# Pharmacological SOS1 inhibitor BI-3406 synergizes with BCR::ABL1 TKIs reducing leukemia stem cells and overcoming imatinib resistance in CML

**DOI:** 10.1038/s41419-026-09170-6

**Published:** 2026-08-12

**Authors:** Rósula García-Navas, Carmela Gómez, Anabel Clemente-Serrano, Belén Zamora-Valdivieso, Sara Pérez-Gutiérrez, Andrea Olarte-San Juan, Alba Díaz-Aguilera, Sonsoles Calvo-Jimenez, Nuria Calzada, Alberto Fernández-Medarde, Magdalena Sierra, Fermín Sánchez-Guijo, Dolors Colomer, Robyn L. Schenk, Marco H. Hofmann, Kaja Kostyrko, Eugenio Santos

**Affiliations:** 1 Centro de Investigación del Cáncer, Instituto de Biología Molecular y Celular del Cáncer, CSIC-Universidad de Salamanca and CIBERONC, Salamanca, Spain; 2 Universidad Internacional de Valencia, Valencia, Spain; 3 Departamento de Anatomía e Histología Humanas, Facultad de Medicina, Universidad de Salamanca, Campus Miguel de Unamuno, Salamanca, Spain; 4 IBSAL (Instituto de Investigación Biomédica de Salamanca), Servicio de Hematología, Hospital Universitario de Salamanca, Departamento de Medicina, Universidad de Salamanca, Salamanca, Spain; 5 Hematopathology Section, Pathology Department, Hospital Clínic, Institut d’Investigacions Biomèdiques August Pi i Sunyer (IDIBAPS) , CIBERONC, Barcelona, Spain; 6 Boehringer Ingelheim RCV GmbH & Co KG, Vienna, Austria

**Keywords:** High-throughput screening, Chronic myeloid leukaemia, Apoptosis

## Abstract

Disease persistence and resistance remain major challenges in chronic myeloid leukemia (CML). We evaluated pharmacological SOS1 inhibition with BI-3406, alone or in combination with BCR::ABL1 tyrosine-kinase inhibitors (TKI), across complementary preclinical models. In p210^BCR/ABL^ transgenic mice, BI-3406 plus imatinib was well-tolerated, markedly prolonged survival, improved hematologic indices, reduced splenomegaly, and decreased bone-marrow LSK/hematopoietic stem and progenitor cells compared to either agent alone. In human CML cell lines and primary patient-derived bone-marrow cells, combined therapeutic treatments with BI-3406 and imatinib (standard of care BCR-ABL TKI) or next-generation TKIs showed strong synergistic suppression of cellular proliferation and increased apoptosis relative to single-agent treatment. Remarkably, drug combinations containing BI-3406 also restored TKI sensitivity to primary bone-marrow cell samples derived from a variety of CML patients that had been initially identified as clinically resistant to imatinib. Mechanistically, BI-3406 attenuated RAS/RAC-ERK/AKT signaling, while SOS1 knockdown phenocopied BI-3406-mediated imatinib sensitization, supporting an on-target SOS1-dependent therapeutic effect. Upregulation of the membrane transport protein SLC22A4 following combination treatment may contribute to the rescued TKI sensitivity but is probably not the sole underlying mechanism as BI-3406+imatinib retained strong synergistic activity despite SLC22A4 knockdown. Comparison of the transcriptomic profiles revealed that the combined BI-3406+imatinib drug regimen triggered the strongest repression of oncogenic, metabolic and stemness-associated transcriptional programs in treated CML cells. Consistently, serial replating MethoCult assays functionally confirmed the impairment of clonogenic self-renewal capacity and the depletion of murine LSK and human Lin⁻CD34⁺CD38⁻ stem/progenitor-enriched compartments after dual SOS1 and BCR::ABL1 inhibition. Our data support SOS1 targeting as a rational therapeutic strategy to potentiate TKI efficacy, impair CML stem/progenitor self-renewal, and restore drug responsiveness in clinically relevant TKI-resistant settings.

**Figure:**
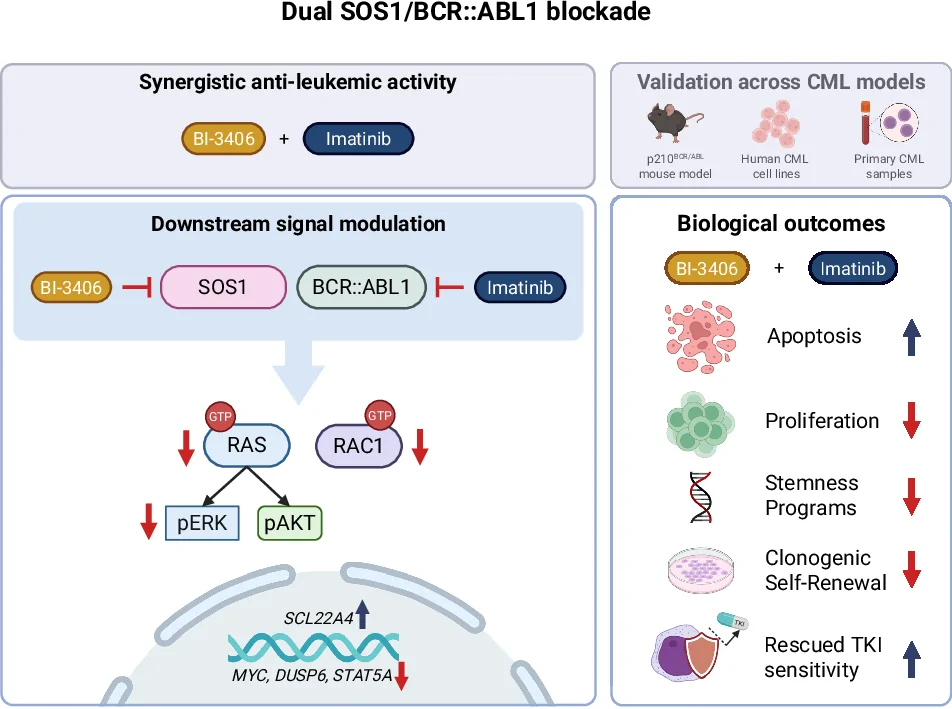

## Introduction

Chronic myeloid leukemia (CML) is driven by the BCR::ABL1 oncoprotein generated by the t(9;22) translocation (Philadelphia chromosome), which confers constitutive tyrosine-kinase activity and promotes myeloid expansion and apoptosis resistance [1]. First-line BCR::ABL1 tyrosine-kinase inhibitors (TKIs) have transformed outcomes in chronic-phase CML; however, a relevant fraction of patients experience intolerance, suboptimal responses, or resistance, often linked to kinase-domain mutations and/or persistence of leukemic stem cells (LSCs), and some progress to advanced phases despite therapy [2–5]. Long-term TKI exposure may also entail vascular adverse events, further limiting durability [6, 7]. These unmet needs support rational combinations with non-ABL targets.

Recent evidence suggests that SOS1, a universal guanine nucleotide exchange factor (GEF) activating RAS proteins, plays a relevant role in CML pathophysiology. Our prior studies in murine tumor models show that SOS1 is required, not only for RAS-driven skin and lung solid tumors [8–10], but also for BCR::ABL1-driven CML. In particular, we showed that SOS1 phosphorylation on Tyr1196 significantly promotes disease development [11] and that SOS1 genetic ablation improves survival and suppresses typical CML markers in mice [12]. Our findings align with other reports [13–15] and with Cancer Dependency Map data, where SOS1 shows the highest gene-dependency score in human CML cell lines [12]. The functional relevance of SOS1 in CML may extend beyond its canonical role in signal transduction, positioning it as a potential therapeutic target. A deeper understanding of the regulatory mechanisms governing SOS1 expression and activity could uncover novel therapeutic strategies to disrupt oncogenic signaling pathways in CML. Moreover, small-molecule SOS1 inhibitors have shown promise in preclinical models, potentially overcoming TKI resistance and improving patient outcomes [13, 14, 16].

Here, we explored SOS1 as a therapeutic target in CML by evaluating the therapeutic efficacy of the pharmacological SOS1 inhibitor BI-3406 in a comprehensive set of preclinical models, including p210^BCR/ABL^ transgenic mice, human CML cell lines, and primary bone-marrow mononuclear cells (BMMNCs) from both imatinib-sensitive and imatinib-resistant CML patients. We found that SOS1 inhibition not only synergistically potentiated the therapeutic effects of BCR::ABL1 TKIs but also enhanced imatinib sensitivity in cells exhibiting a resistant phenotype. Our findings provide new mechanistic and translational clues regarding SOS1 pharmacological targeting in CML and support further clinical studies of SOS1 inhibitors as a novel combinatorial strategy to deepen TKI responses, mitigate LSC-associated persistence and TKI resistance, and ultimately improve patient outcomes.

## Materials and methods

Full protocols, including reagents, antibodies, cell culture media, and oligonucleotide details, are provided in Supplementary Methods and Supplementary Table S1.

### Mouse models and treatment studies

All mouse experiments were approved by the Bioethics Committee of the University of Salamanca (ID: #987) and conducted according to European and Spanish regulations. A transgenic mouse strain harboring the Tec-p210^BCR/ABL^ fusion gene was used as a model for CML as previously described [12, 17]. Disease onset and enrollment were defined by peripheral blood neutrophil counts exceeding 35%. Conditional, inducible *Sos1* knockout mice were generated by crossing the *Sos1*^fl/fl^ strain, in which loxP sites flank exon 10 of the *Sos1* gene, with a tamoxifen-inducible Cre-ERT2 line driven by the endogenous *Polr2a* promoter (RERT locus; Jackson Laboratories Stock #017585), as described previously [18, 19]. These mice were then crossed with Tec-p210^BCR/ABL^ animals to generate the CML model used here [12]*. Sos1* deletion was induced by oral gavage of tamoxifen (75 mg/kg/day for 3 consecutive days) prior to treatment initiation, and deletion efficiency was confirmed by PCR. Mice were housed under specific-pathogen-free (SPF) conditions and monitored daily for body weight, general appearance, posture, activity, and breathing. Pre-specified humane endpoint criteria indicative of advanced leukemic burden (i.e., severe leukocytosis, marked splenomegaly, labored breathing, or >20% body-weight loss) were applied uniformly across treatment arms, and animals were euthanized when these criteria were met. No specific clinical signs compatible with acute non-hematologic organ toxicity were observed under any treatment regimen.

For treatment studies, mice were randomly assigned and received either BI-3406 (50 mg/kg, twice daily) [10], imatinib (100 mg/kg, once daily) [20], or the combination, administered by oral gavage for five consecutive weeks. Disease burden and treatment efficacy were assessed by daily body weight, serial hematology, survival analysis, organ weights, histopathology, and blood/bone-marrow immunophenotyping (flow cytometry). Bone-marrow hematopoietic stem/progenitor cells (HSPCs) were characterized as Lin^−^/Sca-1^+^/c-Kit^+^ (LSK) populations [21].

### Cell lines and patient-derived samples

Human CML cell lines K562 and KCL22, and murine Ba/F3-p210^BCR/ABL^ cells (including T315I and E255K mutants), were cultured in RPMI-1640 supplemented with 10% FBS. Primary bone-marrow mononuclear cells (BMMNCs) were obtained from ten anonymized CML patients after written informed consent under protocols approved by the Complejo Asistencial Universitario de Salamanca CEIm (PI2023101436) and the Hospital Clínic de Barcelona CEIm (HCB/2022/1166). For CML samples, we included adult patients with chronic-phase BCR::ABL1-positive CML and excluded patients with concomitant hematologic malignancies. Drug sensitivity, apoptosis (Annexin V staining), and synergy assays were performed for combination treatments. Cell proliferation was monitored using the Incucyte SX5 Live-Cell Analysis System, and IC50 values and ZIP synergy scores were calculated using SynergyFinder 3.0 (RRID:SCR_019318) [22].

### Molecular and transcriptomic analyses

RAS, MRAS, and RAC activation was quantified using specific GST-RBD pull-down assays [23] in CML cell lines following BI-3406 and imatinib treatment. General protein expression was determined by Western immunoblotting.

RNA-sequencing (RNA-seq) was performed on total RNA from murine and human BMMNCs. Libraries were prepared and sequenced on an Illumina NovaSeq 6000. Differential gene expression analysis used DESeq2 (FDR < 0.05). Functional enrichment analyses employed Enrichr (Reactome, Kinase, and Transcription Factor libraries). Data are deposited in GEO (GSE303378). Quantitative RT-qPCR was used for validation, normalizing to 18S rRNA or B2m.

### Statistical analysis

Data are presented as mean ± SEM from a minimum of three biological replicates. Comparisons were made using One-way ANOVA with Tukey’s post hoc test. Survival was analyzed by the Kaplan–Meier method with the log-rank test. A *p*-value < 0.05 was considered statistically significant.

### Ethics

Primary bone-marrow mononuclear cells (BMMNCs) were obtained from ten anonymized CML patients after written informed consent under protocols approved by the Complejo Asistencial Universitario de Salamanca CEIm (PI2023101436) and the Hospital Clínic de Barcelona CEIm (HCB/2022/1166). All mouse experiments were approved by the Bioethics Committee of the University of Salamanca (#987) and conducted at its NUCLEUS animal facility in accordance with European (2007/526/CE) and Spanish (RD1201/2005 and RD53/2013) guidelines for animal care and experimentation.

## Results

### In vivo tolerability and therapeutic efficacy of SOS1 inhibitor BI-3406 in a murine model of p210^BCR/ABL^-induced CML

**Fig. 1:**
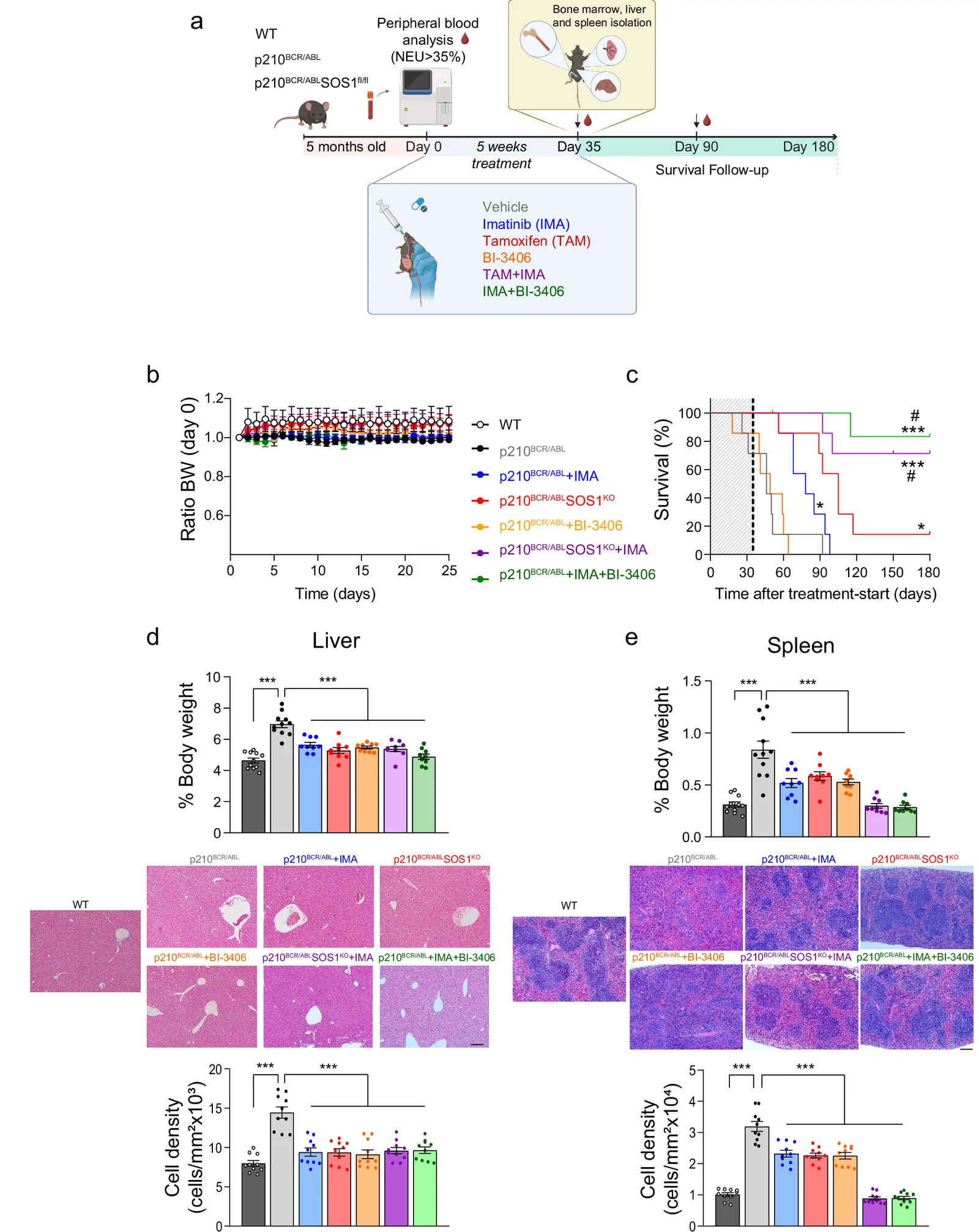
Evaluation of CML progression in p210^BCR/ABL^ mice upon treatment with SOS1 inhibitor BI-3406 and/or BCR/ABL inhibitor imatinib. **a** Schematic overview of treatment regimens including genetic SOS1 ablation (SOS1^KO^) or pharmacological inhibition with BI-3406, administered alone or in combination with the BCR/ABL inhibitor imatinib (IMA). Treatments were initiated at 5–6 months of age, when peripheral blood neutrophil levels exceeded 35% in p210^BCR/ABL^ mice, indicating CML onset. Two parallel experimental arms included (i) a 5-week treatment + endpoint analysis to evaluate body weight, splenomegaly, hepatomegaly, and histopathology, and (ii) a long-term survival follow-up, in which animals underwent the indicated treatments for 5 weeks and were then monitored until humane endpoint and sacrifice. Created in BioRender. García-Navas, R. (2026) https://BioRender.com/ua1hepx. **b** Body-weight evolution over the 25-day treatment period, expressed as a ratio relative to baseline (day 0). None of the treatment regimens significantly impacted body weight, indicating overall tolerability. **c** Kaplan–Meier survival analysis of the different treatment cohorts. The shaded area indicates the initial treatment period (5 weeks), with the dashed line marking the timing of treatment cessation. Treatment conditions and resulting median survival data were as follows: vehicle-treated p210BCR/ABL mice (*n* = 7; median survival, 46 days; reference group); IMA (*n* = 7; median survival, 78 days; HR, 0.3347; 95% CI, 0.05–0.97); SOS1KO (*n* = 7; median survival, 105 days; HR, 0.2481; 95% CI, 0.06–0.91); BI-3406 (*n* = 7; median survival, 49 days; HR, 1.005; 95% CI, 0.26–5.66); SOS1KO + IMA (*n* = 7; median survival, >180 days; HR, 0.114; 95% CI, 0.02–0.48); and IMA + BI-3406 (*n* = 7; median survival, >180 days; HR, 0.06343; 95% CI, 0.01–0.28). Kaplan–Meier curves were compared using the log-rank test. Asterisks shown on the graph indicate comparisons versus vehicle-treated p210BCR/ABL mice (*p < 0.05, **p < 0.01, ***p < 0.001), and # indicates that the combination treatment was significantly superior to the corresponding monotherapy arms. Exact *p*-values are provided in Supplementary Table S2. Quantification of liver (**d**) and spleen (**e**) weights at endpoint, expressed as a percentage of total body weight. Representative H&E-stained liver and spleen sections are shown below the corresponding graphs. Cell-density quantification of H&E-stained sections was performed using QuPath and is expressed as cells/mm². Scale bar, 200 μm. Data represent mean ± SEM from at least three independent mice per group. Statistical analysis was performed using one-way ANOVA with Tukey’s post-test; *p < 0.05, **p < 0.01, ***p < 0.001.

We first investigated the in vivo tolerability and therapeutic efficacy of SOS1 inhibition in the Tec-p210^BCR/ABL^ mouse model of CML [12, 17] (Fig. 1). p210^BCR/ABL^ mice (at 5 months of age, indicating disease onset) were evaluated using two approaches: pharmacological, with BI-3406, imatinib, or their combination; and genetic, using SOS1 deletion (SOS1KO) (Fig. 1a). Subsequently, we assessed the impact on survival, body weight, and CML-associated organomegaly (Fig. 1b–e).

Body-weight measurements throughout the treatment period showed no significant differences across all treatment groups, indicating good tolerability (Fig. 1b). Kaplan–Meier survival analysis (Fig. 1c) showed a median survival of 46 days in vehicle-treated p210BCR/ABL mice. Tamoxifen-induced SOS1 genetic ablation alone prolonged median survival to 105 days, whereas imatinib monotherapy extended it to 78 days, and BI-3406 monotherapy did not improve survival compared with vehicle-treated mice (49 days). In contrast, combining imatinib with either SOS1 deletion or BI-3406 produced significant improvements in survival, with 85.7% of animals in both combination groups remaining alive at the end of the 180-day observation period (Fig. 1c and Supplementary Table S2). These results indicate that while SOS1 inhibition alone is insufficient to confer full survival benefit, its combination with BCR::ABL1 inhibition produces a robust and synergistic therapeutic effect in vivo.

CML-associated organomegaly [24, 25] was ameliorated by treatment. Both agents reduced splenomegaly and hepatomegaly, with the combination producing the greatest spleen shrinkage and restoration of splenic architecture, while liver histology was largely unchanged (Fig. 1d, e).

**Fig. 2:**
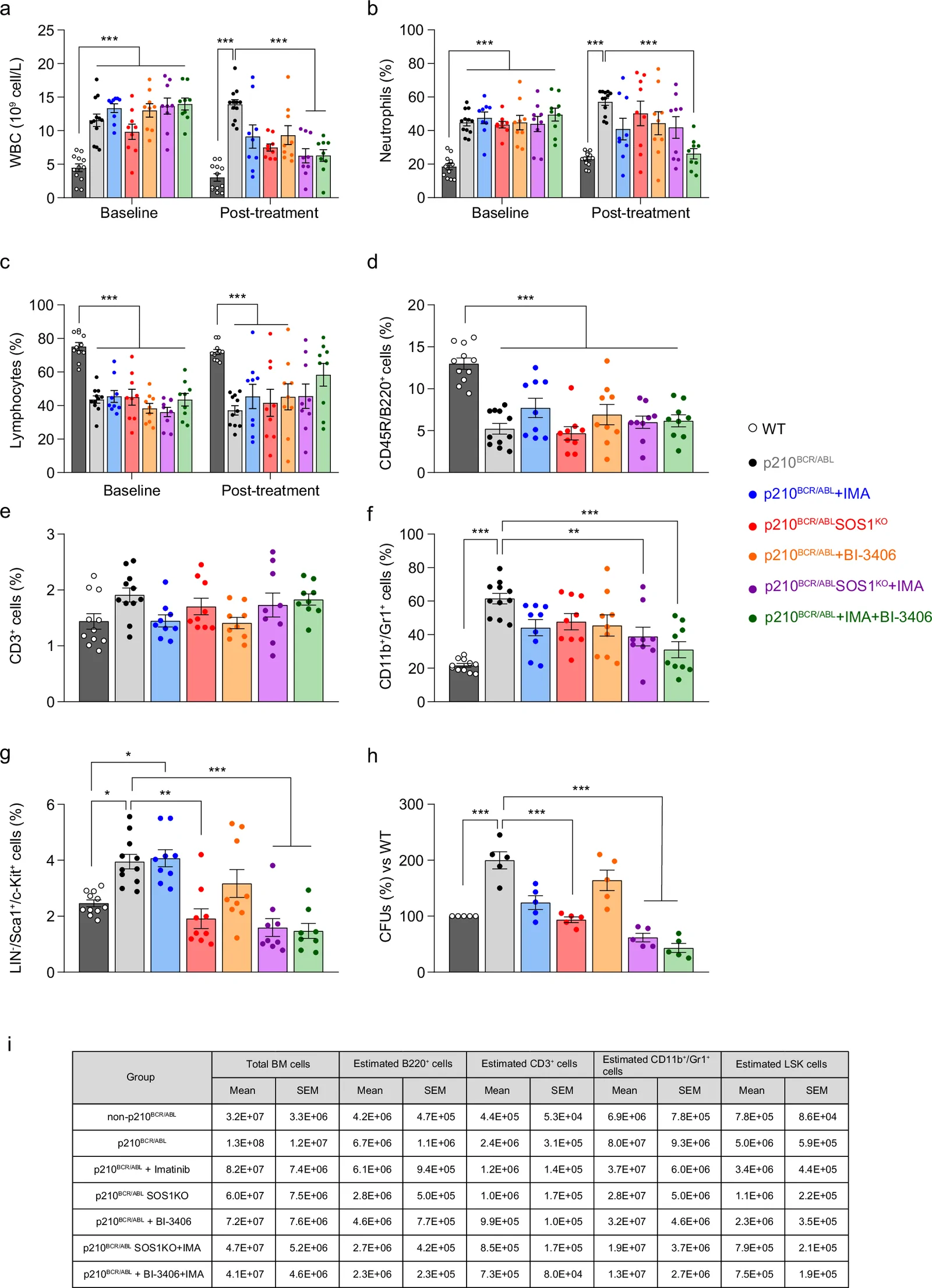
Evaluation of responses in peripheral blood and bone marrow of p210^BCR/ABL^ CML mice treated with SOS1 and/or BCR/ABL inhibition. Peripheral blood parameters before and after treatment. White blood cell (WBC) counts (**a**), neutrophil % (**b**), and lymphocyte % (**c**) were measured by automated hemocytometry. Flow-cytometry analysis of bone-marrow populations: CD45R⁺/B220⁺ B cells (**d**), CD3⁺ T cells (**e**), CD11b⁺Gr-1⁺ cells (**f**), and Lin⁻Sca-1⁺c-Kit⁺ LSK cells (**g**). **h** Clonogenic capacity of bone-marrow mononuclear cells assessed by CFU assay. **i** Estimated absolute bone-marrow cell numbers were calculated by integrating total bone-marrow cellularity with the corresponding flow-cytometry frequencies shown in (**d**–**g**). Full individual mouse values are provided in Supplementary Table S6. Data shown as mean ± SEM (*n* = 9–11 mice/group). Statistical analysis by one-way ANOVA with Tukey’s post-test; *p < 0.05, **p < 0.01, ***p < 0.001.

The impact of single and/or combined inhibition of SOS1 and the BCR::ABL1 kinase on different cell populations from peripheral blood and bone marrow that are relevant for CML pathogenesis was also analyzed (Fig. 2, Supplementary Fig. S1). The therapeutic combination of SOS1 inhibition with imatinib treatment was uniquely effective, resulting in the highest, statistically significant reduction of both total White Blood Cell (WBC) count and percentage of neutrophils in peripheral blood compared to baseline values in other experimental groups (Fig. 2a–c). Longitudinal follow-up further revealed that only the dual inhibition regimen was able to sustain these reductions two months after treatment withdrawal, whereas imatinib monotherapy was associated with hematologic relapse and BI-3406 alone was ineffective, with rapid disease progression (Supplementary Fig. S1a). These findings align with the notion that SOS1 targeting impairs leukemic cell proliferation and survival and indicate that combined SOS1 and BCR::ABL1 blockade achieved superior and more durable disease-burden control compared with either monotherapy.

Bone-marrow samples from the different experimental groups were analyzed by multicolor flow cytometry to quantify major immune and progenitor cells (Fig. 2d–g). B-cell frequencies (CD45R⁺/B220⁺) were markedly reduced in all p210^BCR/ABL^ groups compared to WT controls regardless of treatment (Fig. 2d), whereas T-cell frequencies (CD3⁺) showed no significant variation across treatment groups (Fig. 2e). This pattern is consistent with the known biological features of the murine Tec-p210BCR::ABL1 model, in which leukemic transformation preferentially affects myeloid and B-lineage cells, whereas T cells remain relatively under-represented [17, 26]. The myeloid cell population quantified as CD45⁺CD11b⁺Gr-1⁺ cells was most reduced by combinations (Fig. 2f).

Hematopoietic stem/progenitor cell (HSPC) analysis was performed within the lineage-negative (Lin⁻: CD3⁻, B220⁻, Gr-1⁻, CD11b⁻, Ter119⁻) fraction, identifying LSK cells as Sca-1⁺, c-Kit⁺ [21]. The combination of SOS1 inhibition with imatinib significantly reduced LSK frequencies compared to all other groups (Fig. 2g), whereas monotherapies produced only modest effects.

To complement these frequency-based analyses, we integrated total bone-marrow cellularity with the corresponding flow-cytometry frequencies to estimate the absolute numbers of the major bone-marrow cell populations analyzed in Fig. 2d–g. This analysis confirmed that dual SOS1 and BCR::ABL1 inhibition decreased the estimated, absolute number of LSK cells, supporting a size reduction of the stem/progenitor-enriched compartment rather than a merely proportional change in bone-marrow composition (Fig. 2i; Supplementary Table S6).

Given the role of the spleen as a major reservoir for leukemic stem and progenitor cells in CML [24, 27], we assessed whether the transcriptional profile of stemness-associated genes in this organ was altered by treatment. As expected, *Hoxa9, Kit, Runx1*, and *Myb* were markedly upregulated in the spleen, whereas *Sca-1* and *Meis1* showed no significant differences (Supplementary Fig. S1b). We then evaluated these genes in treated p210^BCR/ABL^ mice. Consistent with the phenotypic depletion of LSK cells (Fig. 2g), combined BI-3406 and imatinib treatment reduced *Hoxa9, Kit, Runx1*, and *Myb* compared to vehicle or either monotherapy. In contrast, *Sca-1* and *Meis1* expressions remained unchanged (Supplementary Fig. S1c).

In line with the above observations, combined SOS1 and BCR::ABL1 inhibition also significantly reduced the clonogenic capacity of bone-marrow cells from the dual-treated mice in comparison to vehicle-treated or single-agent-treated controls (Fig. 2h).

### Therapeutic efficacy of the SOS1 inhibitor BI-3406, alone or in combination with BCR::ABL1 inhibitors such as imatinib, in human CML cell lines

**Fig. 3:**
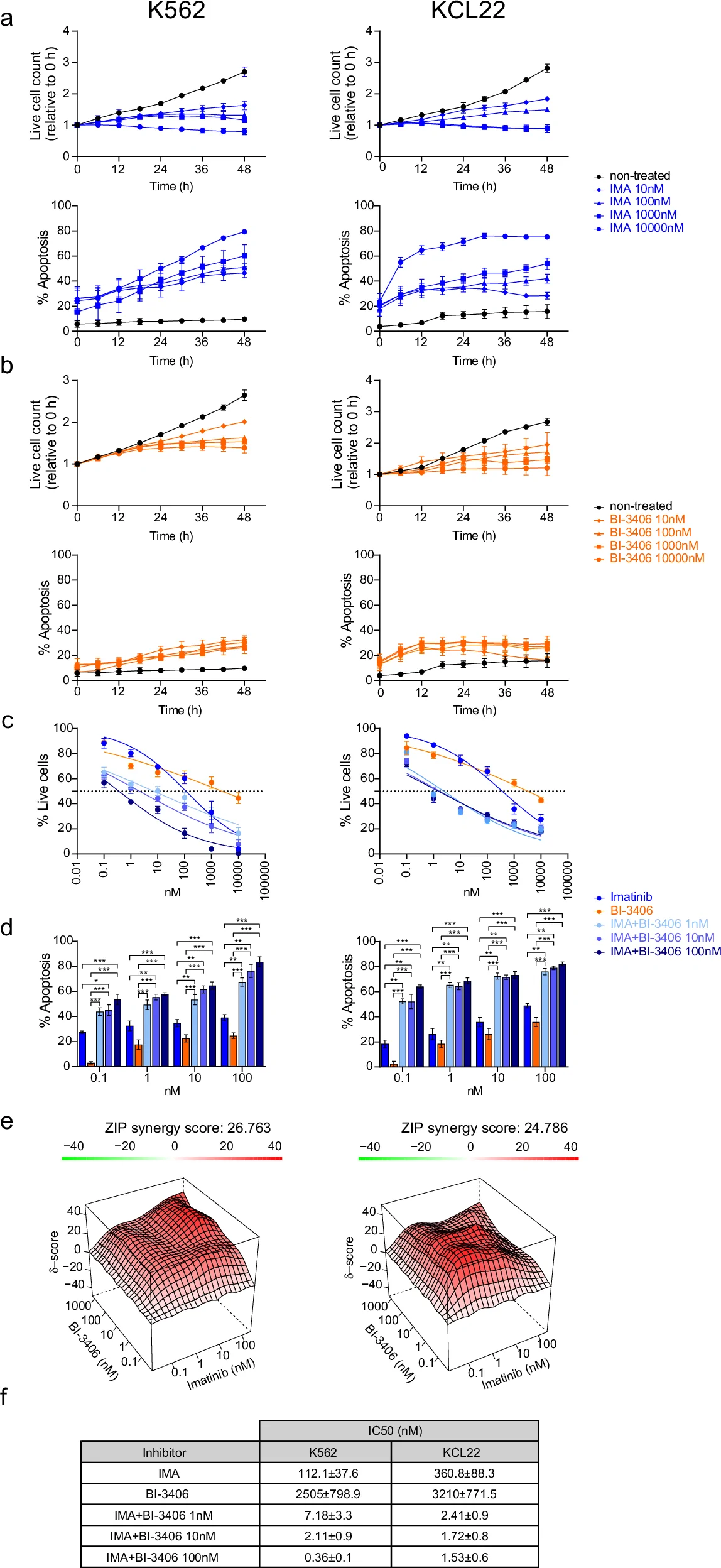
BI-3406 and imatinib reduce viability and induce apoptosis in K562 and KCL22 CML cell lines in a dose- and time-dependent manner. Time-course analysis of cell proliferation (**a**) and Apoptosis analysis (% Annexin V⁺ cells) (**b**) in K562 and KCL22 cells treated with increasing concentrations of imatinib (10–10,000 nM, blue) or BI-3406 (10–10,000 nM, orange) for up to 48 h. Cell viability was measured by live object count normalized to time 0, and apoptosis was assessed by Annexin V staining. Dose-response viability curves (**c**) and Annexin V apoptosis assays (**d**) after 48 h treatment as indicated with imatinib alone (0.1–10,000 nM) or in combination with fixed doses of BI-3406 (1, 10, or 100 nM). **e** Synergy analysis using the ZIP scoring algorithm. 3D surface plots estimating synergy between BI-3406 and imatinib in both CML cell lines, with ZIP scores >20 indicating strong combinatorial effects. **f** Summary table of IC_50_ values (mean ± SD) for imatinib calculated after 48 h co-treatment of both K562 and KCL22 cell lines. IC₅₀ values were calculated using non-linear regression (four-parameter logistic model) in GraphPad Prism v8.0. Data are mean ± SEM from at least three technical and biological replicates. Statistical significance was assessed by one-way ANOVA with Tukey’s post-test; *p < 0.05; **p < 0.01; ***p < 0.001.

We also evaluated the biological impact of BI-3406 when used as a single agent or in combination with imatinib and other later-generation anti-CML TKI drugs on the proliferative and apoptotic rates of human CML cell lines K562 and KCL22 (Fig. 3, Supplementary Fig. S2).

BI-3406 monotherapy was less effective than imatinib in reducing proliferation or inducing apoptosis (Fig. 3a, b). However, BI-3406 treatment increased sensitivity to imatinib, decreasing proliferation and enhancing cell death (Fig. 3c, d) in both model cell lines. This was evident from the significantly lower IC_50_ values calculated for imatinib when combined with BI-3406 (Fig. 3f).

The benefit of BI-3406 as a combination partner was not limited to imatinib. Similar reductions in IC50 values were observed when BI-3406 was combined with second- and third-generation TKIs, including dasatinib, nilotinib, and ponatinib (Supplementary Fig. S2a). Furthermore, synergy scores exceeded 10 for most combinations, supporting a broadly cooperative interaction between BI-3406 and the BCR::ABL1 TKIs tested. In KCL22 cells treated with dasatinib plus BI-3406, the overall ZIP score was low, although localized synergistic activity was observed within specific concentration ranges (Fig. 3e, Supplementary Fig. S2b).

To further assess whether the pharmacological effects of BI-3406 were consistent with SOS1 target engagement, we performed siRNA-mediated SOS1 knockdown in K562 and KCL22 cells (Supplementary Fig. S9). SOS1 depletion efficiently reduced SOS1 expression and reproduced the main sensitizing effect observed with BI-3406, including reduced imatinib IC50 values (Supplementary Fig. S9a–c). These findings further support an on-target contribution of SOS1 inhibition to the observed BI-3406-TKI interaction.

### Therapeutic efficacy of the SOS1 inhibitor BI-3406, as a single agent or in combination with BCR::ABL1 inhibitors such as imatinib, in primary CML patient samples

**Fig. 4:**
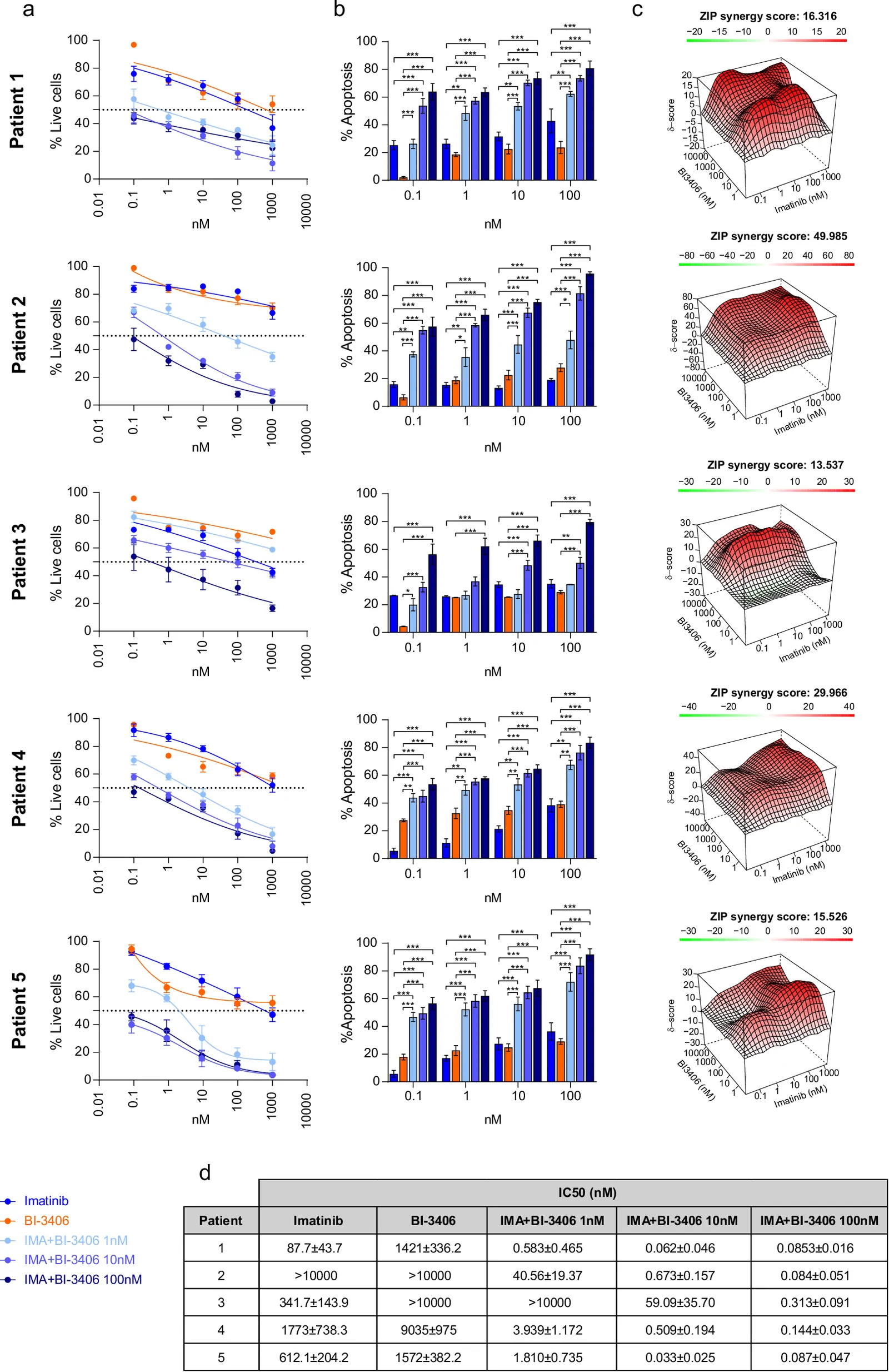
Combination of BI-3406 and imatinib reduces viability and increases apoptosis in primary bone-marrow cells from CML patients. **a** Dose-response curves of BMMNCs from five CML patients treated ex vivo. Cell viability was measured after 48 h and expressed as the percentage of live cells relative to untreated controls. Treatments were: imatinib alone (blue) and BI-3406 alone (orange) over a concentration range of 0.1–1000 nM. Combination treatments consisted of imatinib (over the same 0.1–1000 nM range) combined with a fixed concentration of BI-3406 at 1 nM (light blue), 10 nM (mid blue), or 100 nM (dark blue). **b** Apoptosis analysis (% Annexin V⁺ cells) under the same treatment conditions and time point scale. **c** ZIP synergy analysis of imatinib + BI-3406 combinations for each patient using SynergyFinder. 3D surface plots illustrate strong synergy, with ZIP synergy scores ranging from 13.5 to 49.9. **d** Table summarizing IC_50_ values (nM) for imatinib, calculated after 48 h ex vivo treatment or co-treatment of BMMNCs from all five patients that underwent initial therapy with imatinib; IC₅₀ values were calculated using non-linear regression (four-parameter logistic model) in GraphPad Prism v8.0. Data are mean ± SEM from four technical replicates. Statistical significance was assessed by one-way ANOVA with Tukey’s post-test; *p < 0.05; **p < 0.01; ***p < 0.001.

**Fig. 5:**
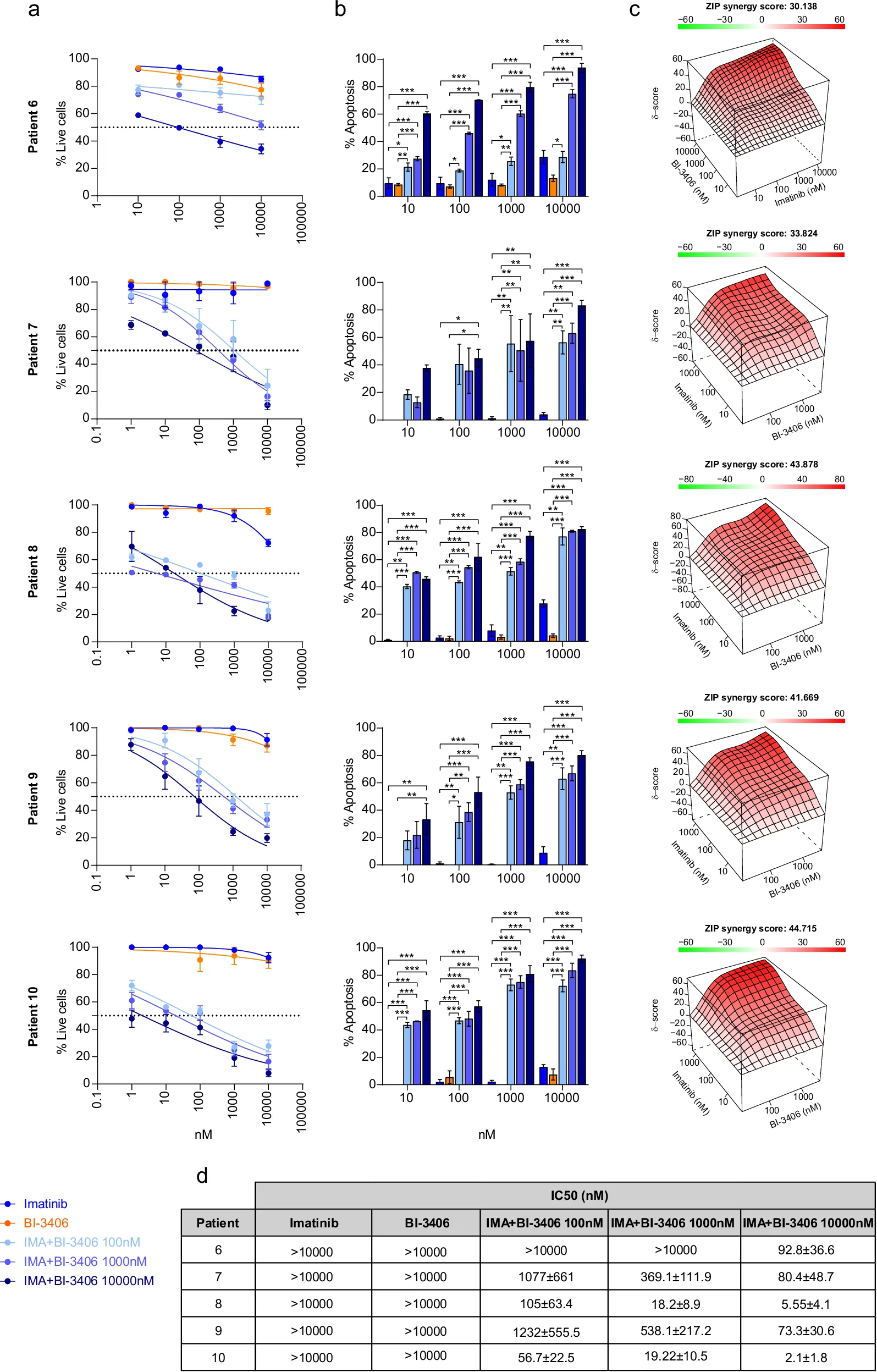
BI-3406 rescues imatinib sensitivity in primary bone-marrow cells from imatinib-resistant CML patients. **a** Dose-response viability curves of BMMNCs from five imatinib-resistant CML patients treated ex vivo for 48 h with imatinib (IMA), BI-3406, or imatinib combined with fixed concentrations of BI-3406. Patient #6 harbored the BCR::ABL1 T315I mutation, whereas patients #7–10 showed clinical resistance to imatinib without detectable BCR::ABL1 kinase-domain mutations by NGS. Cell viability was expressed as the percentage of live cells relative to untreated controls. Imatinib monotherapy was tested over a concentration range of 10–10,000 nM. Combination treatments consisted of imatinib over the same concentration range combined with fixed concentrations of BI-3406 at 100 nM (light blue), 1000 nM (mid blue), or 10,000 nM (dark blue). BI-3406 monotherapy is shown in orange. **b** Apoptosis analysis (% Annexin V⁺ cells) under the same treatment conditions. **c** ZIP synergy analysis of imatinib + BI-3406 combinations for each resistant patient sample using SynergyFinder. 3D surface plots illustrate synergistic interactions between both drugs, with ZIP synergy scores >10 indicating positive drug synergy. **d** Summary table of IC₅₀ values for imatinib, BI-3406, and the indicated imatinib + BI-3406 combinations in each patient-derived sample. IC₅₀ values were calculated using non-linear regression with a four-parameter logistic model in GraphPad Prism v8.0 and are shown as mean ± SD from four technical replicates. Data in (**a**) and (**b**) are shown as mean ± SEM from four technical replicates. Statistical significance was assessed by one-way ANOVA with Tukey’s post-test; *p < 0.05; **p < 0.01; ***p < 0.001.

To confirm the clinical relevance of our findings in CML mice and cell lines, we assessed the impact of BI-3406 as a single agent or in combination with imatinib on primary cell samples from CML patients. For this purpose, we analyzed primary bone-marrow-derived cells from ten anonymized CML patients, including five imatinib-sensitive cases (Fig. 4), one T315I-mutant imatinib-resistant case, and four additional clinically imatinib-resistant cases without detectable BCR::ABL1 kinase-domain mutations (Fig. 5). Supplementary Table S3 summarizes all relevant clinical data of the samples analyzed.

Consistent with prior observations in CML human cell lines [13, 14], BI-3406 as a single agent showed limited therapeutic activity compared to imatinib in BMMNCs, as indicated by relatively high IC_50_ values (>1 μM in most cases; Fig. 4a, d). Notably, combining BI-3406 and imatinib significantly increased cell-death rates compared to the monotherapies (Fig. 4b). This dual-drug regimen consistently achieved strong synergy in all imatinib-sensitive samples, as indicated by very high ZIP synergy scores (range 13.5–49.9; Fig. 4c), supporting the robust cooperative therapeutic potential of SOS1 and BCR::ABL1 co-inhibition in BMMNCs.

Analysis of BMMNCs from imatinib-resistant patient #6, who harbored the BCR::ABL1 T315I mutation and was clinically treated with ponatinib, revealed a strong cooperative effect between BI-3406 and ponatinib. This combination reduced the IC_50_ of ponatinib by one to two orders of magnitude compared with ponatinib alone (Supplementary Fig. S10). As expected, cells from this patient were highly resistant to imatinib monotherapy. Notably, however, the BI-3406+imatinib combination partially restored imatinib sensitivity in these T315I-mutant cells, as shown by increased apoptosis, reduced IC50 values, and high ZIP synergy scores (Fig. 5c). These findings provide proof of concept that SOS1 inhibition can enhance TKI responses even in a kinase-domain mutation-driven resistant context.

To determine whether this sensitizing effect extended beyond a single T315I-mutant case, we next analyzed BMMNCs from four additional CML patients with clinical resistance to imatinib but without detectable BCR::ABL1 kinase-domain mutations. In these mutation-negative resistant samples, imatinib monotherapy showed limited activity, whereas the BI-3406+imatinib combination consistently enhanced drug response, reducing cell viability and increasing apoptosis as compared with either agent alone (Fig. 5a–c). Drug-interaction analyses further supported synergistic or cooperative activity between BI-3406 and imatinib across these clinically resistant samples. Thus, BI-3406-mediated sensitization was not restricted to the single T315I-mutant case but was also observed in clinically resistant CML samples lacking detectable kinase-domain mutations.

We also analyzed in parallel the effect of BI-3406 combinations with other clinically used BCR::ABL1 TKIs, including dasatinib, nilotinib, and ponatinib, on resistant patient-derived samples. These extended analyses showed that BI-3406 also enhanced responses to later-generation TKIs, supporting a broader cooperative interaction, not limited to imatinib, between SOS1 inhibition and BCR::ABL1 blockade (Supplementary Fig. S10).

To further assess the impact of BI-3406 on genetically defined TKI-resistant models, we compared murine Ba/F3 cells expressing p210BCR/ABL with derivative lines harboring the clinically relevant E255K or T315I BCR::ABL1 mutations known to cause TKI resistance in CML [28] (Supplementary Fig. S3). As expected, both mutant lines displayed markedly increased IC50 values for imatinib compared with parental Ba/F3-p210 cells. Consistently, co-treatment with BI-3406 improved imatinib sensitivity in both mutant models. Although Annexin V-based apoptosis assays showed mainly additive effects, metabolic viability assays revealed strong synergy in the E255K and T315I-mutant cells (Supplementary Fig. S3j), supporting the ability of SOS1 inhibition to potentiate imatinib activity in kinase-domain mutation-associated resistant models.

### Impact of BI-3406 treatment on intracellular signaling in CML model cell lines

**Fig. 6:**
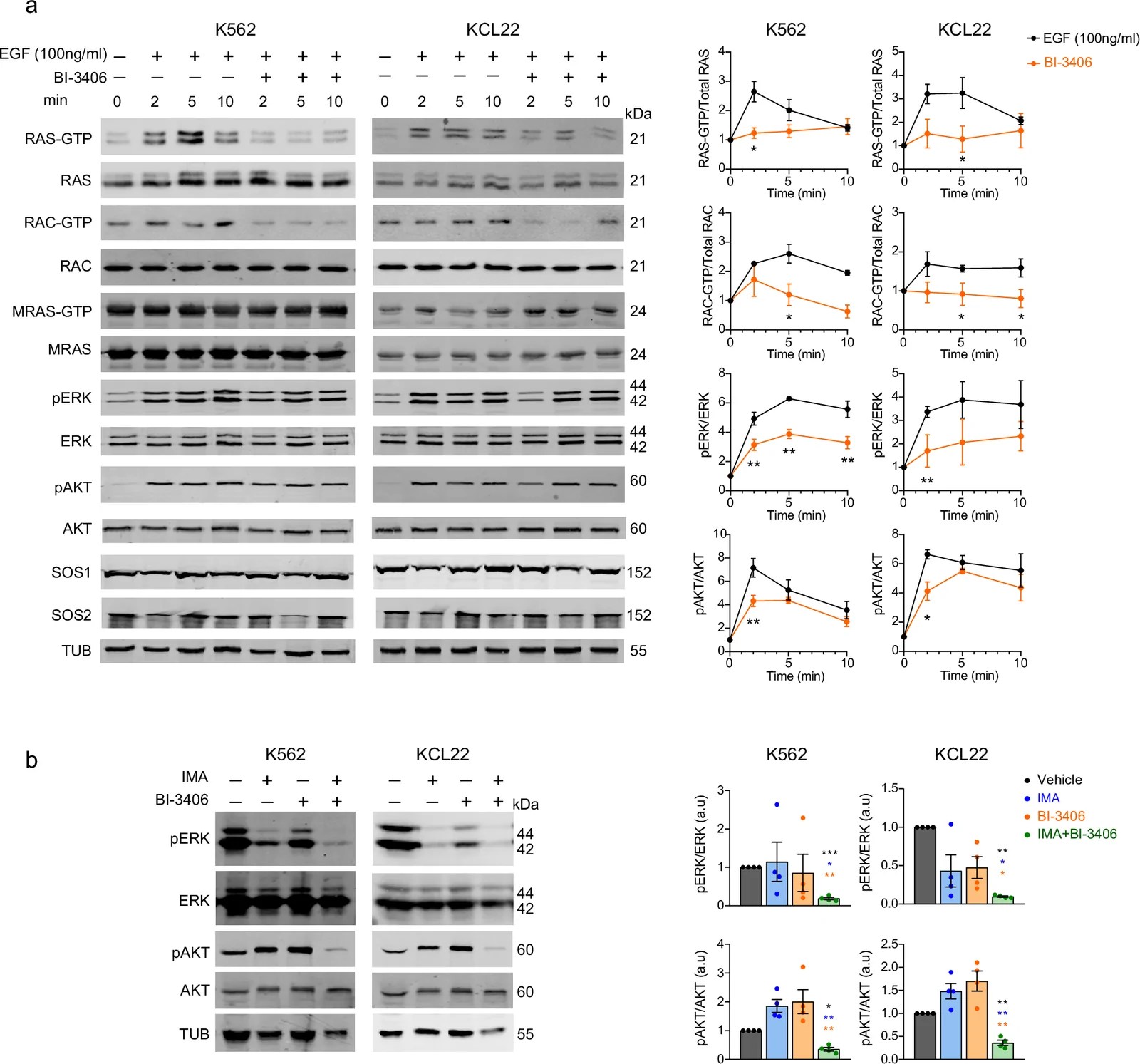
BI-3406 restrains RAS and RAC activation and attenuates downstream ERK and AKT phosphorylation in K562 and KCL22 CML cell lines. **a** Western blot analysis of serum-starved K562 and KCL22 cells stimulated with EGF (100 ng/mL) for 2, 5, or 10 min, either alone or following pre-treatment (2 h) with BI-3406 (1 μM). Active GTP-bound RAS, RAC, and MRAS were assessed by pull-down assays as described in the “Materials and methods” section. Tubulin (TUB) was used as a loading control for all blots. Quantification of RAS-GTP, RAC-GTP, pERK, and pAKT levels normalized to total protein is shown on the right. Graphs represent mean ± SEM from at least three independent experiments. **b** Cells were treated for 24 h with BI-3406 (1 μM), imatinib (IMA, 500 nM), or their combination, and phospho-ERK and phospho-AKT levels were assessed by Western blot. Tubulin was used as a loading control. The blots are representative of at least three independent experiments. Blue asterisks indicate significance vs. imatinib alone; orange asterisks indicate significance vs. BI-3406 alone, and black asterisks indicate significance vs. vehicle-treated alone. Statistical significance was assessed by one-way ANOVA with Tukey’s post-test; *p < 0.05; **p < 0.01; ***p < 0.001.

To explore the impact of the pharmacological SOS1 inhibitor BI-3406 on intracellular signaling, we analyzed the activation kinetics in K562 and KCL22 cell lines (Fig. 6). Consistent with our reports in other systems [9, 10], BI-3406 as a single agent effectively reduced RAS activation and dampened ERK phosphorylation upon EGF stimulation (Fig. 6a). We also observed a slight reduction in pAKT formation following EGF stimulation, indicating some effect on PI3K-dependent signaling (Fig. 6a).

Pull-down assays showed that single-agent BI-3406 treatment also caused a clear drop in RAC-GTP formation in both cell lines after EGF stimulation (Fig. 6a). This is consistent with our previous report showing SOS1 requirement for RAC1 phosphorylation and disease progression [11, 12] and other reports supporting an essential role of RAC1 in CML development [29, 30]. While these data suggest that both RAS and RAC contribute to CML development, no changes in MRAS activation were observed in these CML cell lines under the same experimental conditions (Fig. 6a).

To explore the sustained effects of BI-3406 and its combination with imatinib, we assessed the phosphorylation status of ERK and AKT after extended treatment (24 h) of the same cell lines with EGF (Fig. 6b). The combination induced a strong and sustained reduction of both pERK and pAKT levels. These findings further support a cooperative effect of dual SOS1 and BCR::ABL1 inhibition leading to blockade of key survival and proliferation pathways in CML cells.

We also evaluated the in vivo impact of dual targeting on MAPK signaling in the spleen, a key extramedullary niche in CML [24], by analyzing pERK levels (Supplementary Fig. S4). While BI-3406 monotherapy modestly reduced pERK, imatinib alone significantly increased the proportion of pERK+ cells. Dual SOS1 and BCR::ABL1 inhibition attenuated this imatinib-induced increase and led to lower pERK levels compared to imatinib monotherapy (Supplementary Fig. S4a, b).

### Transcriptional impact of BI-3406 and/or TKI treatment on BM cells of CML mice and patients

**Fig. 7:**
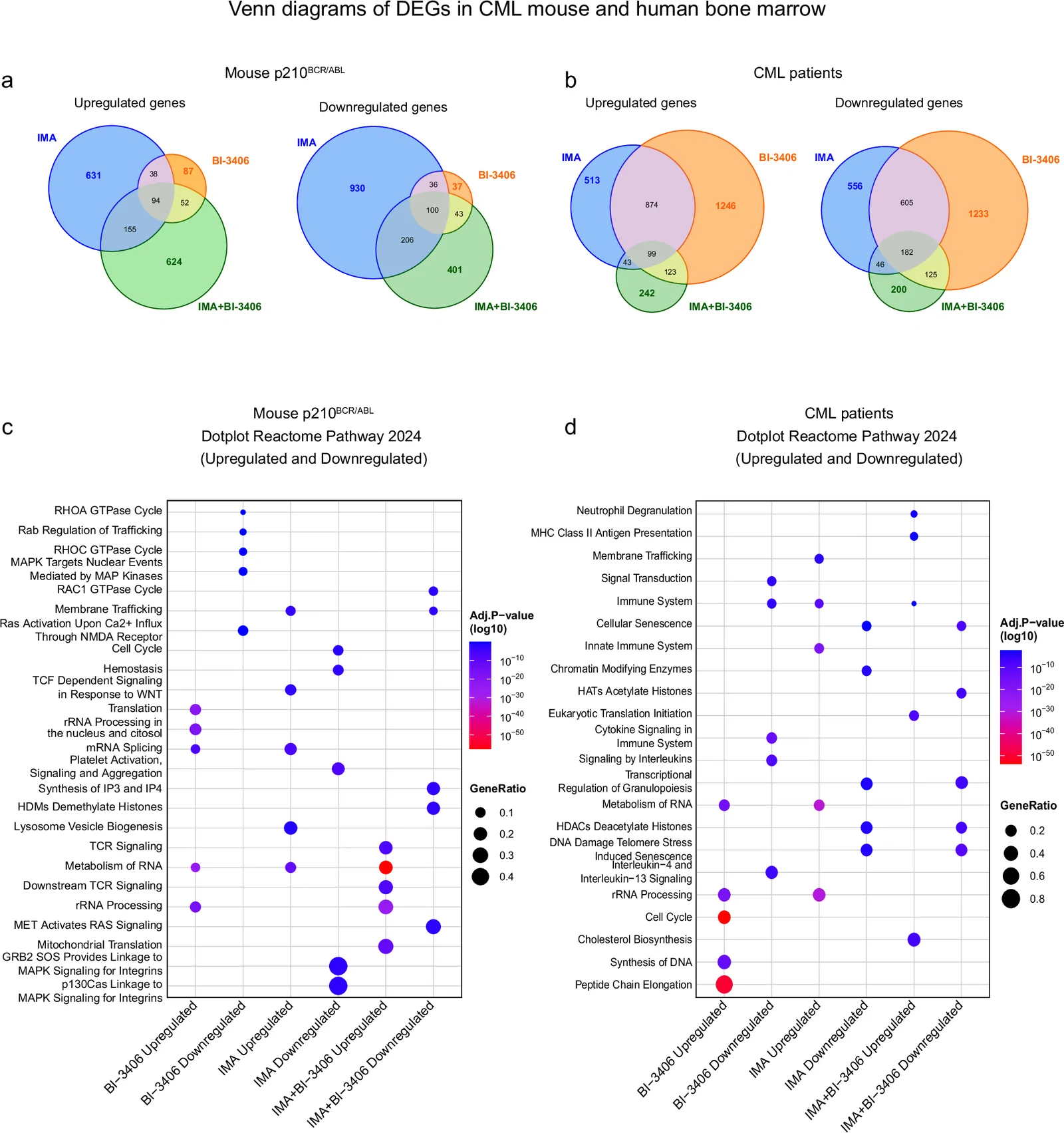
Transcriptomic analysis after single or combined BI-3406 and imatinib treatment in CML bone marrow of mice and patients. Combined treatments induce broader and more convergent transcriptional reprogramming than monotherapies. Venn diagrams showing overlap of differentially expressed gene sets (DEGs) that are upregulated (left) or downregulated (right) in bone-marrow samples from p210^BCR/ABL^ transgenic mice (**a**, left side panels) or CML patients (**b**, right side panels) treated with BI-3406, imatinib (IMA), or their combination (IMA + BI-3406). **c**, **d** Dot plots showing enrichment of signaling pathways (Reactome Pathways 2024 database) for DEGs in the same set of samples as in (**a**, **b**). (**c**) corresponds to mouse bone marrow, and (**d**) corresponds to human patient bone marrow. The size of each dot represents the gene ratio (number of DEGs in the pathway relative to total genes in that pathway), while color reflects the adjusted *p*-value (FDR), with more intense red indicating higher statistical significance.

To elucidate the mechanistic basis for the observed therapeutic synergy, we analyzed the transcriptional profiles using RNA-seq and RT-qPCR (Fig. 7; Supplementary Figs. S5–S8; Supplementary Tables S4 and S5). We focused on BM samples obtained from p210^BCR/ABL^ mice treated in vivo with the drug regimens, and human CML BMMNCs treated ex vivo with BI-3406, imatinib (IMA), or the combination. Unsupervised hierarchical clustering and principal component analysis (PCA) confirmed that each drug regimen induced a distinct transcriptional signature (Supplementary Fig. S5). After identifying genes differentially expressed under each experimental condition (FDR < 0.05; |log2FC| ≥ 1), we performed functional enrichment analyses of the specific transcriptomic patterns linked to single or combined drug treatments in the bone-marrow samples of CML mice or patients (Fig. 7; Supplementary Fig. S6, Supplementary Tables S4 and S5).

In both CML mice and patient samples, BI-3406 monotherapy triggered a compensatory transcriptomic response characterized by upregulation of gene programs related to ribosome biogenesis, rRNA processing, mRNA splicing, and translation initiation (Supplementary Fig. S6). These were accompanied by enrichment of MYC target genes and transcription factors such as TEAD4 and ELK1, indicative of a proliferative stress-adaptive state (Supplementary Tables S4 and S5). This transcriptional pattern was stronger in mice but qualitatively conserved in patient-derived samples (Fig. 7). Notably, qPCR validation of selected transcripts supported these conclusions. For example, *SLC22A4*, a gene associated with amino acid and drug transport [31], which has also been linked to reversion of imatinib resistance [13, 32], was upregulated upon BI-3406 treatment and further increased under the drug combination regimen. Because this association does not formally establish causality, we functionally tested its contribution using siRNA-mediated SLC22A4 knockdown in K562 and KCL22 cells (Supplementary Fig. S9). SLC22A4 depletion modestly reduced imatinib sensitivity, consistent with a contribution of this transporter to imatinib responsiveness. However, IMA+BI-3406 retained strong cooperative activity after SLC22A4 knockdown, suggesting that SLC22A4 contributes to, but does not fully account for, the sensitizing effect of dual SOS1 and BCR::ABL1 inhibition (Supplementary Fig. S9d–g). Furthermore, the expression of MYC and the ERK-responsive DUSP6 gene were both significantly downregulated upon BI-3406 exposure (Supplementary Fig. S8a, b), consistent with attenuation of RAS–MAPK-associated transcriptional outputs (Supplementary Tables S4 and S5).

Imatinib treatment-induced downregulation of canonical oncogenic pathways (MYC and E2F targets, mTORC1 signaling, oxidative phosphorylation, and G2/M checkpoint regulators) in both murine and human samples (Supplementary Tables S4 and S5). Additionally, imatinib treatment upregulated expression of genes involved in vesicle trafficking, lysosome function, and immune-modulatory cytokines, suggesting early immune engagement. These immunometabolic effects were broadly similar across species, although cytokine-linked immune signatures appeared more pronounced in patients (Fig. 7; Supplementary Fig. S6, Supplementary Tables S4 and S5).

Strikingly, the imatinib+BI-3406 combination produced synergistic and more profound transcriptional reprogramming in both models. Shared effects included deeper repression of biosynthetic and mitotic programs, including DNA repair and unfolded protein response, and enhanced downregulation of cell cycle regulators (Fig. 7, Supplementary Fig. S6, Supplementary Tables S4 and S5). Notably, the combination showed the strongest enrichment of inflammatory, interferon/antiviral, and immune-related transcriptional programs, particularly in human patient-derived samples (Fig. 7d). Because these analyses were performed on bulk bone-marrow mononuclear cells, the cellular source of these signatures cannot be definitively assigned. In the murine model, flow-cytometry analysis did not reveal significant changes in CD3+ T-cell frequencies across treatment groups, suggesting that immune-related transcriptional changes are unlikely to simply reflect expansion of the T-cell compartment. Rather, these signatures may reflect treatment-induced transcriptional remodeling within the mixed bone-marrow compartment, potentially involving myeloid, antigen-presenting, progenitor, and/or residual lymphoid populations.

To further gauge the impact of the drug treatment regimens on hematological maturation programs, we next computed gene set-based “Stemness Scores” [33, 34] from RNA-seq using curated transcription factor (TF) [35], surface marker [36], and pathway [37–39] stemness modules, together with LT-HSC and ST-HSC/MPP signatures in mice [40] and Bone-Marrow Map-derived [41] HSC/MPP and GMP-like cell-state signatures in patients (Supplementary Fig. S7; Supplementary Methods). Vehicle-treated mice showed positive enrichment across all stemness modules versus non-leukemic controls, consistent with expansion of HSC/LSC-like programs. Imatinib or BI-3406 alone only partially reduced these scores, whereas their combination reproducibly yielded the lowest values in both murine and human samples, indicating a more effective suppression of HSC/MPP-like transcriptional programs under dual SOS1 and BCR::ABL1 inhibition.

The synergistic effects were validated by RT-qPCR in patient-derived samples (Supplementary Fig. S8). In imatinib-sensitive patients, the combination consistently caused the largest decrease in key leukemic transcripts (*MYC, STAT5A, DUSP6, GATA2*, and *HIF1A*), with *BCR::ABL1* expression reaching its lowest levels. This suppression aligns with convergent reduction of MAPK/ERK (DUSP6 is an ERK-responsive phosphatase [42]) and JAK/STAT outputs, alongside attenuation of stemness (*GATA2*) and hypoxia (*HIF1A*) programs. Also, *SLC22A4* was maximally upregulated.

On the other hand, in patient #6 harboring the imatinib-resistant T315I mutation, imatinib and BI-3406 monotherapies increased *DUSP6* and *MYC* transcripts (compatible with compensatory MAPK output), whereas the imatinib+BI-3406 combination markedly suppressed *DUSP6* and *MYC* transcription below vehicle-treated samples, while also eliciting the highest *SLC22A4* expression (Supplementary Fig. S8b).

Together, our RNA-seq and qPCR data highlight the synergistic ability of imatinib and BI-3406 to repress oncogenic, metabolic, and stemness-associated programs, while inducing transcriptional remodeling of immune/inflammatory pathways. These results suggest that the therapeutic synergy involves, at least in part, suppression of leukemic stemness-associated programs and attenuation of key survival signaling outputs. We therefore next asked whether these transcriptional changes were accompanied by a functional impairment of stem/progenitor self-renewal.

### Functional confirmation of impaired CML stem/progenitor self-renewal after dual SOS1 and BCR::ABL1 inhibition

**Fig. 8:**
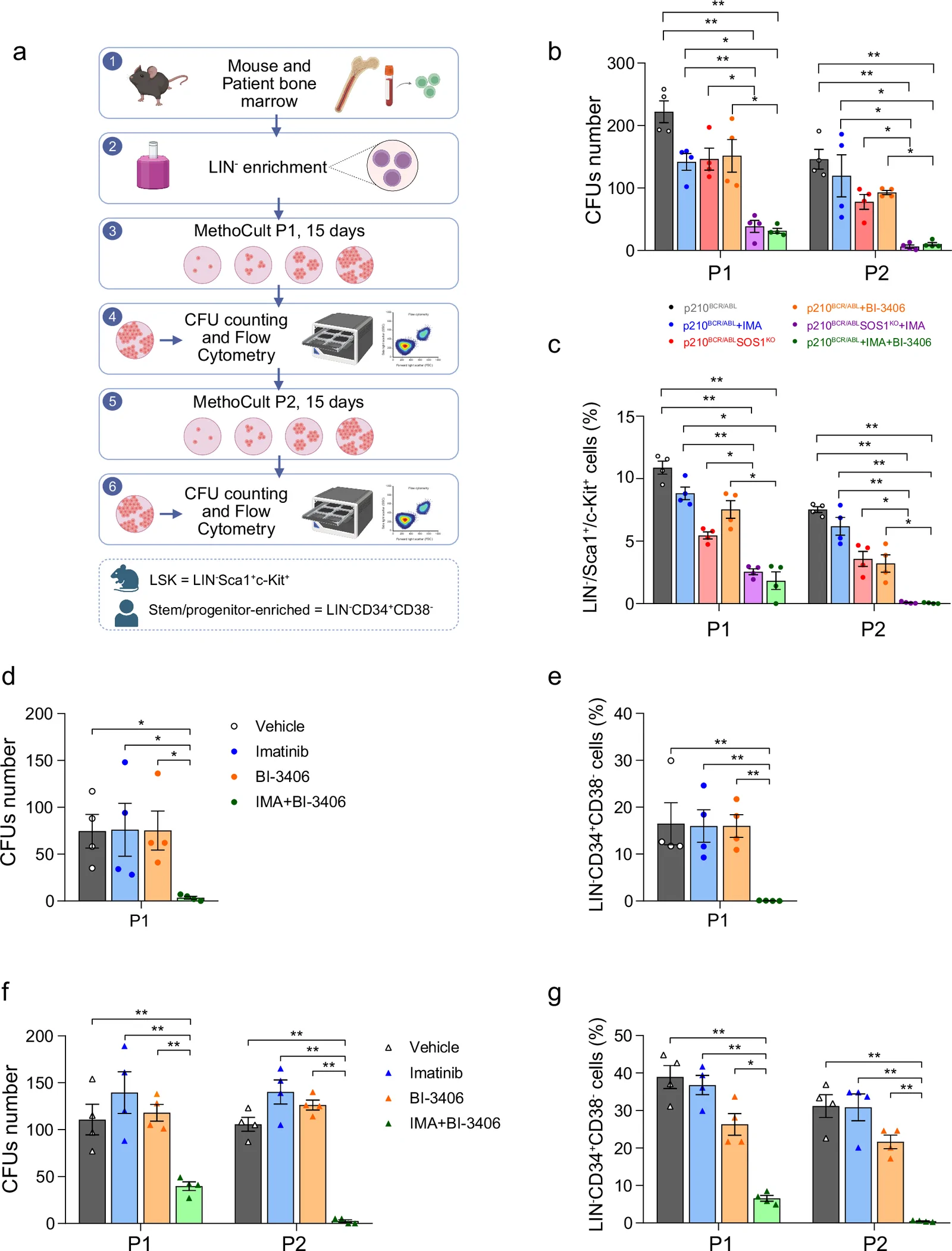
Dual SOS1 and BCR::ABL1 inhibition suppresses serial clonogenic capacity and depletes stem/progenitor-enriched compartments in murine and patient-derived CML samples. **a** Schematic representation of the serial MethoCult replating strategy. Bone-marrow cells from treated p210BCR/ABL mice or primary CML patient samples were subjected to Lin⁻ enrichment and cultured in MethoCult for 15 days. After the first passage (P1), colonies were counted, and cells were analyzed by flow cytometry. Recovered cells were then replated for a second MethoCult passage (P2), followed by CFU counting and flow-cytometry analysis. Murine stem/progenitor-enriched cells were defined as LSK cells (Lin⁻Sca-1⁺c-Kit⁺), whereas human stem/progenitor-enriched cells were defined as Lin⁻CD34⁺CD38⁻ cells. Created in BioRender. García-Navas, R. (2026) https://BioRender.com/ols7zm2. **b** Number of colony-forming units (CFUs) generated after P1 and P2 from Lin⁻ bone-marrow cells isolated from p210BCR/ABL mice treated with vehicle, imatinib (IMA), BI-3406, SOS1KO, SOS1KO+IMA, or IMA+BI-3406. Dual SOS1 and BCR::ABL1 inhibition, either genetically or pharmacologically, markedly reduced serial clonogenic capacity, with minimal colony formation after secondary replating. **c** Flow-cytometry quantification of Lin⁻Sca-1⁺c-Kit⁺ (LSK) cells recovered from MethoCult cultures after P1 and P2 in the same murine treatment groups. Combination-treated cultures showed a marked depletion of the LSK compartment after serial replating. **d** CFU number after P1 in Lin⁻ bone-marrow cells from imatinib-sensitive CML patient samples treated ex vivo with vehicle, IMA, BI-3406, or IMA+BI-3406. Secondary replating (P2) was not performed for combination-treated cultures owing to insufficient cell recovery after P1; therefore, only P1 data are shown. **e** Flow-cytometry quantification of Lin⁻CD34⁺CD38⁻ stem/progenitor-enriched cells recovered after P1 from imatinib-sensitive CML patient-derived MethoCult cultures. **f** CFU number after P1 and P2 in Lin⁻ bone-marrow cells from clinically imatinib-resistant CML patient samples treated ex vivo with vehicle, IMA, BI-3406, or IMA+BI-3406. In resistant samples, monotherapies preserved serial replating capacity, whereas the IMA+BI-3406 combination strongly impaired CFU formation after P1 and almost completely abrogated colony formation after P2. **g** Flow-cytometry quantification of Lin⁻CD34⁺CD38⁻ stem/progenitor-enriched cells recovered after P1 and P2 from clinically imatinib-resistant CML patient-derived MethoCult cultures. Data are shown as mean ± SEM. Each dot represents an individual mouse or patient-derived sample. Statistical significance was assessed by one-way ANOVA followed by Tukey’s post-test; *p < 0.05, **p < 0.01, ***p < 0.001.

To determine whether the transcriptional suppression of stemness-associated programs translated into functional impairment of CML stem/progenitor self-renewal, we performed serial MethoCult replating assays using Lin⁻ stem/progenitor-enriched cells from treated p210^BCR/ABL^ mice and primary CML patient-derived samples (Fig. 8a). In the murine model, Lin⁻ cells isolated from vehicle-treated p210^BCR/ABL^ mice retained robust clonogenic capacity after the first MethoCult passage and continued to generate colonies upon secondary replating. Imatinib or BI-3406 monotherapy produced only partial effects on CFU output and did not profoundly reduce the phenotypic LSK compartment during serial replating. SOS1 genetic ablation reduced LSK recovery, consistent with the requirement of SOS1 for maintenance of the leukemic stem/progenitor compartment, although this effect was less pronounced than that observed with dual SOS1 and BCR::ABL1 inhibition (Fig. 8b, c).

In contrast, both combination strategies, SOS1KO+IMA and BI-3406+IMA, markedly reduced CFU formation after the first MethoCult passage and almost completely abrogated colony formation upon secondary replating (Fig. 8b). Flow-cytometry analysis of replated cultures confirmed a parallel reduction in Lin⁻Sca-1⁺c-Kit⁺ LSK cells, with near-complete loss of this compartment after the second passage in both dual-treatment groups (Fig. 8c).

We next extended these functional analyses to primary CML patient samples. In imatinib-sensitive samples, BI-3406+IMA strongly impaired colony formation already after the first MethoCult passage, and secondary replating was not possible owing to insufficient cell recovery in combination-treated cultures (Fig. 8d). Consistently, flow-cytometry analysis showed a marked depletion of the Lin⁻CD34⁺CD38⁻ stem/progenitor-enriched compartment after BI-3406+IMA treatment (Fig. 8e).

In clinically imatinib-resistant samples lacking detectable BCR::ABL1 kinase-domain mutations, monotherapies showed only modest effects and largely preserved serial replating capacity. However, the BI-3406+IMA combination markedly reduced CFU formation after the first passage and almost completely abrogated secondary replating capacity (Fig. 8f). Flow-cytometry analysis further revealed a profound depletion of the Lin⁻CD34⁺CD38⁻ stem/progenitor-enriched compartment in combination-treated cultures, particularly after serial replating (Fig. 8g).

Together, these serial replating and flow-cytometry data provide functional evidence that dual SOS1 and BCR::ABL1 inhibition impairs the self-renewal-associated clonogenic potential of CML stem/progenitor-enriched populations in both murine and human settings.

## Discussion

We demonstrated previously that SOS1 is critical for CML by showing that ABL-mediated phosphorylation of SOS1 contributes to BCR::ABL1-driven leukemogenesis [11] and that SOS1 genetic ablation suppresses the main pathological hallmarks of CML in p210^BCR/ABL^ mice [12]. Consistent with other reports involving SOS1 inhibition [13–15], these observations suggested that SOS1 is a promising therapeutic target in CML. To test this hypothesis, here we evaluated the impact of BI-3406, a specific pharmacological inhibitor of SOS1, on various biological models of CML, including p210^BCR/ABL^-expressing mice, human CML cell lines, and primary bone-marrow cells from imatinib-sensitive and imatinib-resistant CML patients.

In vivo, BI-3406, alone or combined with imatinib, showed no significant impact on body weight or overt clinical signs of acute toxicity, suggesting that a therapeutic window exists for SOS1 inhibitor-based therapy. BI-3406 monotherapy significantly improved splenomegaly, mirroring the effects of SOS1 genetic ablation [12] but did not provide a survival benefit compared with vehicle, suggesting that partial and transient pharmacological SOS1 inhibition, at the dose and schedule used here, was insufficient to durably control leukemic burden. The apparent discrepancy between the superior efficacy of genetic SOS1 ablation and the more limited monotherapy potency of BI-3406 most likely reflects pharmacokinetic and pharmacodynamic differences between the two approaches. Inducible genetic deletion produces sustained SOS1 loss after tamoxifen administration, whereas pharmacological inhibition via oral dosing may provide time-limited and potentially incomplete target coverage over a 24 h dosing interval in the bone marrow and splenic microenvironment. This is consistent with published data showing that BI-3406 achieves meaningful antitumor activity in KRAS-driven solid tumors with continuous dosing but requires combination with RAS-pathway inhibitors to achieve its full therapeutic potential [10, 43]. In the CML context, where leukemic cells are maintained in protected niches with potentially reduced drug penetrance [44, 45], the threshold required for a durable monotherapy effect may not have been reached. Importantly, the on-target nature of BI-3406 activity in this setting is supported by siRNA-mediated SOS1 knockdown experiments, which reproduced the key sensitizing effects observed pharmacologically. Furthermore, the fact that BI-3406 combined with imatinib achieves outcomes comparable to genetic SOS1 ablation combined with imatinib strongly supports an on-target, SOS1-dependent contribution to the observed synergy.

Interestingly, while imatinib alone offered only slightly better therapeutic benefits, the combination of BI-3406 and imatinib dramatically enhanced anti-CML activity, revealing robust synergy and supporting simultaneous targeting of SOS1 and BCR::ABL1 as a potential therapeutic strategy in CML. This is further supported by our in vivo observations in CML mice, indicating that the combined treatments, including BI-3406, curtail the LSK (Lin⁻, Sca-1⁺, c-Kit⁺) compartment in the bone marrow while simultaneously reducing splenomegaly and downregulating expression of specific stemness-associated markers in the spleen, an organ long recognized as an important reservoir for leukemic stem and progenitor cells in CML [24, 27].

In CML cell lines and primary bone-marrow cultures, BI-3406 synergized with imatinib and later-generation TKIs, yielding stronger suppression of proliferation and clonogenicity and increased apoptosis compared with single agents. Notably, combinations restored drug sensitivity in samples harboring imatinib-resistance mutations while retaining strong activity in imatinib-sensitive contexts, supporting SOS1 blockade as a broadly applicable TKI partner strategy across diverse resistance contexts.

Another remarkable observation was the ability of BI-3406-containing combinations to enhance TKI sensitivity in resistant CML contexts. In a primary bone-marrow sample from a patient carrying the T315I mutation, BI-3406 potentiated ponatinib activity and partially restored imatinib sensitivity, providing proof of concept that SOS1 inhibition can improve TKI responses even in a kinase-domain mutation-driven resistant setting. Importantly, a similar sensitizing effect was also observed in additional clinically imatinib-resistant, mutation-negative primary CML samples, supporting the relevance of SOS1 inhibition beyond the single T315I-mutant case.

SLC22A4 upregulation may contribute to this sensitization phenotype, as this transporter has been previously linked to imatinib responsiveness [13, 32]. However, our SLC22A4 knockdown experiments indicate that this mechanism is not sufficient to account for the observed BI-3406+imatinib therapeutic synergy. SLC22A4 depletion modestly reduced imatinib sensitivity, supporting a functional contribution of this transporter, but did not abrogate the cooperative effect of BI-3406 and imatinib. These observations suggest that the drug re-sensitization of TKI-resistant CML cells may be a multifactorial process involving both SLC22A4 upregulation and other components arising from broader SOS1-dependent transcriptional rewiring.

Mechanistically, single-agent BI-3406 treatment of CML cell lines reduced the levels of activated RAS-GTP and pERK produced after EGF stimulation, confirming that, as in other solid tumor systems [10, 43], SOS1-dependent RAS signaling is a relevant therapeutic target in CML. Consistent with prior reports implicating SOS1 phosphorylation and RAC1 activation in CML pathogenesis [11, 46], BI-3406 also reduced RAC-GTP levels and modestly attenuated pAKT, suggesting broader effects on PI3K-dependent signaling. In this context, no modification of MRAS-GTP was detected under the same experimental conditions, suggesting that SOS1–MRAS interactions may not represent a major functional axis in the CML cell lines studied here, although this mode of action was described in other indications [47, 48]. Notably, consistent with the known ability of imatinib to downregulate RAS/ERK and STAT5 signaling [49], the combination of imatinib + BI-3406 triggered deeper, synergistic reductions of both the pERK/ERK and the pAKT/AKT ratios, suggesting that concurrent dampening of the RAS/RAC→ERK/AKT axis is important for the observed synergistic therapeutic behavior [47].

Consistent with these mechanistic observations, transcriptomic analysis of CML cells treated with BI-3406 and/or imatinib revealed significant transcriptional reprogramming, including robust repression of gene signatures, pathways and markers relevant for proliferation (e.g., *MYC, DUSP6*) as well as for leukemic cell survival (e.g., *MYC, STAT5A*, E2F targets, G2/M checkpoint regulators, mTORC1 signaling) [42, 50, 51]. In particular, the combined treatments produced broader and more profound transcriptional changes than the single agents, further indicating a cooperative transcriptional impact beyond that of either monotherapy. In line with this, our gene set-based stemness and hematopoietic cell-state scoring in murine [40] and human [34, 41] CML bone marrow showed that BI-3406 plus imatinib uniquely produced the deepest reduction in HSC/MPP- and LSC-like transcriptional modules.

The functional relevance of these findings is particularly important in CML, where disease persistence is largely attributed to primitive stem/progenitor populations that are incompletely eliminated by BCR::ABL1 kinase inhibition. Indeed, human CML stem cells can survive imatinib exposure despite effective inhibition of BCR::ABL1 kinase activity, supporting the notion that kinase blockade alone is insufficient to eradicate the disease-sustaining compartment [52]. Persistent BCR::ABL1⁺ stem cells have also been detected in patients after prolonged imatinib therapy, reinforcing the need for combination strategies capable of impairing self-renewal rather than only reducing bulk leukemic burden [53]. In this context, our serial MethoCult replating assays provided solid functional support for the transcriptomic reduction of stemness-associated programs observed after dual SOS1 and BCR::ABL1 inhibition. In both murine and human CML samples, the combination treatments reduced serial colony-forming capacity and depleted stem/progenitor-enriched compartments, including LSK cells in the p210^BCR/ABL^ model and Lin⁻CD34⁺CD38⁻ cells in patient-derived samples. The use of the murine LSK compartment is supported by prior BCR::ABL1-driven CML models showing that leukemia-propagating activity is enriched within primitive stem/progenitor populations [12, 54]. Importantly, combination-treated murine cultures failed to sustain colony formation upon secondary replating, and clinically resistant patient-derived samples lost secondary replating capacity after BI-3406+imatinib exposure. Thus, the transcriptional effects of the combination are accompanied by functional impairment of CML stem/progenitor self-renewal. These findings provide a strong functional basis for future in vivo limiting-dilution or secondary transplantation studies aimed at defining the long-term impact of dual SOS1 and BCR::ABL1 inhibition on leukemia-initiating activity.

Overall, our in vivo and in vitro findings highlight the markedly synergistic therapeutic benefit resulting from combining BI-3406 with classical TKI drugs, which also enhances TKI sensitivity in resistant CML contexts. We propose that the therapeutic benefit stems from the combined downmodulation of RAS/RAC downstream signaling by BI-3406 and specific CML signaling pathways by TKIs. Furthermore, based on our previous findings in KRAS-LUAD [9, 10], it is conceivable that dual SOS1 and BCR::ABL1 inhibition may also exert effects on the leukemic microenvironment beyond direct tumor-cell-intrinsic mechanisms; although this possibility was not directly assessed in the current study and represents an important direction for future investigation. Together, these observations are consistent with our prior report showing that CML cell lines exhibit the highest SOS1 dependency scores across all cancer types in the DepMap database [12] and suggest that further dissection of the transcriptional mechanisms by which SOS1 controls stemness in LSCs may reveal additional therapeutic vulnerabilities [55].

In summary, our preclinical data demonstrate that the SOS1 inhibitor BI-3406 synergizes with BCR::ABL1 TKIs across multiple complementary CML disease models, potentiating anti-leukemic activity, impairing CML stem/progenitor self-renewal, and sensitizing resistant cells to imatinib and other TKIs. These findings provide a compelling mechanistic and functional rationale for evaluating combined SOS1 and BCR::ABL1 inhibition in clinical studies, with the goals of deepening molecular responses, suppressing minimal residual disease, and overcoming clinically relevant forms of TKI resistance.

## Supplementary information

Supplementary information

Supplementary Table S4

Supplementary Table S5

Uncropped Western blot from Figure 6

## Data Availability

RNA-seq data for CML mice and patient samples are deposited in the Gene Expression Omnibus (GEO) database with accession number GSE303378.

## References

[1] Sicuranza A, Cavalleri A, Bernardi S. The biology of chronic myeloid leukemia: an overview of the new insights and biomarkers. Front Oncol. 2025;15:1546813. 10.3389/fonc.2025.1546813 PMC12095019 40406262

[2] Baccarani M, Cortés JE, Pane F, Niederwieser D, Saglio G, Apperley JF, et al. Chronic myeloid leukemia: an update of concepts and management recommendations of European LeukemiaNet. J Clin Oncol. 2009. 10.1200/jco.2009.25.0779. PMC4979100 19884523

[3] Saglio G, Gale RP. Prospects for achieving treatment-free remission in chronic myeloid leukaemia. Br J Haematol. 2020. 10.1111/bjh.16506. PMC7496116 32057102

[4] Hochhaus A, Baccarani M, Silver RT, Schiffer C, Apperley JF, Cervantes F, et al. European LeukemiaNet 2020 recommendations for treating chronic myeloid leukemia. Leukemia. 2020;34:966–84. 10.1038/s41375-020-0776-2 PMC7214240 32127639

[5] Mu H, Zhu X, Jia H, Zhou L, Liu H. Combination therapies in chronic myeloid leukemia for potential treatment-free remission: focus on leukemia stem cells and immune modulation. Front Oncol. 2021. 10.3389/fonc.2021.643382. PMC8155539 34055612

[6] Jain P, Kantarjian H, Boddu PC, Nogueras-González GM, Verstovsek S, Garcia-Manero G, et al. Analysis of cardiovascular and arteriothrombotic adverse events in chronic-phase CML patients after frontline TKIs. Blood Adv. 2019;3:851–61. 10.1182/bloodadvances.2018025874 PMC6436011 30885996

[7] Iurlo A, Cattaneo D, Bucelli C, Spallarossa P, Passamonti F. Cardiovascular Adverse Events of Tyrosine Kinase Inhibitors in Chronic Myeloid Leukemia: Clinical Relevance, Impact on Outcome, Preventive Measures and Treatment Strategies. Curr Treat Options Oncol. 2023;24:1720–38. 10.1007/s11864-023-01149-1 38047977

[8] Liceras-Boillos P, Jimeno D, García-Navas R, Lorenzo-Martín LF, Menacho-Marquez M, Segrelles C, et al. Differential role of the RasGEFs Sos1 and Sos2 in mouse skin homeostasis and carcinogenesis. Mol Cell Biol. 2018;38:e00049-18. 10.1128/MCB.00049-18 PMC6066753 29844066

[9] Baltanás FC, García-Navas R, Rodríguez-Ramos P, Calzada N, Cuesta C, Borrajo J, et al. Critical requirement of SOS1 for tumor development and microenvironment modulation in KRASG12D-driven lung adenocarcinoma. Nat Commun. 2023;14:5856. 10.1038/s41467-023-41583-1 PMC10511506 37730692

[10] Baltanás FC, Kramer-Drauberg M, García-Navas R, Patrucco E, Petrini E, Arnhof H, et al. SOS1 inhibitor BI-3406 shows in vivo antitumor activity akin to genetic ablation and synergizes with a KRASG12D inhibitor in KRAS LUAD. Proc Natl Acad Sci USA. 2025;122:e2422943122. 10.1073/pnas.2422943122 PMC11929440 40073053

[11] Gerboth S, Frittoli E, Palamidessi A, Baltanas FC, Salek M, Rappsilber J, et al. Phosphorylation of SOS1 on tyrosine 1196 promotes its RAC GEF activity and contributes to BCR-ABL leukemogenesis. Leukemia. 2018;32:820–7. 10.1038/leu.2017.267 PMC5739283 28819285

[12] Gómez C, Garcia-Navas R, Baltanás FC, Fuentes-Mateos R, Fernández-Medarde A, Calzada N, et al. Critical requirement of SOS1 for development of BCR/ABL-driven chronic myelogenous leukemia. Cancers. 2022;14:3893. 10.3390/cancers14163893 PMC9406065 36010887

[13] Liu Y, Li C, Su R, Yin Z, Huang G, Yang J, et al. Targeting SOS1 overcomes imatinib resistance with BCR-ABL independence through uptake transporter SLC22A4 in CML. Mol Ther Oncolytics. 2021;23:560–70. 10.1016/j.omto.2021.11.010 PMC8654699 34938856

[14] Luo Z, Lin C, Yu C, Yuan C, Wu W, Xu X, et al. Targeted degradation of SOS1 exhibits potent anticancer activity and overcomes resistance in KRAS-mutant tumors and BCR-ABL-positive leukemia. Cancer Res. 2025;85:101–17. 10.1158/0008-5472.CAN-24-1093 PMC11694061 39437162

[15] You X, Kong G, Ranheim EA, Yang D, Zhou Y, Zhang J. Unique dependence on Sos1 in KrasG12D-induced leukemogenesis. Blood. 2018;132:2575–9. 10.1182/blood-2018-09-874107 PMC6293870 30377195

[16] Begovich K, Schoolmeesters A, Rajapakse N, Martinez-Terroba E, Kumar M, Shakya A, et al. Cereblon-based bifunctional degrader of SOS1, BTX-6654, targets multiple KRAS mutations and inhibits tumor growth. Mol Cancer Ther. 2024. 10.1158/1535-7163.MCT-23-0513. 38224565

[17] Honda H, Oda H, Suzuki T, Takahashi T, Witte ON, Ozawa K, et al. Development of acute lymphoblastic leukemia and myeloproliferative disorder in transgenic mice expressing p210bcr/abl: a novel transgenic model for human Ph1-positive leukemias. Blood. 1998;91:2067–75. 9490692

[18] Qian X, Esteban L, Vass WC, Upadhyaya C, Papageorge AG, Yienger K, et al. The Sos1 and Sos2 Ras-specific exchange factors: differences in placental expression and signaling properties. EMBO J. 2000;19:642–54. 10.1093/emboj/19.4.642 PMC305602 10675333

[19] Baltanás FC, Pérez-Andrés M, Ginel-Picardo A, Diaz D, Jimeno D, Liceras-Boillos P, et al. Functional redundancy of Sos1 and Sos2 for lymphopoiesis and organismal homeostasis and survival. Mol Cell Biol. 2013;33:4562–78. 10.1128/MCB.01026-13 PMC3838185 24043312

[20] Tanaka Y, Fukushima T, Mikami K, Adachi K, Fukuyama T, Goyama S, et al. Efficacy of tyrosine kinase inhibitors on a mouse chronic myeloid leukemia model and chronic myeloid leukemia stem cells. Exp Hematol. 2020;90:46–51.e2. 10.1016/j.exphem.2020.09.186 32910995

[21] Cossarizza A, Chang H-D, Radbruch A, Abrignani S, Addo R, Akdis M, et al. Guidelines for the use of flow cytometry and cell sorting in immunological studies (third edition). Eur J Immunol. 2021;51:2708–3145. 10.1002/eji.202170126 PMC11115438 34910301

[22] Ianevski A, Giri AK, Aittokallio T. SynergyFinder 3.0: an interactive analysis and consensus interpretation of multi-drug synergies across multiple samples. Nucleic Acids Res. 2022. 10.1093/nar/gkac382. PMC9252834 35580060

[23] Liceras-Boillos P, García-Navas R, Ginel-Picardo A, Anta B, Pérez-Andrés M, Lillo C, et al. Sos1 disruption impairs cellular proliferation and viability through an increase in mitochondrial oxidative stress in primary MEFs. Oncogene. 2016;35:6389–402. 10.1038/onc.2016.169 27157612

[24] Bührer ED, Amrein MA, Forster S, Isringhausen S, Schürch CM, Bhate SS, et al. Splenic red pulp macrophages provide a niche for CML stem cells and induce therapy resistance. Leukemia. 2022;36:2634–46. 10.1038/s41375-022-01682-2 PMC7613762 36163264

[25] Jabbour E, Kantarjian H. Chronic myeloid leukemia: 2025 update on diagnosis, therapy, and monitoring. Am J Hematol. 2024;99:2191–212. 10.1002/ajh.27443 39093014

[26] Honda H, Hirai H. Model mice for BCR/ABL-positive leukemias. Blood Cells Mol Dis. 2001;27:265–78. 10.1006/bcmd.2000.0374 11358387

[27] Schemionek M, Spieker T, Kerstiens L, Elling C, Essers M, Trumpp A, et al. Leukemic spleen cells are more potent than bone marrow-derived cells in a transgenic mouse model of CML. Leukemia. 2011;26:1030–7. 10.1038/leu.2011.366 22193968

[28] Braun TP, Eide CA, Druker BJ. Response and resistance to BCR-ABL1-targeted therapies. Cancer Cell. 2020;37:530–42. 10.1016/j.ccell.2020.03.006 PMC7722523 32289275

[29] Thomas EK, Cancelas JA, Zheng Y, Williams DA. Rac GTPases as key regulators of p210-BCR-ABL-dependent leukemogenesis. Leukemia. 2008;22:898–904. 10.1038/leu.2008.71 PMC4464749 18354486

[30] Flis S, Bratek E, Chojnacki T, Piskorek M, Skorski T. Simultaneous inhibition of BCR-ABL1 tyrosine kinase and PAK1/2 serine/threonine kinase exerts synergistic effect against chronic myeloid leukemia cells. Cancers. 2019;11:1544. 10.3390/cancers11101544 PMC6826736 31614827

[31] Pochini L, Barone F, Console L, Brunocilla C, Galluccio M, Scalise M, et al. OCTN1 (SLC22A4) displays two different transport pathways for organic cations or zwitterions. Biochim Biophys Acta Biomembr. 2024;1866:184263. 10.1016/j.bbamem.2023.184263 38092232

[32] Jaruskova M, Curik N, Hercog R, Polivkova V, Motlova E, Benes V, et al. Genotypes of SLC22A4 and SLC22A5 regulatory loci are predictive of the response of chronic myeloid leukemia patients to imatinib treatment. J Exp Clin Cancer Res. 2017;36:55. 10.1186/s13046-017-0523-3 PMC5395939 28420426

[33] Malta TM, Sokolov A, Gentles AJ, Burzykowski T, Poisson L, Weinstein JN, et al. Machine learning identifies stemness features associated with oncogenic dedifferentiation. Cell. 2018;173:338–54.e15. 10.1016/j.cell.2018.03.034 PMC5902191 29625051

[34] Ng SWK, Mitchell A, Kennedy JA, Chen WC, McLeod J, Ibrahimova N, et al. A 17-gene stemness score for rapid determination of risk in acute leukaemia. Nature. 2016;540:433–7. 10.1038/nature20598 27926740

[35] Gullaksen S-E, Omsland M, Brevik M, Letzner J, Haugstvedt S, Gjertsen BT. CML stem cells and their interactions and adaptations to tyrosine kinase inhibitors. Leuk lymphoma. 2025;66:1211–20. 10.1080/10428194.2025.2466817 40598814

[36] Herrmann H, Sadovnik I, Eisenwort G, Rülicke T, Blatt K, Herndlhofer S, et al. Delineation of target expression profiles in CD34 + /CD38− and CD34 + /CD38+ stem and progenitor cells in AML and CML. Blood Adv. 2020;4:5118–32. 10.1182/bloodadvances.2020001742 PMC7594398 33085758

[37] Grassi S, Palumbo S, Mariotti V, Liberati D, Guerrini F, Ciabatti E, et al. The WNT pathway is relevant for the BCR-ABL1-independent resistance in chronic myeloid leukemia. Front Oncol. 2019. 10.3389/fonc.2019.00532. PMC6601352 31293972

[38] Long B, Zhu H, Zhu C, Liu T, Meng W. Activation of the hedgehog pathway in chronic myelogeneous leukemia patients. J Exp Clin Cancer Res. 2011;30:8. 10.1186/1756-9966-30-8 PMC3032744 21235817

[39] Aljedai A, Buckle A-M, Hiwarkar P, Syed F. Potential role of Notch signalling in CD34+ chronic myeloid leukaemia cells: cross-talk between Notch and BCR-ABL. PLoS ONE. 2015;10:e0123016. 10.1371/journal.pone.0123016 PMC4388554 25849484

[40] Nestorowa S, Hamey FK, Pijuan Sala B, Diamanti E, Shepherd M, Laurenti E, et al. A single-cell resolution map of mouse hematopoietic stem and progenitor cell differentiation. Blood. 2016;128:e20–31. 10.1182/blood-2016-05-716480 PMC5305050 27365425

[41] Zeng AGX, Iacobucci I, Shah S, Mitchell A, Wong G, Bansal S, et al. Single-cell transcriptional atlas of human hematopoiesis reveals genetic and hierarchy-based determinants of aberrant AML differentiation. Blood Cancer Discov. 2025;6:307–24. 10.1158/2643-3230.BCD-24-0342 PMC12209776 40294241

[42] Bermudez O, Marchetti S, Pagès G, Gimond C. Post-translational regulation of the ERK phosphatase DUSP6/MKP3 by the mTOR pathway. Oncogene. 2008;27:3685–91. 10.1038/sj.onc.1211040 18223677

[43] Hofmann MH, Gmachl M, Ramharter J, Savarese F, Gerlach D, Marszalek JR, et al. BI-3406, a potent and selective SOS1-KRAS interaction inhibitor, is effective in KRAS-driven cancers through combined MEK inhibition. Cancer Discov. 2021;11:142–57. 10.1158/2159-8290.CD-20-0142 PMC7892644 32816843

[44] Asada N. Regulation of malignant hematopoiesis by bone marrow microenvironment. Front Oncol. 2018. 10.3389/fonc.2018.00119. PMC5924781 29740536

[45] Ito K, Ito K. Leukemia stem cells as a potential target to achieve therapy-free remission in chronic myeloid leukemia. Cancers. 2021;13:5822. 10.3390/cancers13225822 PMC8616035 34830976

[46] Sini P, Cannas A, Koleske AJ, Di Fiore PP, Scita G. Abl-dependent tyrosine phosphorylation of Sos-1 mediates growth-factor-induced Rac activation. Nat Cell Biol. 2004;6:268–74. 10.1038/ncb1096 15039778

[47] Thatikonda V, Lyu H, Jurado S, Kostyrko K, Bristow CA, Albrecht C, et al. Co-targeting SOS1 enhances the antitumor effects of KRASG12C inhibitors by addressing intrinsic and acquired resistance. Nat Cancer. 2024;5:1352–70. 10.1038/s43018-024-00800-6 PMC11424490 39103541

[48] Higgins EM, Bos JM, Mason-Suares H, Tester DJ, Ackerman JP, MacRae CA, et al. Elucidation of *MRAS*-mediated Noonan syndrome with cardiac hypertrophy. JCI Insight. 2017. 10.1172/jci.insight.91225. PMC5333962 28289718

[49] Dudka W, Hoser G, Mondal SS, Turos-Korgul L, Swatler J, Kusio-Kobialka M, et al. Targeting integrated stress response with ISRIB combined with imatinib treatment attenuates RAS/RAF/MAPK and STAT5 signaling and eradicates chronic myeloid leukemia cells. BMC Cancer. 2022. 10.1186/s12885-022-10289-w. PMC9719211 36460969

[50] Pippa R, Odero MD. The role of MYC and PP2A in the initiation and progression of myeloid leukemias. Cells. 2020;9:544. 10.3390/cells9030544 PMC7140463 32110991

[51] Kong T, Laranjeira ABA, Yang K, Fisher DAC, Yu L, Poittevin De La Frégonnière L, et al. DUSP6 mediates resistance to JAK2 inhibition and drives leukemic progression. Nat Cancer. 2023;4:108–27. 10.1038/s43018-022-00486-8 PMC11288645 36581736

[52] Corbin AS, Agarwal A, Loriaux M, Cortes J, Deininger MW, Druker BJ. Human chronic myeloid leukemia stem cells are insensitive to imatinib despite inhibition of BCR-ABL activity. J Clin Invest. 2011;121:396–409. 10.1172/JCI35721 PMC3007128 21157039

[53] Chu S, McDonald T, Lin A, Chakraborty S, Huang Q, Snyder DS, et al. Persistence of leukemia stem cells in chronic myelogenous leukemia patients in prolonged remission with imatinib treatment. Blood. 2011;118:5565–72. 10.1182/blood-2010-12-327437 PMC3217358 21931114

[54] Schemionek M, Elling C, Steidl U, Bäumer N, Hamilton A, Spieker T, et al. BCR-ABL enhances differentiation of long-term repopulating hematopoietic stem cells. Blood. 2010;115:3185–95. 10.1182/blood-2009-04-215376 PMC2858483 20053753

[55] Tusa I, Cheloni G, Poteti M, Silvano A, Tubita A, Lombardi Z, et al. In Vitro Comparison of the effects of imatinib and ponatinib on chronic myeloid leukemia progenitor/stem cell features. Target Oncol. 2020;15:659–71. 10.1007/s11523-020-00741-x PMC7568716 32780298

