# Supplementary material for: Pharmacological SOS1 inhibitor BI-3406 synergizes with BCR::ABL1 TKIs reducing leukemia stem cells and overcoming imatinib resistance in CML
Source: Cell Death Dis. 2026 Aug 12;17(1):951. doi: 10.1038/s41419-026-09170-6 (PMC13748536; doi:10.1038/s41419-026-09170-6)
Supplement: Supplementary file 1 [file 41419_2026_9170_MOESM1_ESM.pdf]

Pharmacological SOS1 inhibitor BI-3406 synergizes with BCR::ABL1 TKIs reducing leukemia stem cells and overcoming imatinib resistance in CML

Rósula García-Navas, Carmela Gómez, Anabel Clemente-Serrano, Belén Zamora-Valdivieso, Sara Pérez-Gutiérrez, Andrea Olarte-San Juan, Alba Díaz-Aguilera, Sonsoles Calvo-Jimenez, Nuria Calzada, Alberto Fernandez-Medarde, Magdalena Sierra, Fermín Sánchez-Guijo, Dolors Colomer, Robyn L. Schenk, Marco H. Hofmann, Kaja Kostyrko, Eugenio Santos.

SUPPLEMENTARY INFORMATION

SUPPLEMENTARY METHODS

Reagents and Resources

All reagents, antibodies, small-molecule inhibitors, cell culture media, supplements, and oligonucleotides used in this study, including supplier information, catalog numbers, and working concentrations, are detailed in Supplementary Table S1.

Mouse Models and Treatment Protocol

Ethics and Housing

All mouse experiments were approved by the Bioethics Committee of the University of Salamanca (ID: #987) and conducted in the NUCLEUS animal facility in strict accordance with European (2007/526/CE) and Spanish (RD1201/2005; RD53/2013) regulations for animal care and experimentation. Animals were housed under specific-pathogen-free (SPF) barrier conditions, in ventilated cages with trunk wood bedding and environmental enrichment (nesting material and shelters). Housing conditions were maintained with 12:12-h light/dark cycles, 20-24 ºC ambient temperature, and 40-60% relative humidity. Autoclaved chow and water were provided *ad libitum*. Mice were group-housed unless required for clinical reasons. Male and female mice were used and balanced across treatment groups; no sex-specific differences were observed and data from both sexes were pooled.

Disease Onset and Hematology

Disease onset and enrollment were defined biologically, at 4-6 months of age. Mice were screened biweekly (every 15 days), and those with peripheral blood neutrophil

counts exceeding 35% were enrolled in the study. Peripheral blood samples (<100 μl) were collected from the submandibular vein into EDTA-coated tubes (Microvette® 500K3E, SARSTEDT). Hematologic parameters, including white blood cell counts, neutrophil percentages, and lymphocyte distributions, were measured using an Element HT5™ hematology analyzer. As pre-specified exclusion criteria, animals that developed severe anemia, rectal prolapse, or other unrelated health problems were humanely euthanized and excluded from further analysis. No mice or samples were excluded from the analysis beyond the pre-specified exclusion criteria described above.

Drug Formulation and Administration

Sample size was determined a priori using a power calculation with 80% power and α = 0.05, based on effect sizes observed in our previous work using the Tec-p210BCR/ABL CML model and related SOS1-targeting in vivo studies. These estimates were also considered together with the feasibility of generating age- and genotype-matched transgenic cohorts and ethical criteria aimed at minimizing animal use. Survival cohorts in the revised study were expanded to n = 7 mice per group to increase the robustness of the Kaplan–Meier analysis [1,2].

Mice received either BI-3406 (50 mg/kg, twice daily), imatinib (100 mg/kg, once daily), or the combination, administered by oral gavage for five consecutive weeks. BI-3406 was formulated at 5.45 mg/ml in 0.5% (w/v) hydroxyethylcellulose (Natrosol). For every 200 mg of BI-3406, an equimolar amount of 1 M HCl was added. The final solution was administered at 9.17 μl/g body weight[3]. Imatinib was prepared in sterile saline at 20 mg/ml and dosed at 5 μl/g[4]. BI-3406 and the Natrosol vehicle were supplied by Boehringer Ingelheim, while imatinib was purchased from MedChemExpress (MCE).

Investigators were not blinded during animal treatment administration. Animal monitoring and humane-endpoint decisions were based on predefined criteria applied uniformly across experimental groups. Flow-cytometry analyses were performed using standardized gating strategies, and histological/digital image quantifications were performed using predefined analysis workflows.

Efficacy Assessment and Humane Endpoints

Efficacy was assessed by survival, organ weights, histopathology, and blood/bone-marrow immunophenotyping at necropsy. Humane endpoints included rectal prolapse, a body-weight loss exceeding 20%, or a hemoglobin level below 6.0 g/dL.

Cell Lines and Patient-Derived Samples

Cell Line Culture and Mycoplasma Testing

The human cell lines K562 (ATCC® CCL-243™, RRID:CVCL_0004) and KCL22 (ATCC® CCL-3349™, RRID: CVCL_2091), were purchased from ATCC for this study and used with the corresponding certificate of analysis/authentication provided by the supplier. Murine Ba/F3-p210BCR/ABL cells, including the control strain and derivatives carrying the E255K or T315I mutations, were kindly provided by Dr Ángel Hernández-Hernández. The BCR::ABL1 p210 coding region was sequence-verified in our laboratory before experimental use, confirming the expected wild-type, E255K, or T315I sequence according to each Ba/F3-derived line. All cell lines were maintained in RPMI-1640 medium supplemented with 10% fetal bovine serum, 1% penicillin–streptomycin, and 2 mM L-glutamine at 37 ºC in a humidified atmosphere with 5% CO_2. Cells were routinely tested for mycoplasma contamination.

Primary Patient Samples

Primary bone marrow mononuclear cells (BMMNCs) were obtained from ten anonymized CML patients after written informed consent, in accordance with the Declaration of Helsinki and with protocols approved by the corresponding ethics committees: Complejo Asistencial Universitario de Salamanca CEIm (PI 2023101436) and Hospital Clínic de Barcelona CEIm (HCB/2022/1166). Mononuclear cells were isolated by Lymphoprep™ centrifugation. Subsequently, $CD34^+$ cells were isolated with EasySep™ Human CD34 Positive Selection Kit II (STEMCELL Technologies, #100-1569) following the manufacturer's protocol. Cells were counted and cultured in Iscove's Modified Dulbecco's Medium (IMDM) supplemented with a cocktail of human recombinant cytokines: 25 ng/ml Flt3L, 30 ng/ml SCF, 50 ng/ml IL-3, 10 ng/ml IL-6, and 30 ng/ml TPO [5,6].

Drug Sensitivity and Synergy Assays

Dose–response curves were generated using increasing concentrations of BI-3406 and the TKIs imatinib, dasatinib, nilotinib, and ponatinib. Combination treatments were performed using increasing TKI concentrations together with fixed concentrations of BI-3406. Apoptosis was assessed by Annexin V staining. IC50 values and synergy scores were calculated using the ZIP synergy model via the webtool[7]. Synergy was considered to occur with ZIP scores > 10.

Flow Cytometry and Clonogenic Assays

Bone Marrow Processing and Immunophenotyping

Bone marrow was collected from femurs and tibias by flushing with PBS + 2% fetal bovine serum, followed by ACK lysis and cell counting. Samples were stained with fluorochrome-conjugated antibodies (Supplementary Table S1) and acquired on a BD Accuri™ C6 Plus flow cytometer or a BD FACSAria™ III cell sorter, as appropriate.

For hematopoietic stem/progenitor cell (HSPC) analysis, events were first gated as cells (FSC-A/SSC-A) and singlets (FSC-A vs FSC-H). Lineage-negative (Lin^-) cells were defined by the absence of Ly6G/C (Gr-1), CD5, CD19, CD11b, CD3e, CD45R/B220, and Ter119. Multipotent progenitors (LSK) were identified within the Lin^- gate as Sca-1^+/c-Kit^+ cells. For all other populations, events were gated as cells (FSC-A/SSC-A), singlets (FSC-A vs FSC-H), and $CD45^+$ leukocytes before applying population-specific markers. Gates were established using fluorescence-minus-one (FMO) controls, and compensation was performed using single-stained controls. At least 50,000 events were recorded per sample and analyzed using FlowJo v10 (RRID:SCR_008520) [8].

Clonogenic Assays

Bone marrow cells were harvested and Lin^- cells were first obtained by fluorescence-activated cell sorting (FACS) on a BD FACSAria. For lineage exclusion, cells were stained with antibodies against CD5, CD11b, CD19, CD45R/B220, Ly6G/C (Gr-1), and TER119, and lineage-negative (Lin^-; marker-negative) cells were collected. Sorted cells were plated in MethoCult™ M3434 Classic methylcellulose medium and cultured for 10-14 days at 37ºC in 5% CO_2. Colonies were counted manually under an inverted microscope.

Histological Examination

Tissues were dissected, fixed for 24h with 4% paraformaldehyde, and washed in phosphate buffer. The processing and staining of the sections were performed by the PMC-BEOCyL Unit (Comparative Molecular Pathology-Biobank Network of Oncological Diseases of Castilla y León).

Pulldown Assays and Western Immunoblotting

Ras/Rac Activation Pulldown Assays

Pull-down activation assays were performed in K562 and KCL22 human CML cell lines following previously described protocols for the quantitation of RAS-GTP[3] and MRAS-GTP[9] and RAC-GTP[10] with specific modifications. Briefly, cells ($5x10^6$) were serum-starved for 12 hours and then treated with BI-3406 (1 µM), Imatinib (500 nM), or the combination (1 µM + 500 nM) for 2 hours. DMSO-treated cells (0.1% v/v) served as controls. After treatment, cells were stimulated with EGF (100 ng/mL) for

2, 5 and 10 minutes and lysed in ice-cold Mg^{2+} Lysis Buffer (MLB). Lysates (0.5 mL) were incubated for 1 hour at 4°C with 10 µg of bacterially expressed GST-Raf-1 RBD (RAS-GTP and MRAS-GTP) or GST-Pak-1 RBD (RAC-GTP) fusion proteins bound to Glutathione-Sepharose™ 4 Fast Flow beads (see Supplementary Table S1). After washing, protein complexes were eluted in SDS sample buffer and analyzed by SDS-PAGE followed by Western blot using specific primary antibodies (Supplementary Table S1). Total RAS, MRAS and RAC1 levels were used for normalization. Membranes were imaged using the Li-Cor Odyssey Imaging System (v3.0) and quantified with ImageJ (v1.54p, RRID:SCR_003070).

General Western Immunoblotting

Cells were lysed in Cell Lysis Buffer (1X) supplemented with 1 mM PMSF and cOmplete™ Protease Inhibitor Cocktail. Equal amounts of protein were resolved by SDS-PAGE, transferred to PVDF membranes, and probed with primary antibodies (Supplementary Table S1). Membranes were imaged using the Li-Cor Odyssey Imaging System v3.0 and quantified with ImageJ (v1.54p, RRID:SCR_003070).

RNA-seq Analyses

RNA Isolation and Sample Processing

Total RNA was isolated using the RNeasy Plus Mini Kit (Qiagen), and RNA integrity was assessed using an Agilent 2100 Bioanalyzer. For the murine RNA-seq analysis, RNA was obtained from bone marrow samples from all treatment groups. For the human RNA-seq analysis, BMMNCs from newly diagnosed CML patients were treated ex vivo with DMSO, imatinib (500 nM), BI-3406 (1 µM), or their combination. Each patient under each treatment condition was considered an independent biological replicate.

Sequencing and Bioinformatic Analysis

The RNA samples were sent to CeGaT GmbH (Tübingen, Germany) for library preparation, sequencing, and bioinformatic analysis. Libraries were generated using the TruSeq Stranded mRNA Library Prep Kit (Illumina) and sequenced on an Illumina NovaSeq 6000 platform to obtain 150-bp paired-end reads. Sequencing reads were aligned to the mouse (GRCm38/mm10) or human (GRCh38/hg38) reference genome using the STAR aligner v2.7.3 (RRID:SCR_004463) [11]. Differential gene expression analysis was performed using DESeq2 (RRID:SCR_000154) (FDR < 0.05)[12].

Functional Enrichment

Functional enrichment analyses of differentially expressed genes (DEGs; FDR < 0.05; |log2FC| ≥ 1) were performed using Enrichr (RRID:SCR_001575)[13]. Three

categories of libraries were interrogated: Reactome Pathways 2024, The Kinase Library 2024, and multiple transcription factor databases (Transcription_Factor_PPIs, TRANSFAC_and_JASPAR_PWMs, TRRUST_Transcription_Factors_2019, and ChEA_2022). The enrichment results are summarized in Supplementary Tables S4 (mouse) and S5 (human). Each table is organized into separate worksheets according to: (i) treatment condition (Imatinib, BI-3406, or Imatinib+BI-3406), (ii) direction of regulation (Upregulated or Downregulated genes), and (iii) enrichment library (Reactome, Kinase, or Transcription Factors). For transcription factors, results from the different libraries were combined into a single worksheet per treatment and regulation direction (e.g., Up_Mouse_IMA_TF), to facilitate comparison. Each worksheet includes the enriched term, gene overlap, p-value, adjusted p-value, odds ratio, combined score, and associated gene list. PCA plots, hierarchical clustering heatmaps, Venn diagrams, and dot plots were generated using R v4.0.4 within RStudio v2025.05.1+513.

Stemness Score Calculation

Gene expression data, provided as normalized count matrices for human and murine samples, along with corresponding metadata detailing treatment conditions, were imported and processed using the R statistical environment and packages from the tidyverse suite. The analysis environment was RStudio Desktop. The Stemness Score was calculated to quantify the relative activation of established self-renewal and immaturity gene programs within each sample, serving as a quantitative proxy for Leukemic Stem Cell (LSC) characteristics[14]. The scoring methodology adapts a standard gene set enrichment approach[15]. The score was derived from three pre-defined modules of genes that reflect critical biological programs for LSC identity:

First, a transcription factor (TF-stemness) module (MEIS1, HOXA9/10, BMI1, GATA2, HLF, TAL1, RUNX1, MYB, MECOM/EVI1, PROCR)[16] captured core regulators of hematopoietic and leukemic stem cell self-renewal. Second, a generic HSC/LSC surface marker module (CD34, KIT, THY1, PROM1/CD133, CXCR4, ITGA6, ITGB3, CD44, DPP4/CD26, IL1RAP, CD25/IL2RA, CD93)[17], as these markers have been shown to be aberrantly expressed on CML LSCs, but not on normal HSCs, and to correlate with disease burden and TKI response. Finally, a pathway-stemness module comprised Wnt/β-catenin (CTNNB1, LEF1, TCF7, AXIN2, MYC)[18], Hedgehog (GLI1, PTCH1)[19] and Notch (NOTCH1/2, HES1, HEY1)[20] targets, which are critical for HSC maintenance and leukemia stem cell self-renewal and are activated in myeloid leukemias including CML.

In addition, to place our human bulk RNA-seq data within a more granular hematopoietic differentiation framework, we analysed the patient BMMNC dataset using the 55 transcriptional cell-state signatures defined in the BoneMarrowMap single-cell reference atlas of human bone marrow hematopoiesis[21]. Marker genes for each of the 55 cell states (e.g. HSC, MPP-MyLy, LMPP, early GMP, GMP-cycle, GMP-NEU) were downloaded and stored as a list of gene sets. Only modules with at least two genes present in our expression matrix were retained.

For both the three LSC modules and the 55 BoneMarrowMap cell-state modules, scores were computed using an analogous z-score–based gene-set scoring strategy adapted from standard single-sample enrichment approaches.

To normalize gene expression differences across the entire dataset, the expression of every gene was standardized by calculating the Z-score row-wise (gene-centric):

$$\mathrm{Z}_{i,j} = \frac{X_{i,j} - \mu_i}{\sigma_i}$$

Where $X_{i,j}$ is the normalized expression value of gene i in sample j, μ_i is the mean expression of gene i across all samples in the dataset, and σ_i is the standard deviation of gene i across all samples.

The final Stemness Score for sample j within a specific module was calculated as the arithmetic mean of the Z-scores for all genes belonging to that module:

$$\text{Stemness Score}_j = \frac{1}{N_G} \sum_{i \in \text{Module}} Z_{i,j}$$

Where N_G is the number of module genes available in the expression matrix. A positive score indicates that the module's gene program is generally upregulated relative to the dataset mean.

Quantitative RT–qPCR

Quantitative RT–qPCR assays were performed using the Luna® Universal One-Step RT-qPCR Kit (see Supplementary Table S1 for full reagent details) according to the manufacturer's instructions. Reactions were run on a QuantStudio™ 5 Real-Time PCR System with 384-well block format (Thermo Fisher Scientific). Gene-specific primers are listed in Supplementary Table S1. Relative mRNA levels were calculated using the ΔΔCt method after normalization to 18S rRNA or beta2-microglobulin (B2m).

RNA-seq data availability

Raw and processed RNA-seq data are deposited in the Gene Expression Omnibus (GEO, RRID:SCR_005012) under the accession number GSE303378 (available upon request until public release). The dataset comprises transcriptomic profiles from bone marrow cells of Tec-p210BCR/ABL transgenic CML mice, including mice with conditional Sos1 deletion, and from BMMNCs isolated from newly diagnosed, untreated CML patients.

Statistical analysis

Statistical analyses were performed using GraphPad Prism v8.0 (RRID:SCR_002798). Data are presented as mean ± SEM from a minimum of three biological replicates. One-way ANOVA followed by Tukey's post hoc test was used for comparisons among multiple groups. Survival curves were analyzed using the Kaplan–Meier method and compared using the log-rank test. For drug combination studies, synergy scores were calculated using the ZIP model[7], and synergy was considered to occur with scores >10. A p-value < 0.05 was considered statistically significant.

REFERENCES

1 Gómez C, Garcia-Navas R, Baltanás FC, Fuentes-Mateos R, Fernández-Medarde A, Calzada N *et al.* Critical Requirement of SOS1 for Development of BCR/ABL-Driven Chronic Myelogenous Leukemia. *Cancers 2022, Vol 14, Page 3893* 2022; **14**: 3893.

2 Baltanás FC, García-Navas R, Rodríguez-Ramos P, Calzada N, Cuesta C, Borrajo J *et al.* Critical requirement of SOS1 for tumor development and microenvironment modulation in KRASG12D-driven lung adenocarcinoma. *Nat Commun* 2023; **14**: 5856.

3 Baltanás FC, Kramer-Drauberg M, García-Navas R, Patrucco E, Petrini E, Arnhof H *et al.* SOS1 inhibitor BI-3406 shows in vivo antitumor activity akin to genetic ablation and synergizes with a KRASG12D inhibitor in KRAS LUAD. *Proceedings of the National Academy of Sciences* 2025; **122**: e2422943122.

4 Tanaka Y, Fukushima T, Mikami K, Adachi K, Fukuyama T, Goyama S *et al.* Efficacy of tyrosine kinase inhibitors on a mouse chronic myeloid leukemia model and chronic myeloid leukemia stem cells. *Experimental Hematology* 2020; **90**: 46-51.e2.

5 Rondeau V, Espéli M, Balabanian K. Feeder-Free Differentiation Assay for Mouse Hematopoietic Stem and Progenitor Cells. *Methods Mol Biol* 2021; **2308**: 47–58.

6 Wilkinson AC, Igarashi KJ, Nakauchi H. Haematopoietic stem cell self-renewal in vivo and ex vivo. *Nat Rev Genet* 2020; **21**: 541–554.

7 Ianevski A, Giri AK, Aittokallio T. SynergyFinder 3.0: an interactive analysis and consensus interpretation of multi-drug synergies across multiple samples. *Nucleic Acids Research* 2022; **50**. doi:10.1093/nar/gkac382.

8 Cossarizza A, Chang H-D, Radbruch A, Abrignani S, Addo R, Akdis M *et al.* Guidelines for the use of flow cytometry and cell sorting in immunological studies (third edition). *European Journal of Immunology* 2021; **51**: 2708–3145.

9 Higgins EM, Bos JM, Mason-Suares H, Tester DJ, Ackerman JP, MacRae CA *et al.* Elucidation of *MRAS*-mediated Noonan syndrome with cardiac hypertrophy. *JCI Insight* 2017; **2**. doi:10.1172/jci.insight.91225.

10 Castro-Castro A, Ojeda V, Barreira M, Sauzeau V, Navarro-Lérida I, Muriel O *et al.* Coronin 1A promotes a cytoskeletal-based feedback loop that facilitates Rac1 translocation and activation. *The EMBO Journal* 2011; **30**: 3913–3927.

11 Dobin A, Davis CA, Schlesinger F, Drenkow J, Zaleski C, Jha S *et al.* STAR: ultrafast universal RNA-seq aligner. *Bioinformatics* 2013; **29**: 15–21.

12 Love MI, Huber W, Anders S. Moderated estimation of fold change and dispersion for RNA-seq data with DESeq2. *Genome Biology* 2014; **15**: 550.

13 Xie Z, Bailey A, Kuleshov MV, Clarke DJB, Evangelista JE, Jenkins SL *et al.* Gene Set Knowledge Discovery with Enrichr. *Curr Protoc* 2021; **1**: e90.

14 Malta TM, Sokolov A, Gentles AJ, Burzykowski T, Poisson L, Weinstein JN *et al.* Machine Learning Identifies Stemness Features Associated with Oncogenic Dedifferentiation. *Cell* 2018; **173**: 338-354.e15.

15 Hänzelmann S, Castelo R, Guinney J. GSVA: gene set variation analysis for microarray and RNA-Seq data. *BMC Bioinformatics* 2013; **14**: 7.

16 Gullaksen S-E, Omsland M, Brevik M, Letzner J, Haugstvedt S, Gjertsen BT. CML stem cells and their interactions and adaptations to tyrosine kinase inhibitors. *Leukemia & lymphoma* 2025; **66**: 1211–1220.

17 Herrmann H, Sadovnik I, Eisenwort G, Rülicke T, Blatt K, Herndlhofer S *et al.* Delineation of target expression profiles in CD34+/CD38− and CD34+/CD38+ stem and progenitor cells in AML and CML. *Blood Advances* 2020; **4**: 5118–5132.

18 Grassi S, Palumbo S, Mariotti V, Liberati D, Guerrini F, Ciabatti E *et al.* The WNT Pathway Is Relevant for the BCR-ABL1-Independent Resistance in Chronic Myeloid Leukemia. *Frontiers in Oncology* 2019; **9**. doi:10.3389/fonc.2019.00532.

19 Long B, Zhu H, Zhu C, Liu T, Meng W. Activation of the hedgehog pathway in chronic myelogeneous leukemia patients. *J Exp Clin Cancer Res* 2011; **30**: 8.

20 Aljedai A, Buckle A-M, Hiwarkar P, Syed F. Potential Role of Notch Signalling in CD34+ Chronic Myeloid Leukaemia Cells: Cross-Talk between Notch and BCR-ABL. *PLOS ONE* 2015; **10**: e0123016.

21 Zeng AGX, Iacobucci I, Shah S, Mitchell A, Wong G, Bansal S *et al.* Single-cell Transcriptional Atlas of Human Hematopoiesis Reveals Genetic and Hierarchy-Based Determinants of Aberrant AML Differentiation. *Blood Cancer Discov* 2025; **6**: 307–324.

Supplementary Table S1. Reagents, antibodies, oligonucleotides, and other resources used in this study

Category	Reagent / Resource	Supplier	Catalog number	Additional details
**Cell lines**	K562	ATCC	CCL-243	Human CML cell line
	KCL22	ATCC	CRL-3349	Human CML cell line
	Ba/F3-p210 WT / T315I / E255K	—	—	Murine pro-B cell line expressing BCR-ABL1 variants
**Plasmid**	Raf-1 GST RBD 1-149	Addgene	13338	pGEX-2T bacterial expression vector encoding GST followed by N-terminal residues 2-149 of human Raf-1
	pGEX-PAK-CRIB			Expression plasmid for the c-Myc-tagged Cdc42/Rac interacting binding (CRIB) domain (amino acids 67–150) of rat α p21-activated kinase (PAK) fused to GST was kindly provided by Xose Bustelo
**Primary cells**	Bone marrow mononuclear cells from CML patients	Hospital Universitario de Salamanca	—	Obtained under CEIm: PI 2023 10 1436
**Media & supplements**	RPMI-1640	Gibco, Thermo Fisher Scientific	11875-093	With 10% FBS, 1% pen/strep, 2 mM L-glutamine
	IMDM	Gibco	12440-053	For primary BMMNCs cells
	Fetal bovine serum (FBS)	Gibco	10270-106	Heat-inactivated
	Flt3L (recombinant human)	MedChemExpress	HY-P70544	25 ng/mL
	SCF (recombinant human)	MedChemExpress	HY-P70757	30 ng/mL
	IL-3 (recombinant human)	MedChemExpress	HY-P7040A	50 ng/mL
	IL-6 (recombinant human)	MedChemExpress	HY-P7044	10 ng/mL
	TPO (recombinant human)	MedChemExpress	HY-P7115	30 ng/mL
	EGF from murine submaxillary gland	Sigma-Aldrich	E1257	100ng/mL
	Glutathione-SepharoseTM 4 Fast Flow beads	Cytiva	7513201	
	Cell Lysis Buffer (10X)	Cell Signaling Technology	9803	
	cOmplete™ Protease Inhibitor Cocktail	Roche	4693116001	
	ACK Lysing Buffer	Gibco	A1049201	
**Small-molecule inhibitors**	BI-3406	Boehringer Ingelheim (OpnMe)	—	SOS1 inhibitor
	Imatinib	MedChemExpress	HY-15463	BCR-ABL1 TKI
	Dasatinib	MedChemExpress	HY-1018	BCR-ABL1/SRC TKI
	Nilotinib	MedChemExpress	HY-10159	BCR-ABL1 TKI
	Ponatinib	MedChemExpress	HY-12047	BCR-ABL1 TKI
**Antibodies**	Annexin V-FITC	Immunostep	ANXVF-200T	Apoptosis detection
	pERK1/2 (Thr202/Tyr204)	Cell Signaling Technology	4370	WB, 1:1000; IHC: 1:200
	ERK1/2	Cell Signaling Technology	4695	WB, 1:1000
	pAKT (Ser473)	Cell Signaling Technology	4060	WB, 1:1000
	AKT	Cell Signaling Technology	9272	WB, 1:1000
	panRAS	Millipore	05-516	WB, 1:1000
	RAC1	BD Biosciences	610650	WB, 1:1000
	β-Tubulin	Sigma-Aldrich	T4026	WB: 1:5000
	SOS1	Proteintech	55041-1-AP	WB, 1:1000
	SOS2 (B-6)	Santa Cruz Biotechnology	sc-393667	WB, 1:100
	Vinculin	Proteintech	26520-1-AP	WB: 1:5000
	MRAS	Proteintech	14213-1-AP	WB, 1:2000
	APC Rat Anti-Mouse CD117	BD Pharmingen	553356	FC: 1:200
	PE-Ly-6A/E (Sca-1) (Rat Anti-Mouse)	BD Pharmingen	562059	FC: 1:200
	FITC-Anti-mouse-CD11b (M1/70)	Biolegend	101206	FC: 1:200
	FITC Rat Anti-Mouse CD5	BD Pharmingen	553785	FC: 1:200
	FITC Rat Anti-Mouse CD19	BD Pharmingen	561740	FC: 1:200
	FITC-CD3e	BD Pharmingen	553062	FC: 1:200
	FITC-Ly6G/6C (Gr-1) (RB6-8C5)	Biolegend	108406	FC: 1:200
	FITC-TER-119 AntiMouse	Invitrogen	11-5921-82	FC: 1:200
	AlexaFluor488-CD45R/B220	BD Pharmingen	557669	FC: 1:200
	PerCP-Cy5.5 Rat Anti-Mouse CD11b	BD Pharmingen	550993	FC: 1:200
	APC-CD3e (145-2C11)	Invitrogen	17-0031-82	FC: 1:200
	PE-CD45R/B220	BD Pharmingen	553089	FC: 1:200
	FITC-CD45.2 Antibody, anti-mouse	Miltenyi	130-102-458	FC: 1:200
	BCR-ABL1 Fwd	Integrated DNA Technologies	*Homo sapiens*	CCAGGTGTATGAGCTGCTAGAG
	BCR-ABL1 Rev	Integrated DNA Technologies	*Homo sapiens*	GTCAGAGGGATTCCACTGCCAA
	GATA2 Fwd	Integrated DNA Technologies	*Homo sapiens*	ACTGACGGAGAGCATGAAGAT
	GATA2 Rev	Integrated DNA Technologies	*Homo sapiens*	CCGGCACATAGGAGGGGTA

Category	Reagent / Resource	Supplier	Catalog number	Additional details
**Oligonucleotides (qPCR)**	GLUT1 Fwd	Integrated DNA Technologies	*Homo sapiens*	TTGCAGGCTTCTCCAACTGGAC
	GLUT1 Rev	Integrated DNA Technologies	*Homo sapiens*	CAGAACCAGGAGCACAGTGAAG
	HIF1A Fwd	Integrated DNA Technologies	*Homo sapiens*	TATGAGCCAGAAGAACTTTTAGGC
	HIF1A Rev	Integrated DNA Technologies	*Homo sapiens*	CACCTCTTTTGGCAAGCATCCTG
	STAT5A Fwd	Integrated DNA Technologies	*Homo sapiens*	ACCTGTGGTTGTCATCGTCC
	STAT5A Rev	Integrated DNA Technologies	*Homo sapiens*	TTTGTCAGGCACGGCAAATG
	DUSP6 Fwd	Integrated DNA Technologies	*Homo sapiens*	CTCGGATCACTGGAGCCAAAAC
	DUSP6 Rev	Integrated DNA Technologies	*Homo sapiens*	GTCACAGTGACTGAGCGGCTAA
	MYC Fwd	Integrated DNA Technologies	*Homo sapiens*	CCTGGTGCTCCATGAGGAGAC
	MYC Rev	Integrated DNA Technologies	*Homo sapiens*	CAGACTCTGACCTTTTGCCAGG
	SLC22A4 Fwd	Integrated DNA Technologies	*Homo sapiens*	TCTTGGCAAGTCAGTTCGTATTA
	SLC22A4 Rev	Integrated DNA Technologies	*Homo sapiens*	CGCCAGTCTCTGATGAAGTAAG
	18S Fwd	Integrated DNA Technologies	*Homo sapiens*	ACCCGTTGAACCCCATTCGTGA
	18S Rev	Integrated DNA Technologies	*Homo sapiens*	GCCTCACTAAACCATCCAATCGG
	c-kit Fwd	Integrated DNA Technologies	*Mus musculus*	CCTCCTGCCCTTTATCCTTTAG
	c-kit Rev	Integrated DNA Technologies	*Mus musculus*	GACCTCCAAACCAGCTTACTT
	Hoxa9 Fwd	Integrated DNA Technologies	*Mus musculus*	GGCTTTGATGGAGGAGGTATT
	Hoxa9 Rev	Integrated DNA Technologies	*Mus musculus*	AGCTAGCACATACAGCCAATAG
	Runx-1 Fwd	Integrated DNA Technologies	*Mus musculus*	GGACATTCGGTCTTAGGGATTT
	Runx-1 Rev	Integrated DNA Technologies	*Mus musculus*	CCTCAACATCTCATGCCTTCT
	Myb Fwd	Integrated DNA Technologies	*Mus musculus*	GACAAGGGAAGAGGATGAGAAG
	Myb Rev	Integrated DNA Technologies	*Mus musculus*	GTCCGGTTGGGCAGATAAT
	Sca1 Fwd	Integrated DNA Technologies	*Mus musculus*	CTCAGGAGGCAGCAGTTATT
	Sca1 Rev	Integrated DNA Technologies	*Mus musculus*	GTACCCAGGATCTCCATACTTTC
	B2M Fwd	Integrated DNA Technologies	*Mus musculus*	ATGGGAAGCCGAACATACTG
	B2M Rev	Integrated DNA Technologies	*Mus musculus*	CAGTCTCAGTGGGGGTGAAT
	Meis1 Fwd	Integrated DNA Technologies	*Mus musculus*	GACAGAACGGACGATCATTCA
	Meis1 Rev	Integrated DNA Technologies	*Mus musculus*	CCTCAGACCCAACTACCAAATC
**Kits**	RNeasy Plus Mini Kit	Qiagen	74134	RNA extraction
	MethoCult™ M3434 Classic	STEMCELL Technologies	# 03444	Methylcellulose-based medium with recombinant cytokines (including EPO) for mouse cells
	EasySep™ Human CD34 Positive Selection Kit II	STEMCELL Technologies	#100-1569	Immunomagnetic positive selection of human CD34+ cells
	Lymphoprep™	STEMCELL Technologies	# 18061	Density gradient medium for the isolation of mononuclear cells
	Luna® Universal One-Step RT-qPCR Kit	New England Biolabs	E3005	
	AlamarBlue™	Invitrogen	Y00-100	Cell Viability Reagents
	iBlot™ 2 PVDF Regular Stacks	Invitrogen	IB2401	
**Equipment**	BD FACSAria™ III Cell Sorter	BD Biosciences		
	Incucyte® SX5 Live-Cell Analysis System	Sartorius	—	Non-adherent cell by cell assays
	QuantStudio™ 5 Real-Time PCR System with 384-well block format	Thermo Fisher Scientific		
	Element HT5™ Hematology Analyzer	scil animal care company S.L.U.		Veterinary Hematology Analyzer
**Software**	RStudio 2025.05.1+513 "Mariposa Orchid" Release	RStudio, PBC		
	GraphPad Prism 8.0.1	Dotmatic		
	FlowJo 10.4			

Supplementary Table S2. Statistical analysis of Kaplan–Meier survival curves for $p210^{BCR/ABL}$ CML mouse cohorts shown in Figure 1c. Median survival (days), hazard ratio (HR) with 95% confidence interval (CI) versus the vehicle-treated p210BCR/ABL group, p-value from the log-rank (Mantel–Cox) test, and survival proportion at day 180 (% of mice alive at the end of the observation period) are shown. HR < 1 indicates a survival benefit relative to the control group.

Group	Median survival (days)	HR (95% CI) Treated vs. $p210^{BCR/ABL}$	p-value (Log-rank) vs vehicle	p-value (Log-rank) vs IMA	p-value (Log-rank) vs SOS1 KO	p-value (Log-rank) vs BI-3406	Survival proportion at day 180 (%)
$p210^{BCR/ABL}$	46	Ref.	—	—	—	—	0
$p210^{BCR/ABL}$ + IMA	78	0.3347 (0.05–0.97)	0.032	—	0.0293	0.0012	0
$p210^{BCR/ABL}$ SOS1KO	105	0.2481 (0.06–0.91)	0.022	0.0293	—	0.0012	0
$p210^{BCR/ABL}$ + BI-3406	49	1.005 (0.26–5.66)	0.849	0.0012	0.0012	—	0
$p210^{BCR/ABL}$ SOS1KO + IMA	>180	0.114 (0.02–0.48)	0.007	0.0008	0.03	0.0001	85.7
$p210^{BCR/ABL}$ + IMA + BI-3406	>180	0.06343 (0.01–0.28)	0.002	0.0004	0.0112	0.0002	85.7

Supplementary Table S3. Clinical characteristics of CML patient-derived bone marrow samples used in ex vivo assays. Demographic data, disease status, BCR::ABL1 kinase-domain mutation status, clinical grouping, treatment history, and sampling context of the CML patient-derived bone marrow mononuclear cells used in ex vivo assays.

Patient	Sex	Age	BCR::ABL1 mutation status	Clinical group used in this study	TKI Regimen	Clinical follow-up
1	M	70	None detected by NGS	Treatment-naïve at diagnosis, imatinib-sensitive	Imatinib 400 mg	Well tolerated to date
2	M	77	None detected by NGS	Treatment-naïve at diagnosis, imatinib-sensitive	Imatinib 400 mg	Subsequently switched to bosutinib due to hematologic toxicity and inadequate response
3	M	63	None detected by NGS	Treatment-naïve at diagnosis, imatinib-sensitive	Imatinib 400 mg	Mild conjunctivitis, no dose modification needed
4	M	72	None detected by NGS	Treatment-naïve at diagnosis, imatinib-sensitive	Imatinib 400 mg	Recurrent dermatologic toxicity; switched to dasatinib 100 mg
5	M	62	None detected by NGS	Treatment-naïve at diagnosis, imatinib-sensitive	Imatinib 400 mg	Gastrointestinal toxicity, dose reduction required
6	M	35	T315I	Imatinib-resistant	Ponatinib 45 mg	Ponatinib discontinued after 1 month due to thrombocytopenia; subsequently underwent sibling donor allo-HSCT
7	M	65	None detected by NGS	Imatinib-resistant	Bosutinib 500 mg	Switched to asciminib 80mg
8	M	74	None detected by NGS	Imatinib-resistant	Imatinib 400 mg	Switched to bosutinib 300 mg
9	M	59	None detected by NGS	Imatinib-resistant	Imatinib 400 mg	Switched to dasatinib 100 mg and subsequently to ponatinib 45 mg. Underwent unrelated donor allo-HSCT one year later; died from septic shock three months after transplantation.
10	M	56	None detected by NGS	Imatinib-resistant	Asciminib 40 mg+ Venetoclax + Decitabine	Accelerated phase at diagnosis; relapse sample used for ex vivo assays

Supplementary Table S4. Enrichment analysis of mouse bone marrow RNA-seq data. The complete dataset is provided as a separate Excel workbook. This Excel workbook compiles the results of Enrichr over-representation analyses performed on differentially expressed genes (DEGs) identified in bone marrow from $p210^{BCR/ABL}$ CML mice treated with BI-3406 (BI), imatinib (IMA), or their combination (COMBO), relative to vehicle-treated controls. DEG lists were generated as described in Methods (FDR < 0.05; $|\log_2 FC| \geq 1$ when indicated). Each worksheet corresponds to one treatment × regulation direction (up- or downregulated) × database category. The following Enrichr libraries were used: Reactome Pathways 2024 (Reactome); The Kinase Library 2024 (Kinase); Transcription factor databases: Transcription_Factor_PPIs, TRANSFAC and JASPAR_PWMs, TRRUST Transcription Factors 2019, and ChEA_2022 (all grouped under TF). Only significantly enriched terms (adjusted P < 0.05, Benjamini–Hochberg) are reported. For example, the worksheet Up_Mouse_IMA_Reactome contains Reactome pathways significantly enriched among genes upregulated by imatinib in mouse bone marrow. This dataset provides the source information for the enrichment results shown in Fig. 7c and Supplementary Fig. S6 (mouse panels).

Supplementary Table S5. Enrichment analysis of human bone marrow RNA-seq data. The complete dataset is provided as a separate Excel workbook. This Excel workbook compiles the results of Enrichr over-representation analyses performed on differentially expressed genes (DEGs) identified in ex vivo bone marrow samples from CML patients treated *ex vivo* with BI-3406 (BI), imatinib (IMA), or their combination (COMBO), relative to untreated controls. DEG lists were generated as described in Methods (FDR < 0.05; $|\log_2 FC| \geq 1$ when indicated). Each worksheet corresponds to one treatment × regulation direction (up- or downregulated) × database category. The following Enrichr libraries were used: Reactome Pathways 2024 (Reactome); The Kinase Library 2024 (Kinase); Transcription factor databases: Transcription_Factor_PPIs, TRANSFAC and JASPAR_PWMs, TRRUST Transcription Factors 2019, and ChEA 2022 (all grouped under TF). Only significantly enriched terms (adjusted P < 0.05, Benjamini–Hochberg) are reported. For example, the worksheet Up_Human_IMA_Reactome contains Reactome pathways significantly enriched among genes upregulated by imatinib in patient-derived bone marrow. This dataset provides the source information for the enrichment results shown in Fig. 7d and Supplementary Fig. S6 (human panels).

Supplementary Table S6. Estimated absolute bone marrow cell numbers in treated p210BCR/ABL CML mice. Individual mouse values used to estimate absolute numbers of major bone-marrow populations analyzed in Figure 2d–g are shown. Total bone-marrow cellularity was determined after bone-marrow recovery and estimated absolute cell numbers were calculated by multiplying total bone-marrow cells by the corresponding flow-cytometry frequency among singlet events. Populations include CD45R/B220^{+} B cells, CD3^{+} T cells, CD11b^{+}Gr1^{+} cells, and Lin^{-}Sca-1^{+}c-Kit^{+} LSK cells. Group-level mean ± SEM values are summarized in Figure 2i.

Mouse ID	Group	Total BM cells	B220^{+} (%)	Estimated B220^{+} cells	CD3^{+} (%)	Estimated CD3^{+} cells	CD11b^{+}Gr1^{+} (%)	Estimated CD11b^{+}Gr1^{+} cells	LSK (%)	Estimated LSK cells
EE210	non-p210BCR/ABL	1,99E+07	9,44	1,88E+06	2,04	4,06E+05	22,3	4,44E+06	2,23	4,44E+05
EE211	non-p210BCR/ABL	4,05E+07	11,06	4,48E+06	2,01	8,14E+05	16,5	6,68E+06	1,85	7,49E+05
EE170	non-p210BCR/ABL	4,37E+07	12	5,24E+06	1,46	6,38E+05	28	1,22E+07	2,03	8,87E+05
EE213	non-p210BCR/ABL	3,22E+07	13,3	4,28E+06	0,91	2,93E+05	23	7,41E+06	2,22	7,15E+05
EE322	non-p210BCR/ABL	3,38E+07	16,1	5,44E+06	1,18	3,99E+05	25,4	8,59E+06	2,62	8,86E+05
EE335	non-p210BCR/ABL	3,72E+07	13,9	5,17E+06	1,09	4,05E+05	20,4	7,59E+06	2,19	8,15E+05
EE557	non-p210BCR/ABL	4,92E+07	13,4	6,59E+06	1,28	6,30E+05	18,9	9,30E+06	2,89	1,42E+06
EE317	non-p210BCR/ABL	3,06E+07	15,7	4,80E+06	1,12	3,43E+05	17	5,20E+06	2,31	7,07E+05
EE333	non-p210BCR/ABL	3,11E+07	12,1	3,76E+06	1,01	3,14E+05	19	5,91E+06	3,09	9,61E+05
EE654	non-p210BCR/ABL	2,37E+07	15,49	3,67E+06	1,47	3,48E+05	21,5	5,10E+06	2,72	6,45E+05
EE660	non-p210BCR/ABL	1,14E+07	10,28	1,17E+06	2,25	2,57E+05	25,98	2,96E+06	2,91	3,32E+05
EE344	p210BCR/ABL	1,34E+08	2,53	3,39E+06	2,47	3,31E+06	68,2	9,14E+07	4,07	5,45E+06
EE154	p210BCR/ABL	6,47E+07	7,32	4,74E+06	2,03	1,31E+06	60,4	3,91E+07	5,15	3,33E+06
EE307	p210BCR/ABL	1,65E+08	3,61	5,96E+06	2,52	4,16E+06	59,1	9,75E+07	2,88	4,75E+06
MM235	p210BCR/ABL	1,63E+08	2,93	4,78E+06	2,2	3,59E+06	49,4	8,05E+07	3,69	6,01E+06
MM240	p210BCR/ABL	1,57E+08	7,34	1,15E+07	1,83	2,87E+06	71,2	1,12E+08	4,59	7,21E+06
MM236	p210BCR/ABL	6,92E+07	4,27	2,95E+06	1,82	1,26E+06	60,1	4,16E+07	3,49	2,42E+06
MM248	p210BCR/ABL	6,60E+07	4,22	2,79E+06	1,76	1,16E+06	45,8	3,02E+07	3,24	2,14E+06
EE730	p210BCR/ABL	1,31E+08	3,36	4,40E+06	2,11	2,76E+06	48,6	6,37E+07	4,15	5,44E+06
EE151	p210BCR/ABL	1,64E+08	6,45	1,06E+07	1,76	2,89E+06	68,5	1,12E+08	2,99	4,90E+06
MM254	p210BCR/ABL	1,29E+08	8,07	1,04E+07	1,35	1,74E+06	79,4	1,02E+08	3,66	4,72E+06
MM255	p210BCR/ABL	1,59E+08	7,42	1,18E+07	1,16	1,84E+06	65,9	1,05E+08	5,56	8,84E+06
EE156	p210BCR/ABL + Imatinib	6,18E+07	4,44	2,74E+06	1,67	1,03E+06	56,7	3,50E+07	4,03	2,49E+06
EE223	p210BCR/ABL + Imatinib	6,40E+07	7,15	4,58E+06	1,31	8,38E+05	36	2,30E+07	4,15	2,66E+06
R29	p210BCR/ABL + Imatinib	1,04E+08	5,13	5,34E+06	1,37	1,42E+06	51,9	5,40E+07	5,5	5,72E+06
EE306	p210BCR/ABL + Imatinib	6,76E+07	12,5	8,45E+06	1,45	9,80E+05	41,6	2,81E+07	3,53	2,39E+06
EE345	p210BCR/ABL + Imatinib	8,86E+07	4,11	3,64E+06	2,14	1,90E+06	21,4	1,90E+07	5,5	4,87E+06
MM243	p210BCR/ABL + Imatinib	1,04E+08	10,8	1,12E+07	1,13	1,18E+06	57,4	5,97E+07	3,17	3,30E+06
MM244	p210BCR/ABL + Imatinib	4,87E+07	10,4	5,06E+06	1,31	6,38E+05	23,2	1,13E+07	2,97	1,45E+06
MM247	p210BCR/ABL + Imatinib	1,11E+08	4,11	4,56E+06	1,56	1,73E+06	51,8	5,75E+07	3,64	4,04E+06
MM259	p210BCR/ABL + Imatinib	8,38E+07	10,8	9,05E+06	1,08	9,05E+05	57,4	4,81E+07	4,16	3,49E+06
EE168	p210BCR/ABL SOS1KO	6,73E+07	2,19	1,47E+06	1,33	8,95E+05	65,5	4,41E+07	2,96	1,99E+06
EE456	p210BCR/ABL SOS1KO	5,19E+07	4,69	2,43E+06	2,45	1,27E+06	37,2	1,93E+07	4,2	2,18E+06
EE477	p210BCR/ABL SOS1KO	3,47E+07	5,52	1,92E+06	1,61	5,59E+05	64,1	2,22E+07	1,39	4,82E+05
EE478	p210BCR/ABL SOS1KO	5,14E+07	10,1	5,19E+06	1,48	7,61E+05	51,8	2,66E+07	1,94	9,97E+05
EE592	p210BCR/ABL SOS1KO	9,37E+07	3,59	3,36E+06	1,44	1,35E+06	63,1	5,91E+07	1,38	1,29E+06
EE593	p210BCR/ABL SOS1KO	6,73E+07	5,05	3,40E+06	1,32	8,88E+05	24,8	1,67E+07	1,99	1,34E+06
EE717	p210BCR/ABL SOS1KO	3,43E+07	2,2	7,55E+05	1,33	4,56E+05	48,4	1,66E+07	1,14	3,91E+05
EE683	p210BCR/ABL SOS1KO	4,20E+07	3,85	1,62E+06	2,13	8,95E+05	37,6	1,58E+07	1,19	5,00E+05
MM225	p210BCR/ABL SOS1KO	9,31E+07	4,95	4,61E+06	2,25	2,09E+06	37,2	3,46E+07	1	9,31E+05
EE346	p210BCR/ABL + BI3406	4,74E+07	5,71	2,71E+06	1,83	8,67E+05	79,4	3,76E+07	2,24	1,06E+06
EE498	p210BCR/ABL + BI3406	1,14E+08	1,58	1,80E+06	1,17	1,33E+06	51,1	5,83E+07	2,71	3,09E+06
EE302	p210BCR/ABL + BI3406	6,48E+07	5,43	3,52E+06	1,01	6,54E+05	44,7	2,90E+07	4,69	3,04E+06
EE349	p210BCR/ABL + BI3406	6,46E+07	8,9	5,75E+06	1,53	9,88E+05	26,5	1,71E+07	5,31	3,43E+06
EE851	p210BCR/ABL + BI3406	1,05E+08	4,87	5,11E+06	1,54	1,62E+06	22,9	2,40E+07	2,13	2,24E+06
EE728	p210BCR/ABL + BI3406	4,88E+07	9,89	4,83E+06	1,86	9,08E+05	26,9	1,31E+07	1,22	5,95E+05
EE677	p210BCR/ABL + BI3406	6,95E+07	13,3	9,24E+06	1,28	8,90E+05	40,2	2,79E+07	2,46	1,71E+06
EE850	p210BCR/ABL + BI3406	6,50E+07	9	5,85E+06	1,34	8,71E+05	51,8	3,37E+07	5,2	3,38E+06
EE725	p210BCR/ABL + BI3406	6,69E+07	3,55	2,37E+06	1,12	7,49E+05	65,8	4,40E+07	2,59	1,73E+06
EE163	p210BCR/ABL SOS1KO+IMA	3,07E+07	6,74	2,07E+06	2,29	7,03E+05	68,5	2,10E+07	1,72	5,28E+05
EE353	p210BCR/ABL SOS1KO+IMA	5,18E+07	5,95	3,08E+06	1,39	7,20E+05	32,9	1,70E+07	2,24	1,16E+06
EE678	p210BCR/ABL SOS1KO+IMA	3,67E+07	6,54	2,40E+06	1,48	5,43E+05	11,7	4,29E+06	0,78	2,86E+05
EE720	p210BCR/ABL SOS1KO+IMA	3,42E+07	8,59	2,94E+06	0,82	2,80E+05	37	1,27E+07	1,13	3,86E+05
EE846	p210BCR/ABL SOS1KO+IMA	7,20E+07	1,76	1,27E+06	2,68	1,93E+06	60,8	4,38E+07	1,67	1,20E+06
EE718	p210BCR/ABL SOS1KO+IMA	6,29E+07	8,89	5,59E+06	1,72	1,08E+06	34	2,14E+07	0,92	5,79E+05
MM224	p210BCR/ABL SOS1KO+IMA	2,80E+07	5,88	1,65E+06	1,05	2,94E+05	37,4	1,05E+07	1,01	2,83E+05
EE719	p210BCR/ABL SOS1KO+IMA	5,77E+07	3,73	2,15E+06	1,63	9,41E+05	37	2,13E+07	3,81	2,20E+06
EE721	p210BCR/ABL SOS1KO+IMA	4,74E+07	5,95	2,82E+06	2,53	1,20E+06	30,7	1,46E+07	1,07	5,07E+05

Mouse ID	Group	Total BM cells	B220⁺ (%)	Estimated B220⁺ cells	CD3⁺ (%)	Estimated CD3⁺ cells	CD11b⁺Gr1⁺ (%)	Estimated CD11b⁺Gr1⁺ cells	LSK (%)	Estimated LSK cells
R28	p210BCR/ABL + BI3406+IMA	4,43E+07	2,59	1,15E+06	1,63	7,22E+05	51,3	2,27E+07	1,44	6,38E+05
EE347	p210BCR/ABL + BI3406+IMA	4,71E+07	4,45	2,10E+06	1,66	7,82E+05	39	1,84E+07	3,47	1,63E+06
EE343	p210BCR/ABL + BI3406+IMA	6,33E+07	4,28	2,71E+06	1,95	1,23E+06	42,7	2,70E+07	2,24	1,42E+06
EE342	p210BCR/ABL + BI3406+IMA	4,75E+07	6,1	2,90E+06	1,28	6,08E+05	23,2	1,10E+07	2,93	1,39E+06
EE499	p210BCR/ABL + BI3406+IMA	5,14E+07	6,52	3,35E+06	1,75	9,00E+05	21,1	1,08E+07	1,12	5,76E+05
EE731	p210BCR/ABL + BI3406+IMA	1,71E+07	9,2	1,57E+06	2,26	3,86E+05	20,8	3,56E+06	1,4	2,39E+05
EE852	p210BCR/ABL + BI3406+IMA	3,53E+07	7,2	2,54E+06	1,79	6,32E+05	19,2	6,78E+06	0,7	2,47E+05
EE854	p210BCR/ABL + BI3406+IMA	3,31E+07	6,35	2,10E+06	2,2	7,28E+05	13,1	4,34E+06	0,78	2,58E+05
EE681	p210BCR/ABL + BI3406+IMA	2,92E+07	8,89	2,60E+06	1,94	5,66E+05	48,7	1,42E+07	1,18	3,45E+05

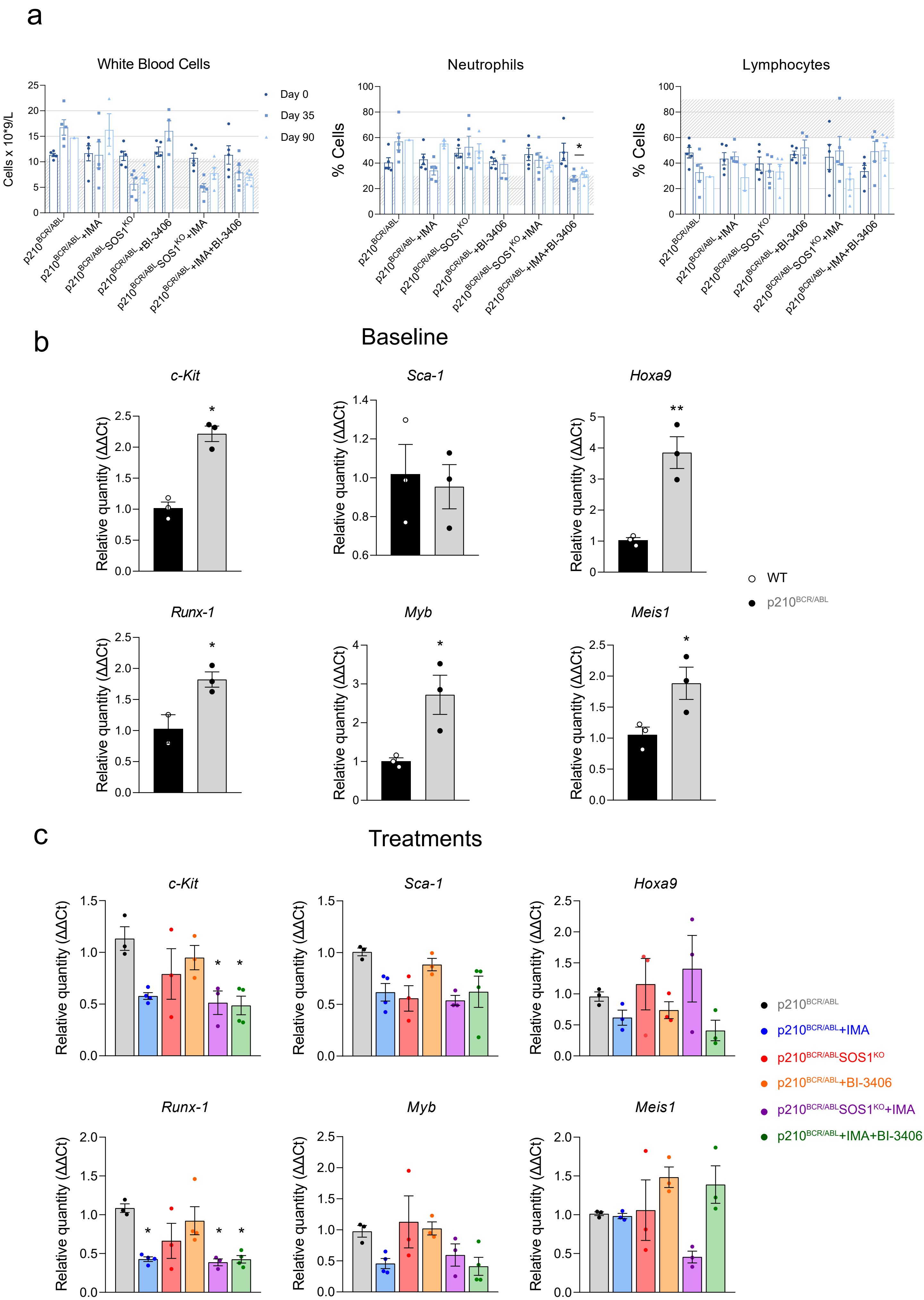

a
White Blood Cells
Cells x 10*9/L
Day 0
Day 35
Day 90
Neutrophils
% Cells
Lymphocytes
% Cells
p210BCR/ABL
p210BCR/ABL+IMA
p210BCR/ABLSOS1KO
p210BCR/ABL+BI-3406
p210BCR/ABLSOS1KO+IMA
p210BCR/ABL+IMA+BI-3406
b
Baseline
c-Kit
Sca-1
Hoxa9
Runx-1
Myb
Meis1
Relative quantity (ΔΔCt)
WT
p210BCR/ABL
c
Treatments
c-Kit
Sca-1
Hoxa9
Runx-1
Myb
Meis1
Relative quantity (ΔΔCt)
p210BCR/ABL
p210BCR/ABL+IMA
p210BCR/ABLSOS1KO
p210BCR/ABL+BI-3406
p210BCR/ABLSOS1KO+IMA
p210BCR/ABL+IMA+BI-3406

Supplementary Figure S1. Dual SOS1 and BCR::ABL1 inhibition sustains long-term hematological *in vivo* therapy responses and downregulates leukemic stemness-associated gene expression in p210$^{BCR/ABL}$ CML mice.

(a) Hematologic parameters were measured at baseline (Day 0), end of treatment (Day 35), and two months post-treatment withdrawal (Day 90). Total white blood cell (WBC) and neutrophil (NEU) counts decreased in all treatment arms at day 35; however, only dual inhibition sustained these reductions at day 90. Imatinib monotherapy was associated with hematologic relapses, while BI-3406 monotherapy was ineffective, with rapid disease progression. Lymphocyte (LYM) percentages remained below the normal reference range in most groups, except for a transient change observed in the SOS1^{KO} group. Together, these data demonstrate that dual targeting achieves superior control of disease burden and prevents relapse. Data represent mean ± SEM from $n \geq 4$ mice per group; *$p < 0.05$, vs. Day 0 (two-way ANOVA with Tukey's post-test). The shaded area represents normal reference ranges for mice.

(b) Baseline expression of stemness-associated genes in spleen from WT vs. leukemic p210$^{BCR/ABL}$ mice, highlighting their marked upregulation in the CML model (*Hoxa9, Kit, Runx1, Myb*) and providing the rationale for their use as molecular readouts in treatment studies. Data represent mean ± SEM (n = 3–4 per group). *$p < 0.05$, **$p<0.01$ by one-way ANOVA with Tukey's post-test.

(c) RT-qPCR analysis of spleen samples from p210$^{BCR/ABL}$ mice treated for 5 weeks with vehicle, BI-3406, imatinib (IMA), or the combination BI-3406+IMA. The combination treatment reduced the expression of *Hoxa9, Myb, c-Kit*, and *Runx1* compared to control or monotherapy groups, while *Sca-1* and *Meis1* remained largely unchanged. Gene expression was normalized to 18S rRNA and expressed as fold-change relative to WT or vehicle-treated controls. Data represent mean ± SEM (n = 3–4 per group). *$p < 0.05$ by one-way ANOVA with Tukey's post-test.

a

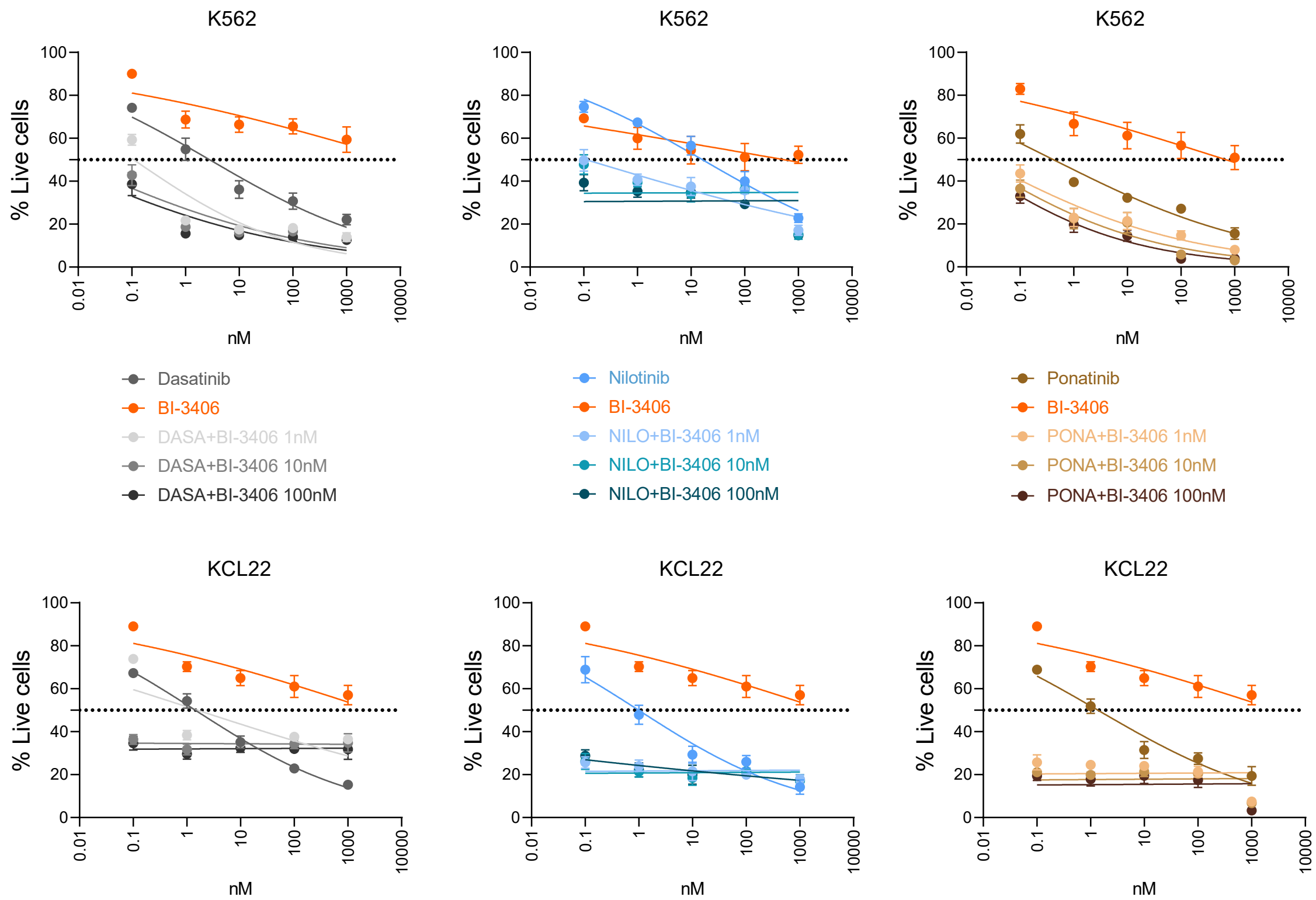

b

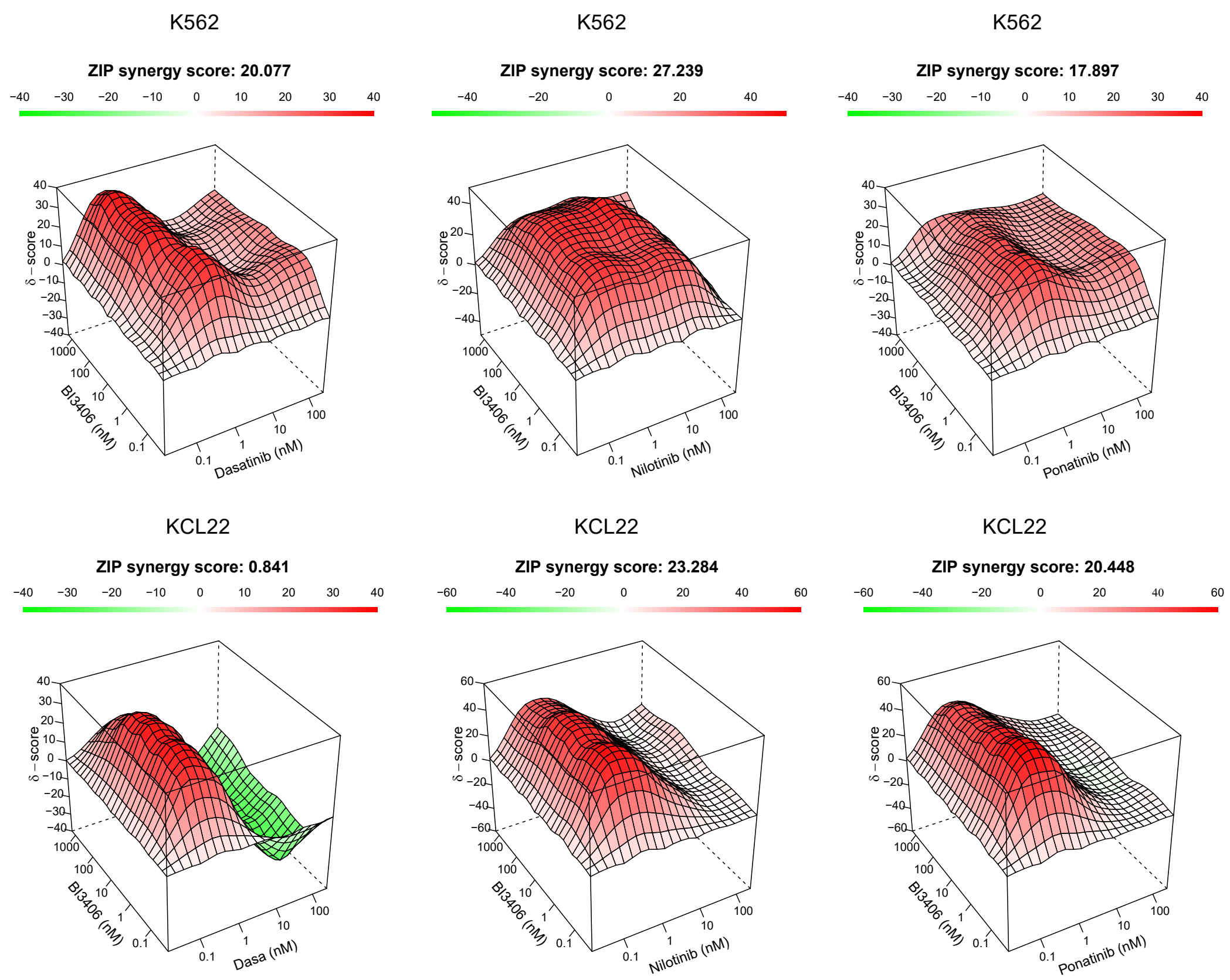

c

	IC50 (nM)	
	K562	KCL22
DASATINIB (nM)	2.8±0.9	1.4±0.2
DASA+BI-3406 1nM	0.108±0.05	1.5±1.3
DASA+BI-3406 10nM	<0.01	<0.01
DASA+BI-3406 100nM	<0.01	<0.01
NILOTINIB (nM)	16.3±4.4	0.9±0.3
NILO+BI-3406 1nM	0.111±0.1	<0.01
NILO+BI-3406 10nM	<0.01	<0.01
NILO+BI-3406 100nM	<0.01	<0.01
PONATINIB (nM)	0.4±0.1	1.3±0.4
PONA+BI-3406 1nM	0.017±0.01	<0.01
PONA+BI-3406 10nM	0.012±0.01	<0.01
PONA+BI-3406 100nM	<0.01	<0.01

Supplementary Figure S2. BI-3406 enhances sensitivity to second- and third-generation TKIs in K562 and KCL22 CML cell lines.

(a) Dose–response viability curves of K562 and KCL22 cells treated with dasatinib (left panels), nilotinib (middle panels), or ponatinib (right panels), alone or in combination with fixed doses of BI-3406 (1nM, 10nM, or 100nM). BI-3406 monotherapy had minimal effect on viability but markedly potentiated the cytotoxic activity of all three TKIs in both cell lines, resulting in a pronounced reduction in IC_{50} values (see table in panel c).

(b) Synergy analysis of BI-3406 in combination with dasatinib, nilotinib, or ponatinib using the ZIP model. 3D surface plots show areas of synergy (red) across concentration matrices. ZIP synergy scores >10 denote strong combinatorial interaction. Synergistic effects were observed for most combinations, with particularly high scores for nilotinib combinations. Although dasatinib plus BI-3406 showed a low overall ZIP synergy score in KCL22 cells, localized synergistic activity was detected within specific concentration ranges, as reflected by a most synergistic area score above 20. These data indicate that BI-3406 enhances the activity of second- and third-generation TKIs, although the magnitude and distribution of synergy varied according to the TKI and cell line.

Data presented as mean ± SEM from three independent replicates. IC_{50} values were calculated after 48 h treatment.

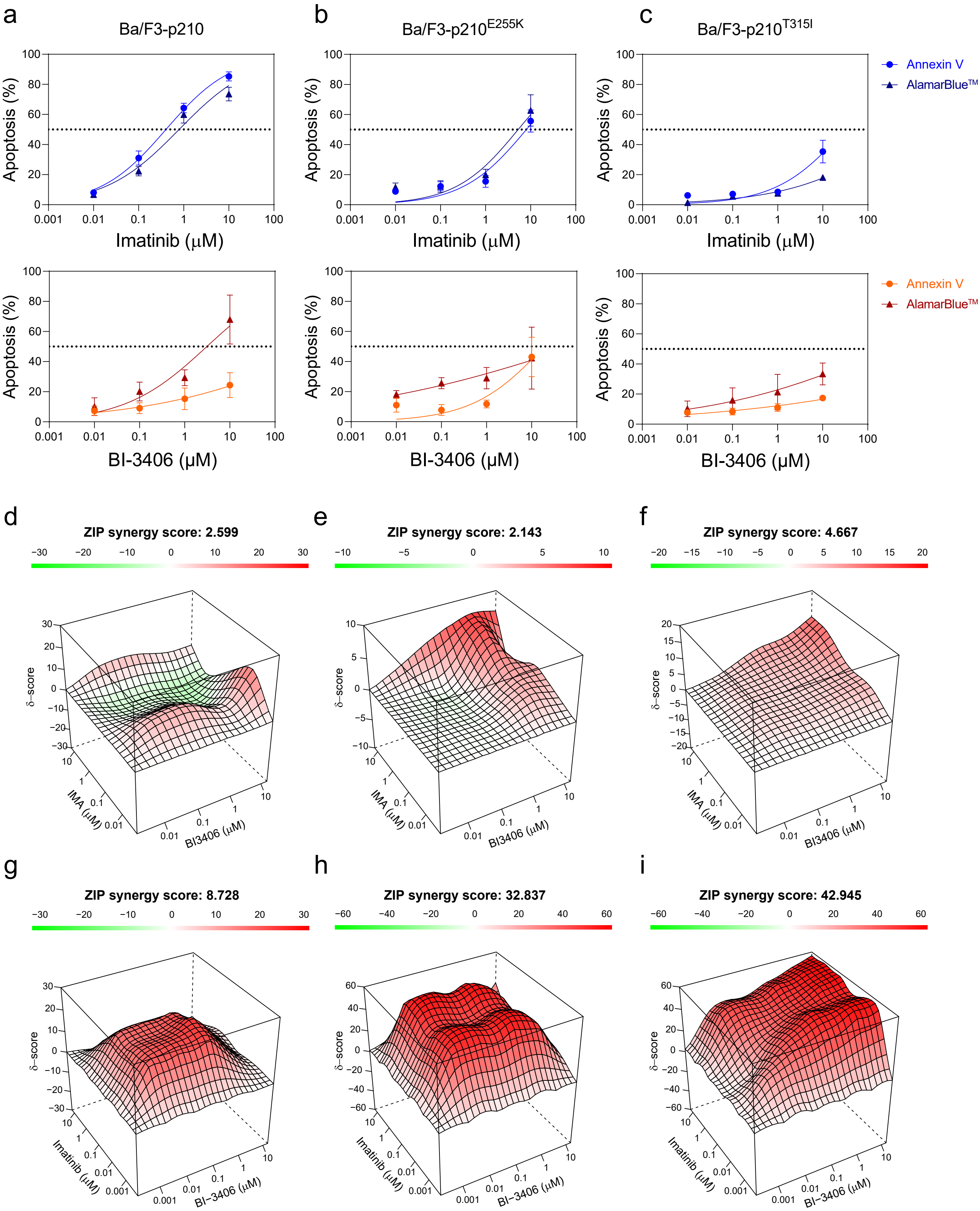

j

	IC50 (μM)	
	Annexin V	AlamarBlue™
BaF/3-p210 IMA	0.40±0.036	0.78±0.18
BaF/3-p210 BI-3406	1.57±0.76	3.10±0.99
BaF/3-p210^{E255K} IMA	8.01±2.42	5.28±1.80
BaF/3-p210^{E255K} BI-3406	>10	>10
BaF/3-p210^{T315I} IMA	>10	>10
BaF/3-p210^{T315I} BI-3406	>10	>10

Supplementary Figure S3. **BI-3406 enhances imatinib sensitivity in Ba/F3 murine pro-B cells expressing wild-type or mutant TKI-resistant BCR::ABL1 variants.**

(a-c) Dose–response curves for imatinib (upper panels) and BI-3406 (lower panels) in Ba/F3-p210 cells expressing wild-type (WT) (a), E255K (b), or T315I (c) BCR::ABL1, assessed by Annexin V apoptosis assays (Annexin V) or AlamarBlue™.

(d-f) ZIP synergy scores for each genotype based on Annexin V data. Co-treatment with BI-3406 moderately improved sensitivity in both mutants, yielding additive ZIP synergy scores (E255K: 2.14 and T315I: 4.667).

(g-i) Synergy map (ZIP model) of imatinib + BI-3406 in Ba/F3-p210 (g) E255K (h) and T315I (i) cells using AlamarBlue™-based viability assays. The combination elicited markedly synergistic responses in E255K cells (ZIP: 32.837, h) and T315I cells (ZIP: 42.945, i), whereas non-resistant Ba/F3-p210 cells showed only borderline synergy (ZIP < 10; g). Stronger synergy was observed in the most resistant T315I model, especially when assessed by metabolic readout.

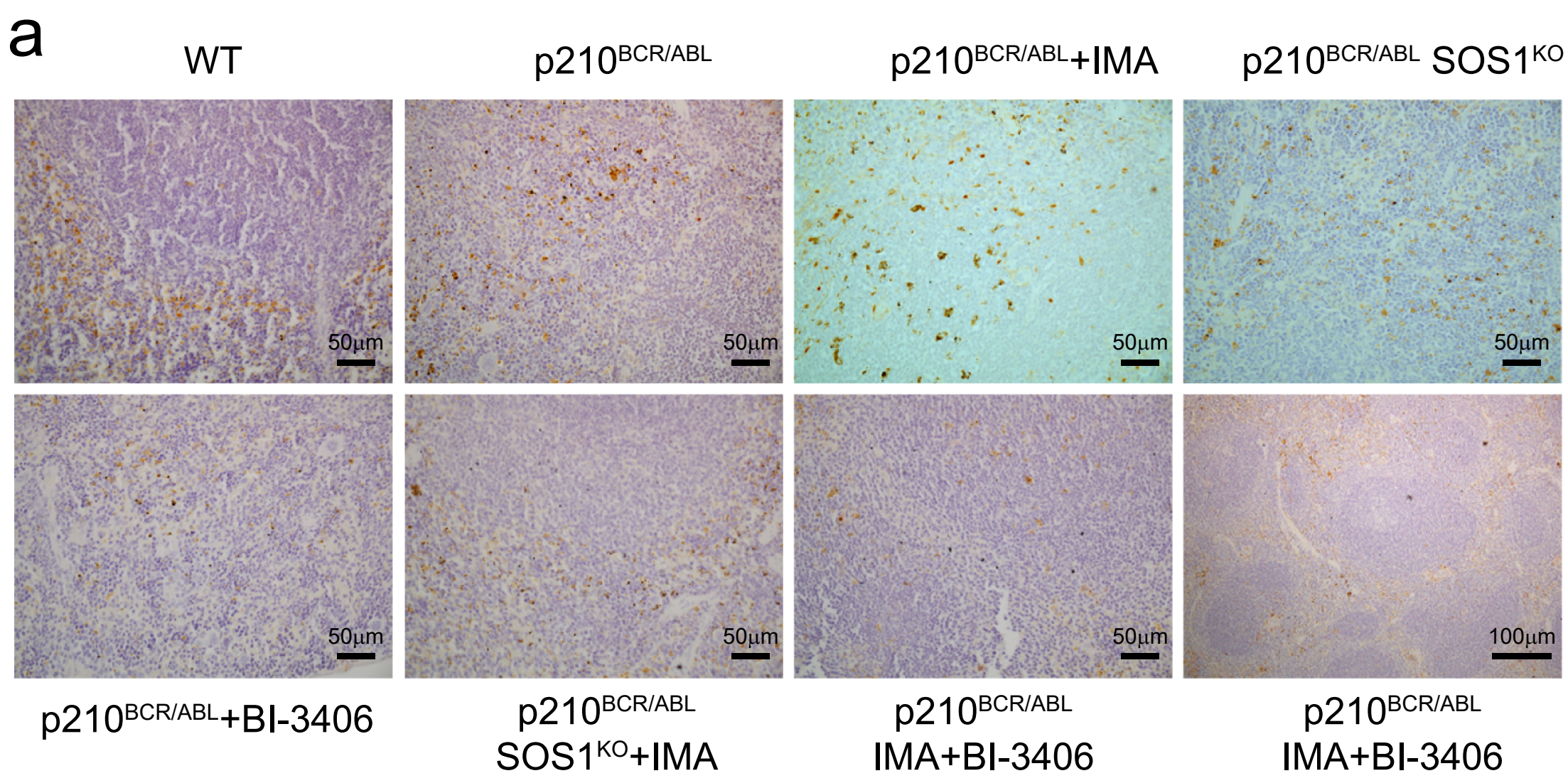
a
WT
p210BCR/ABL
p210BCR/ABL+IMA
p210BCR/ABL SOS1KO
50µm
100µm
p210BCR/ABL+BI-3406
p210BCR/ABL SOS1KO+IMA
p210BCR/ABL IMA+BI-3406
p210BCR/ABL IMA+BI-3406

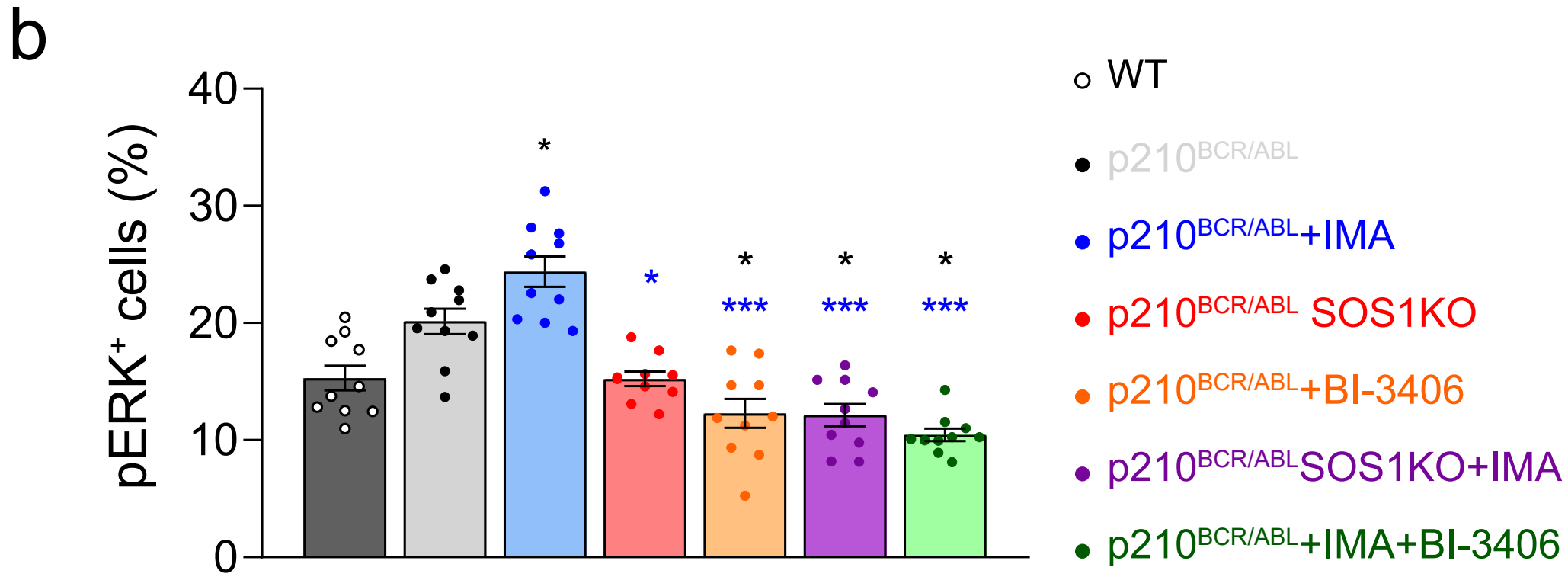
b
pERK+ cells (%)
40
30
20
10
0
*
*
***
WT
p210BCR/ABL
p210BCR/ABL+IMA
p210BCR/ABL SOS1KO
p210BCR/ABL+BI-3406
p210BCR/ABLSOS1KO+IMA
p210BCR/ABL+IMA+BI-3406

Supplementary Figure S4. Immunohistochemical detection of MAPK activation in the spleen of $p210^{BCR/ABL}$ mice undergoing SOS1 inhibition and/or BCR::ABL1 inhibition.

Combining either genetic or pharmacological SOS1 inhibition with BCR::ABL1 inhibition suppresses MAPK activation in the spleen of $p210^{BCR/ABL}$ mice.

(a) Representative immunohistochemistry images (anti-pERK, brown signal) of spleen tissue sections from the indicated treatment groups. Scale bars: 50 μm, and 100 μm.

(b) Quantification of phosphorylated ERK-positive ($pERK^+$) cells in spleen sections from p210BCR/ABL mice treated with vehicle, BI-3406, imatinib (IMA), or BI-3406+IMA was performed using QuPath and expressed as the percentage of $pERK^+$ cells. Imatinib monotherapy significantly increased the proportion of $pERK^+$ cells, consistent with compensatory MAPK activation. In contrast, BI-3406, either alone or in combination with imatinib, reduced ERK activation, with the strongest suppression observed in the dual-treatment group. Data represent mean ± SEM from 10 mice per group. Statistical significance was assessed by one-way ANOVA with Tukey's post-test. Black asterisks indicate comparisons versus vehicle-treated p210BCR/ABL mice, and blue asterisks indicate comparisons versus imatinib-treated p210BCR/ABL mice; *$p < 0.05$, **$p < 0.01$, ***$p < 0.001$.

Mouse p210$^{BCR/ABL}$

a

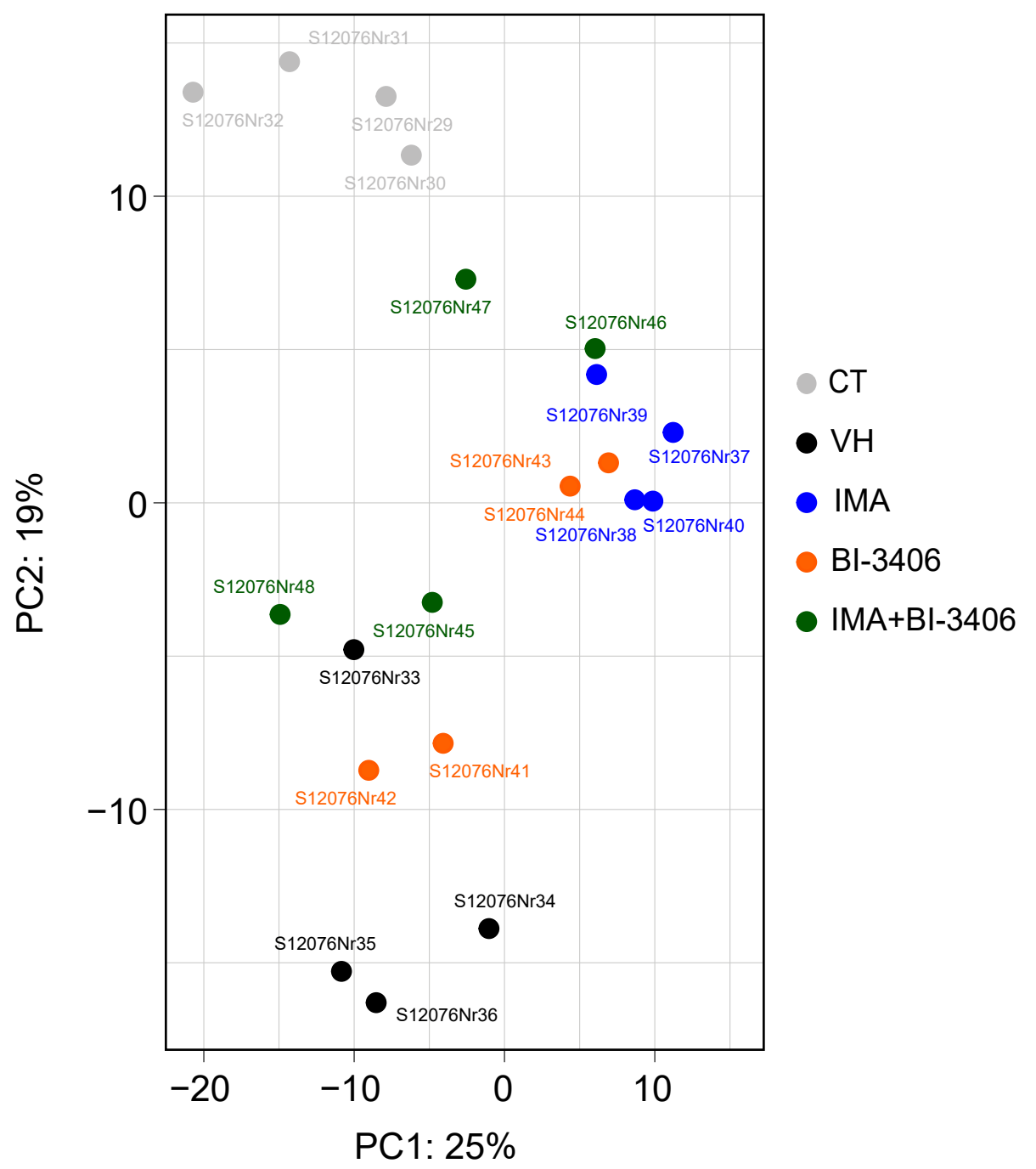

b

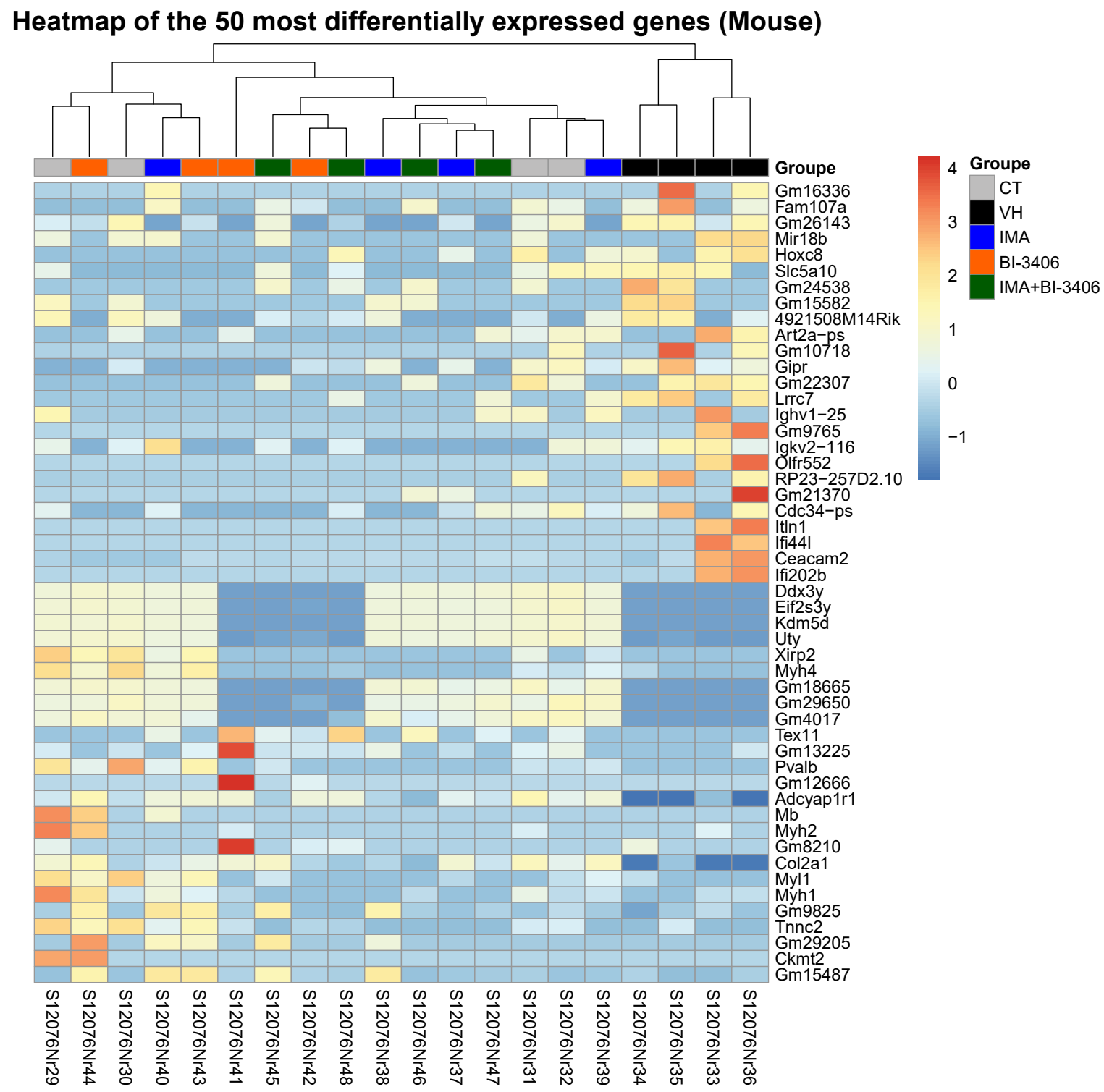

CML patients

c

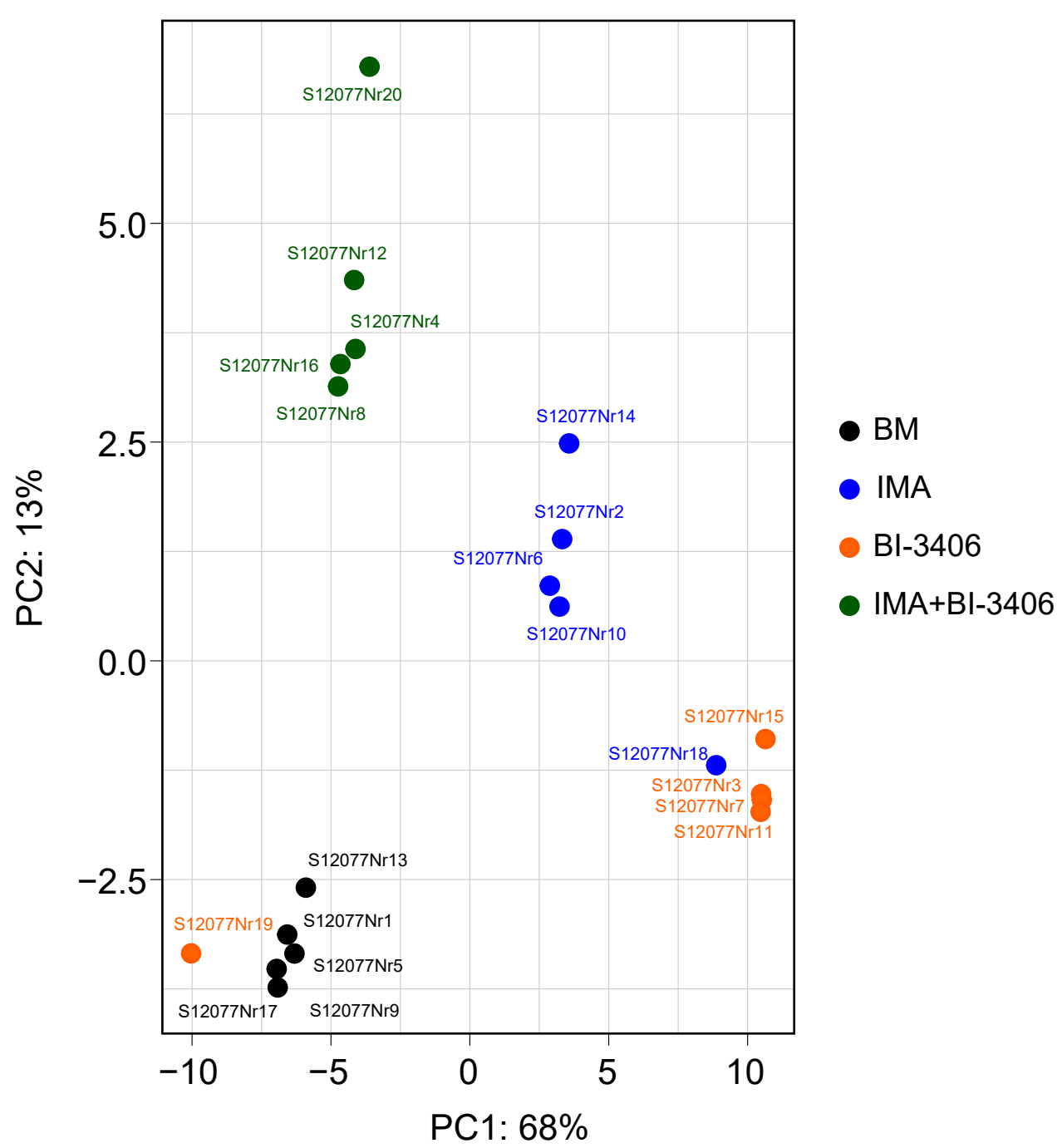

d

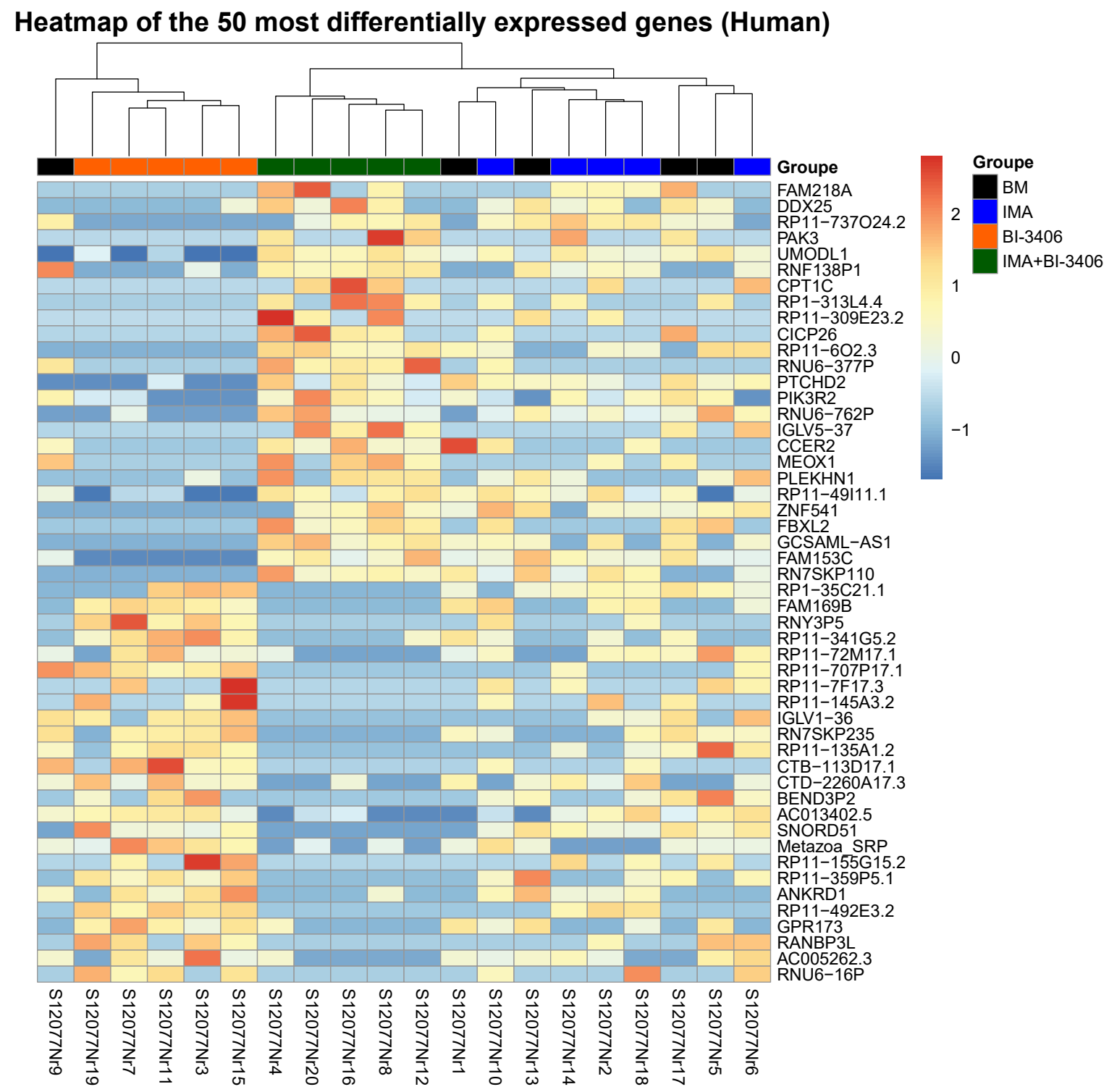

Supplementary Figure S5. Principal component analysis (PCA) and hierarchical clustering heatmaps of RNA-seq data from CML mouse and human bone marrow samples.

(a,b) PCA and hierarchical clustering heatmap of variance-stabilized RNA-seq expression data from bone marrow cells of $p210^{BCR/ABL}$ transgenic mice treated *in vivo* with vehicle control (VH), BI-3406, imatinib (IMA), or BI-3406 plus imatinib (IMA+BI-3406).

(c,d) Equivalent analyses for primary bone marrow mononuclear cells from CML patients treated *ex vivo* under the same conditions.

PCA plots (a,c) display the first two principal components explaining the largest proportion of variance, with each point representing an individual biological replicate. Heatmaps (b,d) show the top 50 differentially expressed genes (25 upregulated and 25 downregulated; FDR < 0.05) ranked by adjusted p-value, with color intensity indicating standardized expression values (z-scores).

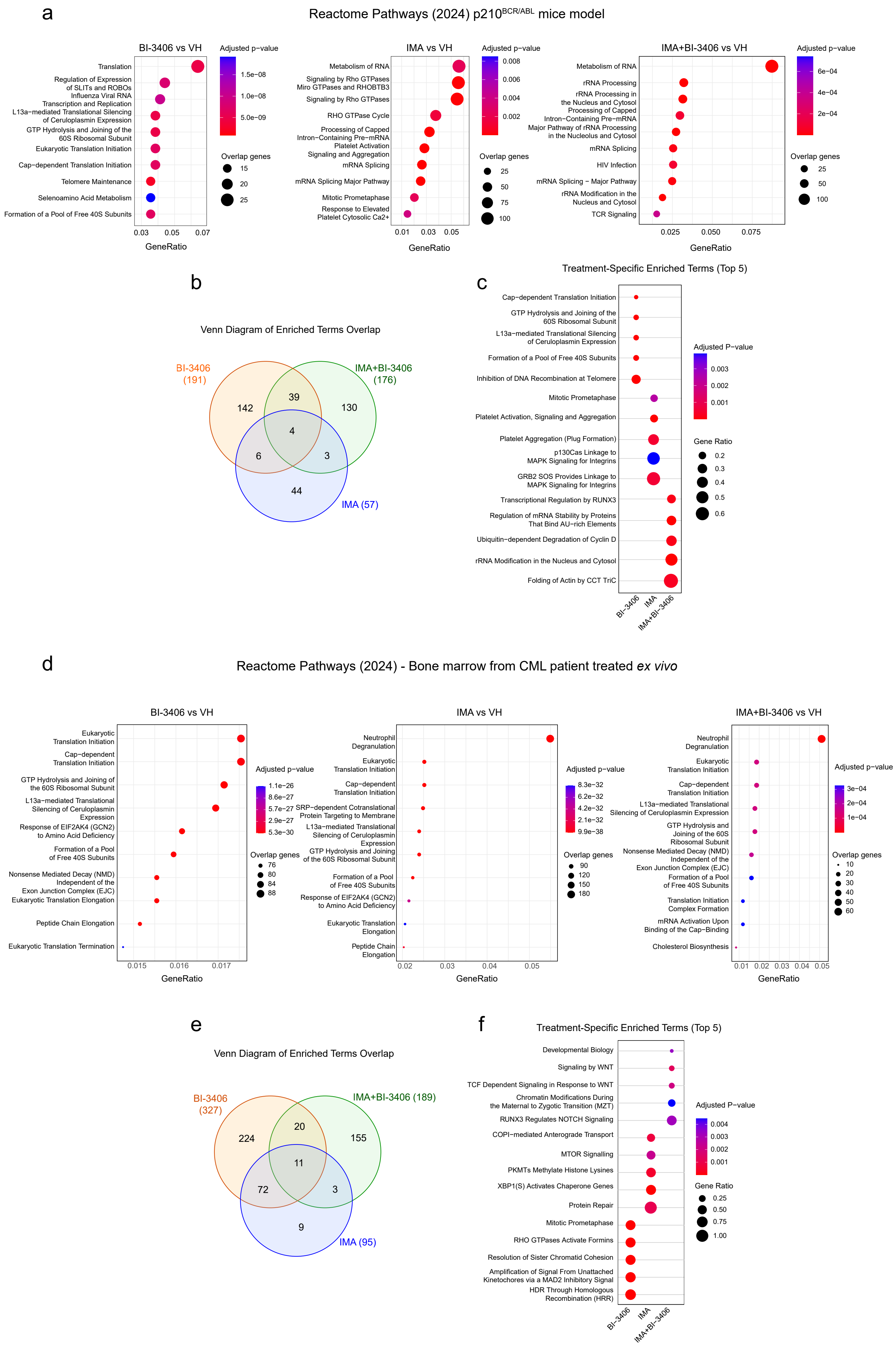
a
Reactome Pathways (2024) p210BCR/ABL mice model
BI-3406 vs VH
Translation
Regulation of Expression of SLITs and ROBOs
Influenza Viral RNA Transcription and Replication
L13a-mediated Translational Silencing of Ceruloplasmin Expression
GTP Hydrolysis and Joining of the 60S Ribosomal Subunit
Eukaryotic Translation Initiation
Cap-dependent Translation Initiation
Telomere Maintenance
Selenoamino Acid Metabolism
Formation of a Pool of Free 40S Subunits
GeneRatio
Adjusted p-value
Overlap genes
IMA vs VH
Metabolism of RNA
Signaling by Rho GTPases Miro GTPases and RHOBTB3
Signaling by Rho GTPases
RHO GTPase Cycle
Processing of Capped Intron-Containing Pre-mRNA
Platelet Activation Signaling and Aggregation
mRNA Splicing
mRNA Splicing Major Pathway
Mitotic Prometaphase
Response to Elevated Platelet Cytosolic Ca2+
IMA+BI-3406 vs VH
Metabolism of RNA
rRNA Processing
rRNA Processing in the Nucleus and Cytosol
Processing of Capped Intron-Containing Pre-mRNA
Major Pathway of rRNA Processing in the Nucleolus and Cytosol
mRNA Splicing
HIV Infection
mRNA Splicing - Major Pathway
rRNA Modification in the Nucleus and Cytosol
TCR Signaling
b
Venn Diagram of Enriched Terms Overlap
BI-3406 (191)
IMA+BI-3406 (176)
IMA (57)
c
Treatment-Specific Enriched Terms (Top 5)
Cap-dependent Translation Initiation
GTP Hydrolysis and Joining of the 60S Ribosomal Subunit
L13a-mediated Translational Silencing of Ceruloplasmin Expression
Formation of a Pool of Free 40S Subunits
Inhibition of DNA Recombination at Telomere
Mitotic Prometaphase
Platelet Activation, Signaling and Aggregation
Platelet Aggregation (Plug Formation)
p130Cas Linkage to MAPK Signaling for Integrins
GRB2 SOS Provides Linkage to MAPK Signaling for Integrins
Transcriptional Regulation by RUNX3
Regulation of mRNA Stability by Proteins That Bind AU-rich Elements
Ubiquitin-dependent Degradation of Cyclin D
rRNA Modification in the Nucleus and Cytosol
Folding of Actin by CCT TriC
Adjusted P-value
Gene Ratio
BI-3406
IMA
IMA+BI-3406
d
Reactome Pathways (2024) - Bone marrow from CML patient treated ex vivo
BI-3406 vs VH
Eukaryotic Translation Initiation
Cap-dependent Translation Initiation
GTP Hydrolysis and Joining of the 60S Ribosomal Subunit
L13a-mediated Translational Silencing of Ceruloplasmin Expression
Response of EIF2AK4 (GCN2) to Amino Acid Deficiency
Formation of a Pool of Free 40S Subunits
Nonsense Mediated Decay (NMD) Independent of the Exon Junction Complex (EJC)
Eukaryotic Translation Elongation
Peptide Chain Elongation
Eukaryotic Translation Termination
IMA vs VH
Neutrophil Degranulation
Eukaryotic Translation Initiation
Cap-dependent Translation Initiation
SRP-dependent Cotranslational Protein Targeting to Membrane
L13a-mediated Translational Silencing of Ceruloplasmin Expression
GTP Hydrolysis and Joining of the 60S Ribosomal Subunit
Formation of a Pool of Free 40S Subunits
Response of EIF2AK4 (GCN2) to Amino Acid Deficiency
Eukaryotic Translation Elongation
Peptide Chain Elongation
IMA+BI-3406 vs VH
Neutrophil Degranulation
Eukaryotic Translation Initiation
Cap-dependent Translation Initiation
L13a-mediated Translational Silencing of Ceruloplasmin Expression
GTP Hydrolysis and Joining of the 60S Ribosomal Subunit
Nonsense Mediated Decay (NMD) Independent of the Exon Junction Complex (EJC)
Formation of a Pool of Free 40S Subunits
Translation Initiation Complex Formation
mRNA Activation Upon Binding of the Cap-Binding
Cholesterol Biosynthesis
e
Venn Diagram of Enriched Terms Overlap
BI-3406 (327)
IMA+BI-3406 (189)
IMA (95)
f
Treatment-Specific Enriched Terms (Top 5)
Developmental Biology
Signaling by WNT
TCF Dependent Signaling in Response to WNT
Chromatin Modifications During the Maternal to Zygotic Transition (MZT)
RUNX3 Regulates NOTCH Signaling
COPI-mediated Anterograde Transport
MTOR Signalling
PKMTs Methylate Histone Lysines
XBP1(S) Activates Chaperone Genes
Protein Repair
Mitotic Prometaphase
RHO GTPases Activate Formins
Resolution of Sister Chromatid Cohesion
Amplification of Signal From Unattached Kinetochores via a MAD2 Inhibitory Signal
HDR Through Homologous Recombination (HRR)
Adjusted P-value
Gene Ratio

Supplementary Figure S6. Comparative analysis of Reactome Pathway 2024 in transcriptomes from the bone marrow of CML mice and human patients treated with BI-3406, imatinib, or their combination.

(a,d) Dot plots showing the top 10 Reactome-enriched pathways (FDR < 0.05) identified in differentially expressed genes (DEGs) from bone marrow samples. The samples were obtained from (a) $p210^{BCR/ABL}$ CML mice treated *in vivo* and (d) CML patient cells treated *ex vivo* with BI-3406, imatinib (IMA), or the combination (IMA+BI-3406). The control group for all comparisons was the vehicle (VH), which consisted of Natrosol and saline for the *in vivo* experiment and DMSO for the *ex vivo* studies. Circle size represents the number of overlap genes, and the color scale reflects the adjusted p-value.

(b,e) Venn diagrams displaying the number of shared and unique Reactome pathways enriched in each treatment condition for mouse (b) and human (e) samples.

(c,f) Dot plots showing the top 5 representative pathways specifically enriched in each individual treatment (BI-3406, IMA, or IMA+BI-3406), highlighting unique transcriptional programs induced by monotherapies or combination, in both mouse (c) and human (f) bone marrow. Circle size indicates gene ratio; color scale reflects adjusted p-value.

Enrichment analyses were performed using Enrichr with Reactome Pathway 2024 database input. Gene lists were generated from DESeq2 with FDR < 0.05. Shared and specific pathway identities were defined by overlap among enriched term sets.

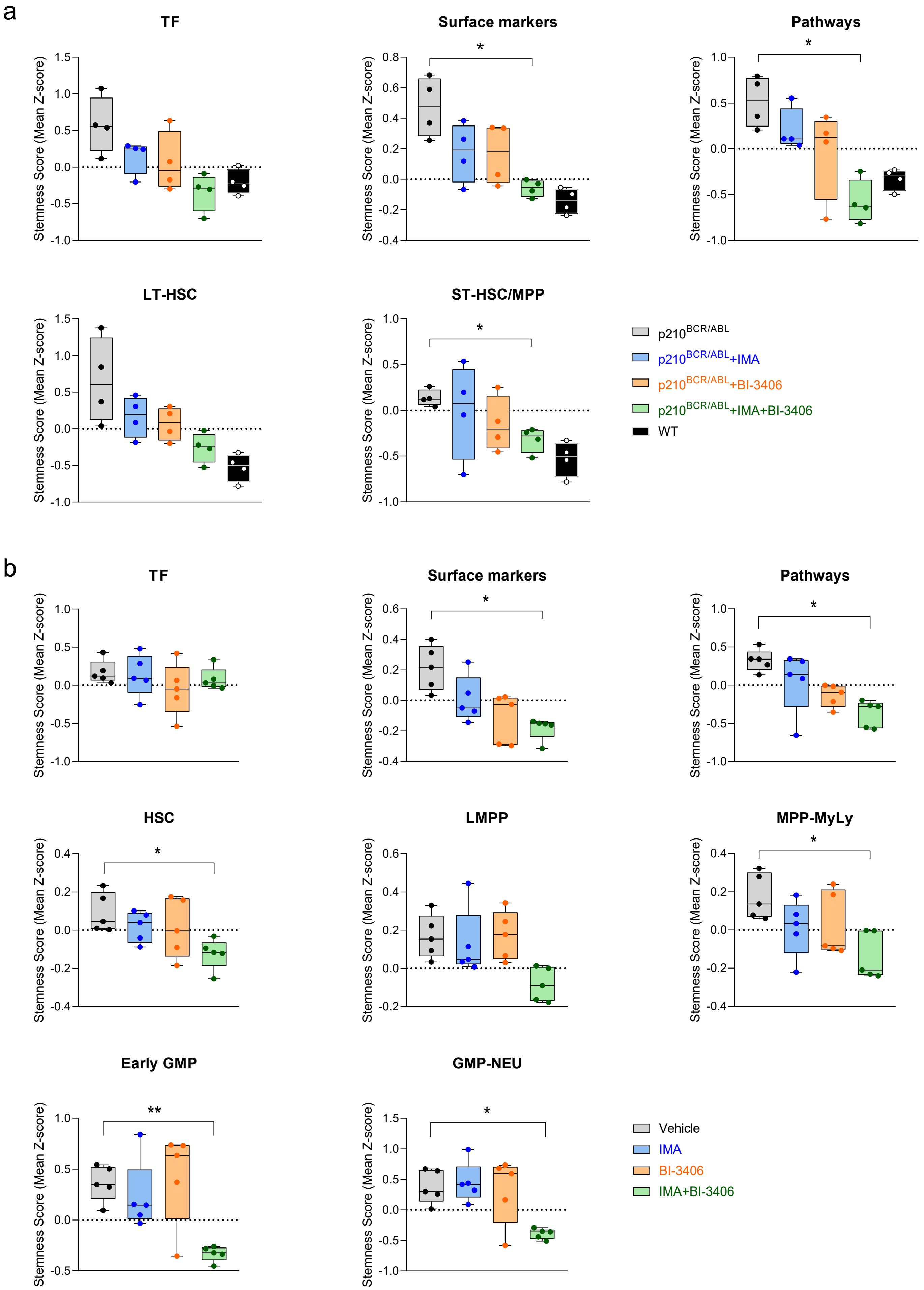

a
TF
Surface markers
Pathways
LT-HSC
ST-HSC/MPP
Stemness Score (Mean Z-score)
*
p210BCR/ABL
p210BCR/ABL+IMA
p210BCR/ABL+BI-3406
p210BCR/ABL+IMA+BI-3406
WT
b
TF
Surface markers
Pathways
HSC
LMPP
MPP-MyLy
Early GMP
GMP-NEU
Stemness Score (Mean Z-score)
*
**
Vehicle
IMA
BI-3406
IMA+BI-3406

Supplementary Figure S7. Modulation of stemness signatures in murine and human CML bone marrow after single or combined BI-3406 and imatinib therapy treatments.

(a) Z-score-based stemness scores in bone marrow from $p210^{BCR/ABL}$ CML mice treated in vivo with vehicle (VH), imatinib (IMA), BI-3406 or the combination (IMA+BI-3406), together with non-leukemic control (WT) mice. Scores were calculated from bulk RNA-seq using curated murine modules encompassing Transcription Factor-stemness (TF), HSC/LSC-associated surface markers (Surface markers), stemness-associated pathways (Wnt/β-catenin, Hedgehog, Notch) and LT-HSC and ST-HSC/MPP signatures. Positive values indicate relative enrichment of the corresponding program compared with the dataset mean.

(b) Z-score-based stemness scores in *ex vivo*-treated BMMNCs from CML patients exposed to vehicle (control, DMSO only), BI-3406, IMA or IMA+BI-3406. Human modules include TF-stemness (TF), generic HSC/LSC surface markers (Surface markers), stemness-associated pathways (Pathways: Wnt/β-catenin, Hedgehog, Notch) and selected cell-state signatures (HSC, MPP-MyLy, LMPP, early GMP, GMP-NEU) derived from the BoneMarrowMap single-cell reference atlas of human hematopoiesis.

Scores were calculated by transforming normalized expression values to gene-wise Z-scores and averaging across genes within each module (see Supplementary Methods). Statistical significance: *$p < 0.05$, **$p < 0.01$; one-way ANOVA with Tukey's post-hoc test.

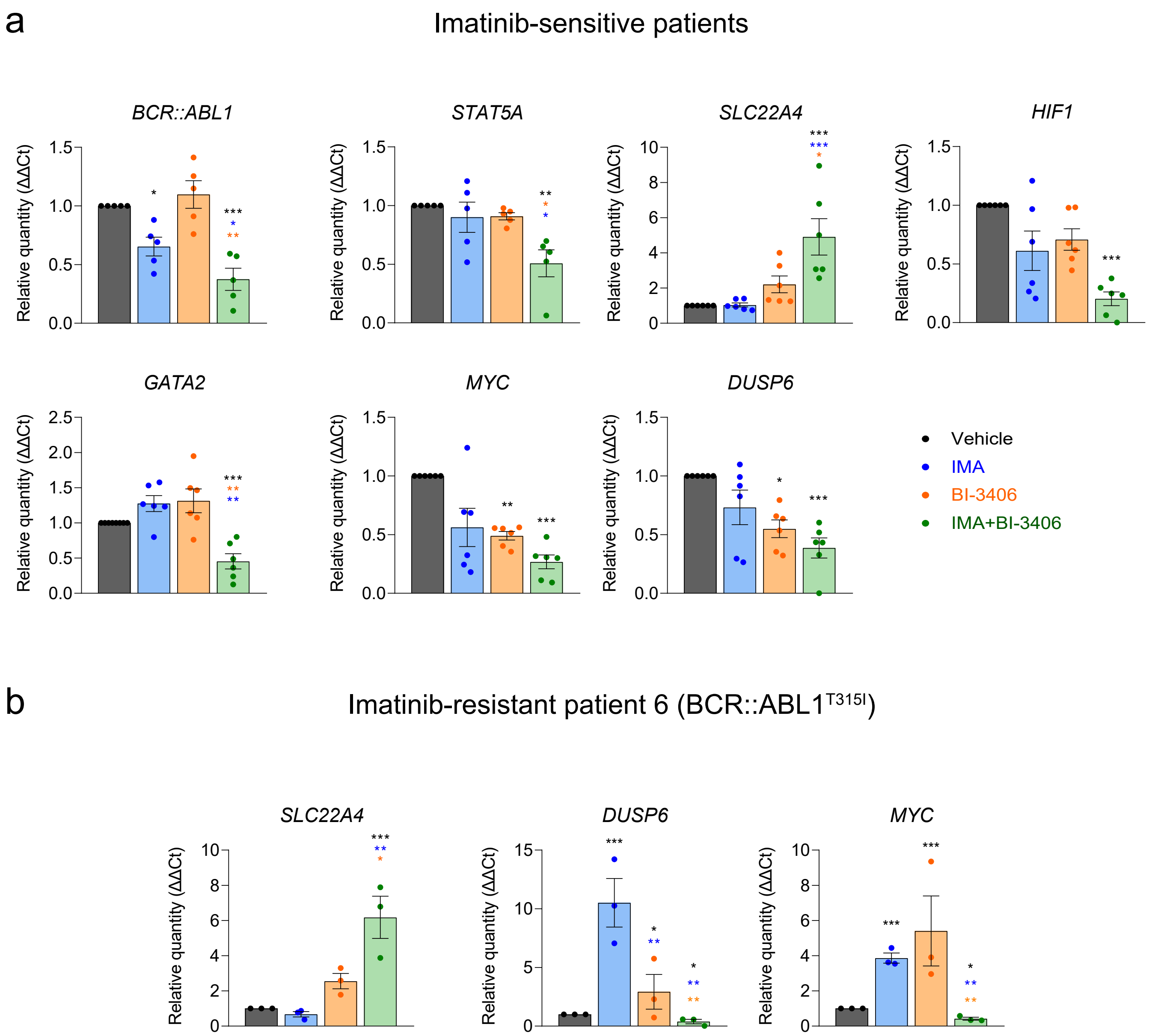

a
Imatinib-sensitive patients
BCR::ABL1
STAT5A
SLC22A4
HIF1
GATA2
MYC
DUSP6
Relative quantity (ΔΔCt)
Vehicle
IMA
BI-3406
IMA+BI-3406
b
Imatinib-resistant patient 6 (BCR::ABL1T315I)
SLC22A4
DUSP6
MYC
Relative quantity (ΔΔCt)

Supplementary Figure S8. Direct RT–qPCR validation of transcriptomic alterations detected in RNA-seq studies of the bone marrow of imatinib-sensitive (patients #1-5) and imatinib-resistant (patient #6) CML patients

(a) Quantitative PCR analysis of *BCR::ABL1, SLC22A4, GATA2, DUSP6, MYC and HIF1A* in ex vivo bone-marrow–derived mononuclear cells (BMMNCs) from CML patients treated with vehicle (control, DMSO only), BI-3406, imatinib (IMA) or the combination (IMA+BI-3406). Data are shown as relative quantity (ΔΔCt) normalized to 18S rRNA and expressed versus non-treated. BI-3406 monotherapy significantly induced SLC22A4, with the highest up-regulation under IMA+BI-3406. *MYC* and *BCR::ABL1* decreased across treatments, with the strongest suppression under IMA+BI. GATA2 and HIF1A were significantly reduced only in the combination arm, consistent with suppression of stemness and hypoxia-adaptation programs.

(b) Case study in an imatinib-resistant T315I CML patient. qPCR for *SLC22A4, DUSP6* and *MYC* in ex vivo BMMNCs shows *DUSP6* and *MYC* induction with IMA monotherapy (compatible with compensatory MAPK activation), partial reduction with BI-3406, and marked suppression below VH with IMA+BI-3406. SLC22A4 increased in all arms, peaking with IMA+BI. Dots represent technical replicates from the same patient.

Statistical significance: *$p < 0.05$, **$p < 0.01$, ***$p < 0.001$; one-way ANOVA with Tukey's post-hoc test. Black asterisks indicate differences versus vehicle (control, DMSO only), blue asterisks versus IMA, and orange asterisks versus BI-3406. Error bars: mean ± SEM. Panel (a): biological replicates (n = 5 per group). Panel (b): n = 1 patient (3 technical replicates/condition). These results validate key RNA-seq trends and support cooperative transcriptional reprogramming by IMA+BI-3406, including attenuation of MAPK-associated transcriptional outputs in the T315I context.

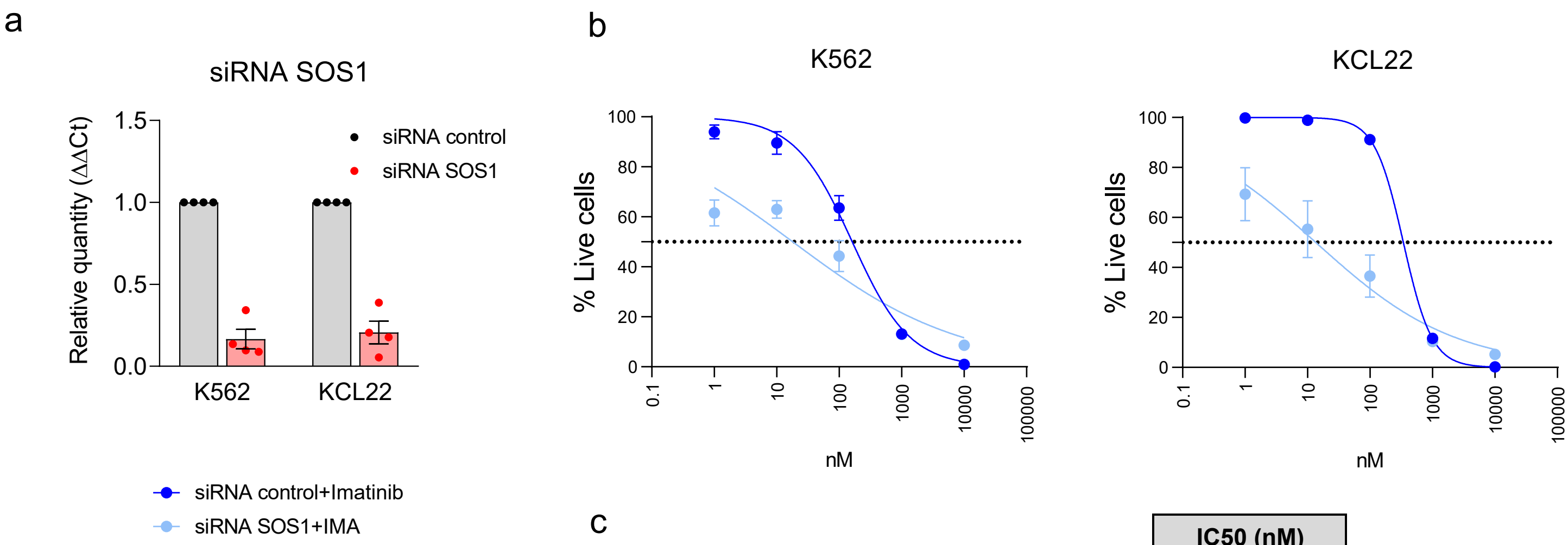

c

		IC50 (nM)
**siRNA**	**Cell line**	**Imatinib**
Control	K562	161.8±21.2
	KCL22	342.2±15.7
SOS1	K562	18.0±6.2
	KCL22	13.5±6.7

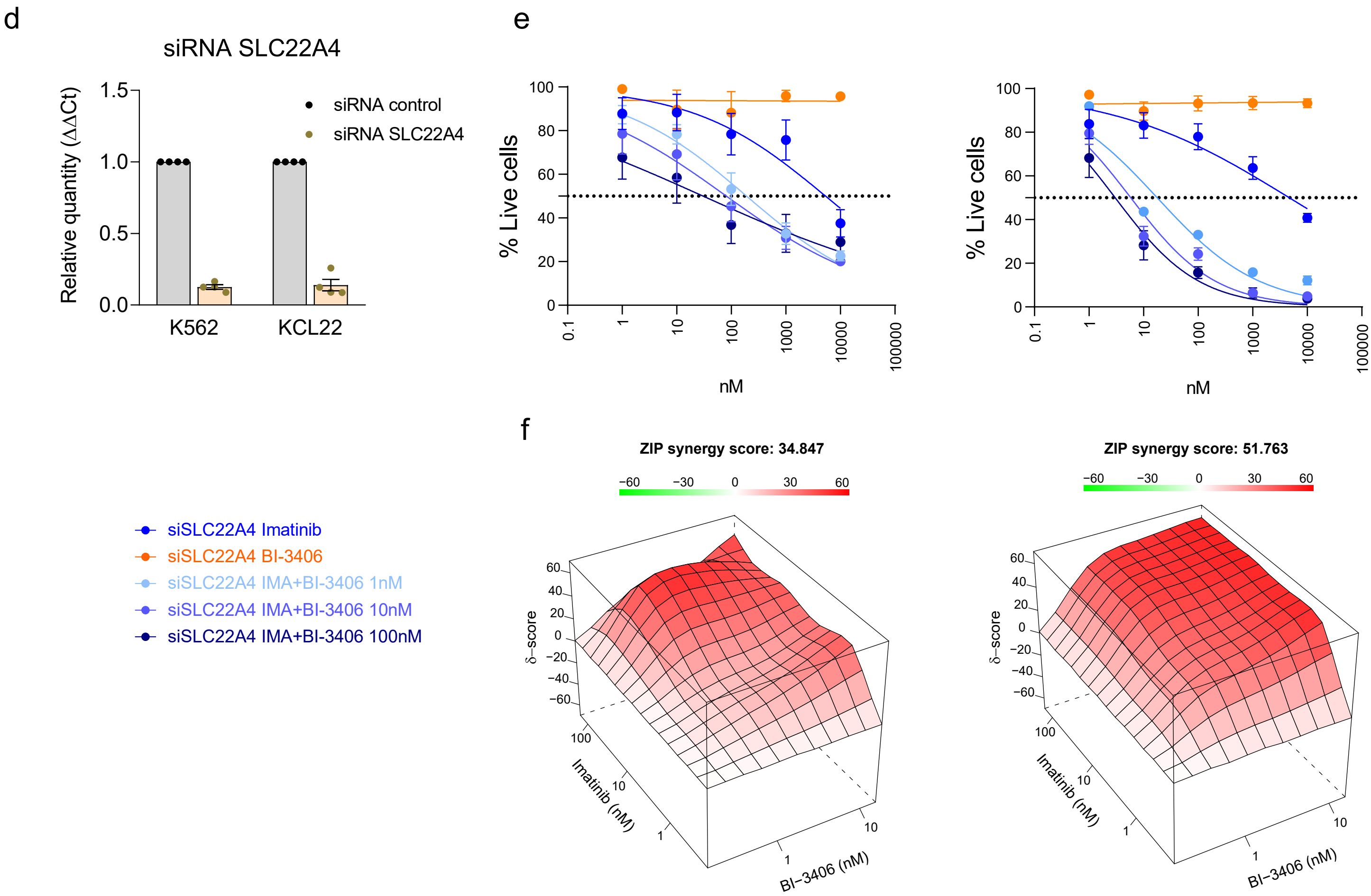

g

		IC50 (nM)				
**siRNA**	**Cell line**	**Imatinib**	**BI-3406**	**IMA+BI-3406 1nM**	**IMA+BI-3406 10nM**	**IMA+BI-3406 100nM**
Control	K562	281.3±45.4	8714±813	85.2±42.1	34.8±20.2	16.9±6.8
	KCL22	373.1±52.1	4035±243.4	126.1±21.8	6.0±3.2	1±0.2
SLC22A4	K562	5346±3862	>10000	190.8±58.6	81.3±43.4	30.4±9.8
	KCL22	4688±2811	>10000	17.8±3.6	5.8±1.2	3.0±0.8

Supplementary Figure S9. SOS1 and SLC22A4 knockdown analyses in CML cell lines.

(a) Validation of SOS1 knockdown by RT-qPCR in K562 and KCL22 cells transfected with SOS1-targeting siRNA or non-targeting siRNA control. Relative SOS1 mRNA expression was calculated using the ΔΔCt method.

(b) Dose–response viability curves of K562 and KCL22 cells transfected with non-targeting siRNA control or SOS1-targeting siRNA and treated with increasing concentrations of imatinib (IMA). SOS1 knockdown increased imatinib sensitivity in both CML cell lines.

(c) Summary table of imatinib IC_{50} values in control and SOS1-depleted K562 and KCL22 cells.

(d) Validation of SLC22A4 knockdown by RT-qPCR in K562 and KCL22 cells transfected with SLC22A4-targeting siRNA or non-targeting siRNA control. Relative SLC22A4 mRNA expression was calculated using the ΔΔCt method.

(e) Dose–response viability curves of SLC22A4-depleted K562 and KCL22 cells treated with imatinib, BI-3406, or imatinib combined with fixed concentrations of BI-3406. SLC22A4 knockdown reduced imatinib sensitivity but did not abolish the cooperative effect of BI-3406 and imatinib.

(f) ZIP synergy analysis of imatinib + BI-3406 combinations in SLC22A4-depleted K562 and KCL22 cells using SynergyFinder. 3D surface plots show positive drug synergy after SLC22A4 knockdown, with ZIP synergy scores >10 indicating synergistic interaction.

(g) Summary table of IC_{50} values for imatinib, BI-3406 and the indicated imatinib + BI-3406 combinations in control and SLC22A4-depleted K562 and KCL22 cells.

Data are shown as mean ± SEM from four replicates. IC_{50} values were calculated using non-linear regression with a four-parameter logistic model in GraphPad Prism v8.0.

a

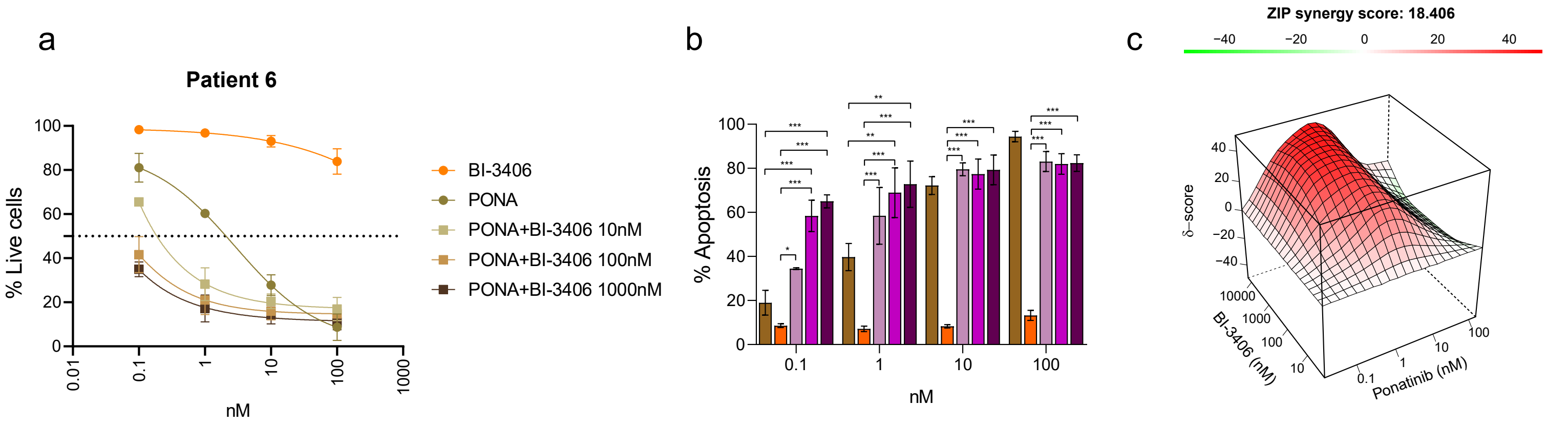

d

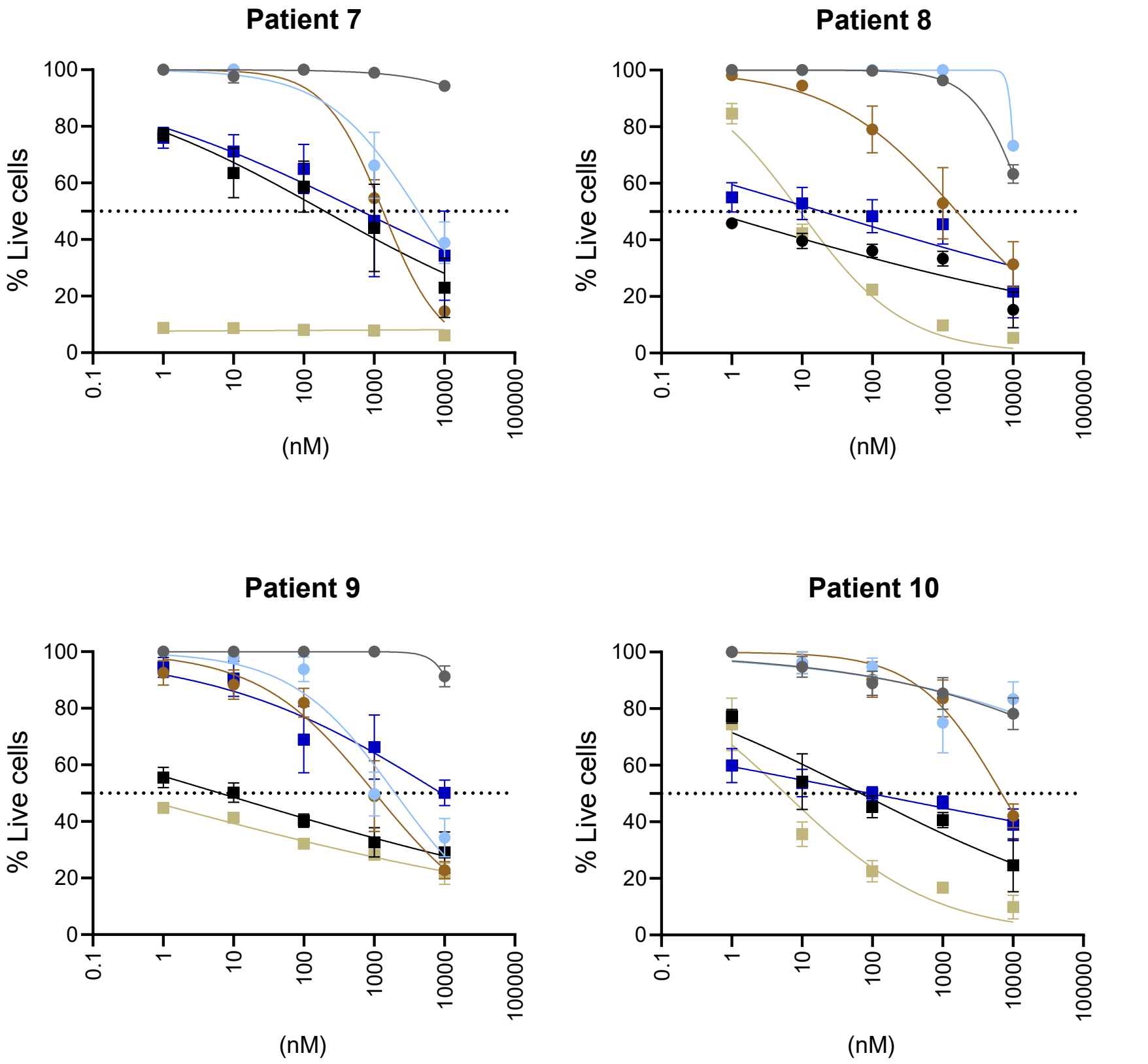

e

		IC50 (nM)		
Patient	TKI	TKI alone	TKI + BI-3406 100 nM	ZIP scores
7	Dasatinib	> 10000	199±57.2	44.61
	Nilotinib	4232±1282	661±75.7	38.18
	Ponatinib	1339±155	< 0.01	63.57
8	Dasatinib	> 10000	0.48±0.35	38.43
	Nilotinib	> 10000	19.3±8.4	32.97
	Ponatinib	1592±646	9.4±1.7	43.64
9	Dasatinib	> 10000	6.4±4.8	60.87
	Nilotinib	1985±588	895±87	25.95
	Ponatinib	1026±355	0.247±0.175	43.20
10	Dasatinib	> 10000	68.8±40.3	40.38
	Nilotinib	> 10000	93.8±80.7	34.81
	Ponatinib	6893.6±1574	5.8±2.2	56.77

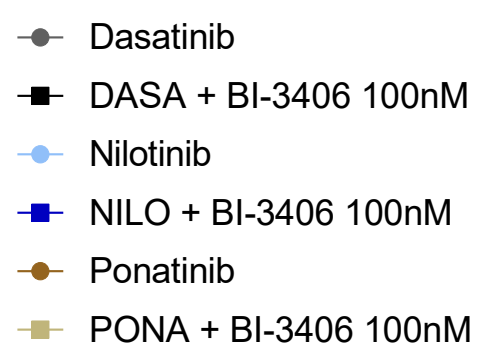

Supplementary Figure S10. BI-3406 enhances sensitivity to second- and third-generation TKIs in imatinib-resistant primary CML samples.

(a) Dose–response viability curve of BMMNCs from imatinib-resistant patient #6 harboring the BCR::ABL1 T315I mutation treated *ex vivo* with ponatinib (PONA), BI-3406, or ponatinib combined with fixed concentrations of BI-3406.

(b) Annexin V apoptosis analysis in patient #6 under the same ponatinib-based treatment conditions. Data are shown as mean ± SEM. Each dot represents a technical replicate from patient #6. Statistical significance was assessed by one-way ANOVA followed by Tukey's post-test; *p<0.05, **p<0.01, ***p<0.001.

(c) ZIP synergy surface plot showing the interaction between ponatinib and BI-3406 in patient #6.

(d) Dose–response viability curves of BMMNCs from clinically imatinib-resistant CML patients #7-10 treated *ex vivo* with dasatinib, nilotinib or ponatinib, alone or in combination with BI-3406 100 nM. Cell viability was expressed as the percentage of live cells relative to untreated controls.

(e) Summary table of IC50 values for dasatinib, nilotinib and ponatinib alone or in combination with BI-3406 100 nM in clinically imatinib-resistant CML patient samples, together with ZIP synergy scores for each combination. IC50 values were calculated using non-linear regression with a four-parameter logistic model in GraphPad Prism. Data are shown as mean ± SEM from technical replicates.
