# Supplementary material for: Pharmacological SOS1 inhibitor BI-3406 synergizes with BCR::ABL1 TKIs reducing leukemia stem cells and overcoming imatinib resistance in CML
Source: Cell Death Dis. 2026 Aug 12;17(1):951. doi: 10.1038/s41419-026-09170-6 (PMC13748536; doi:10.1038/s41419-026-09170-6)

Figure 6 a)

K562 RAS-GTP

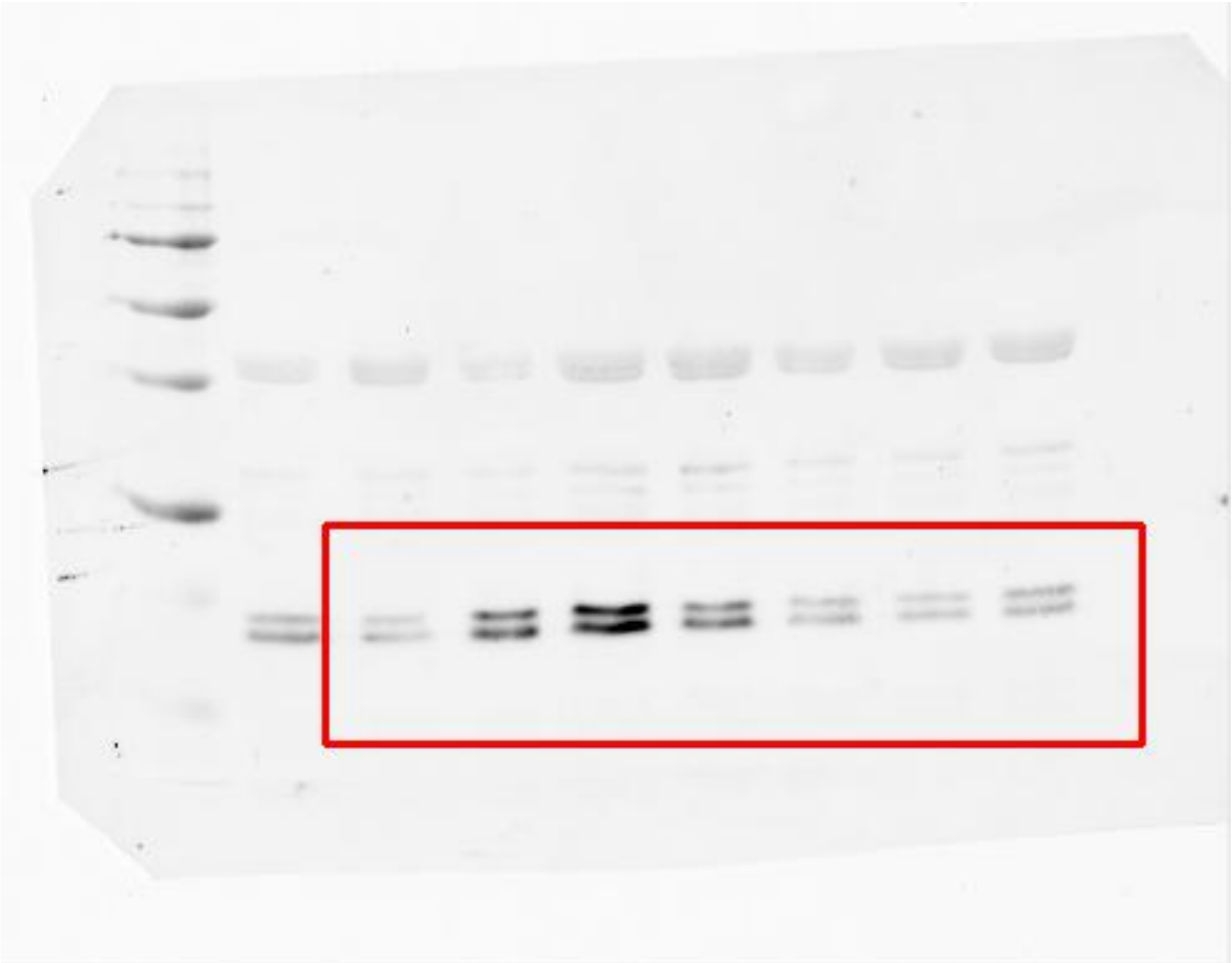

K562 RAS

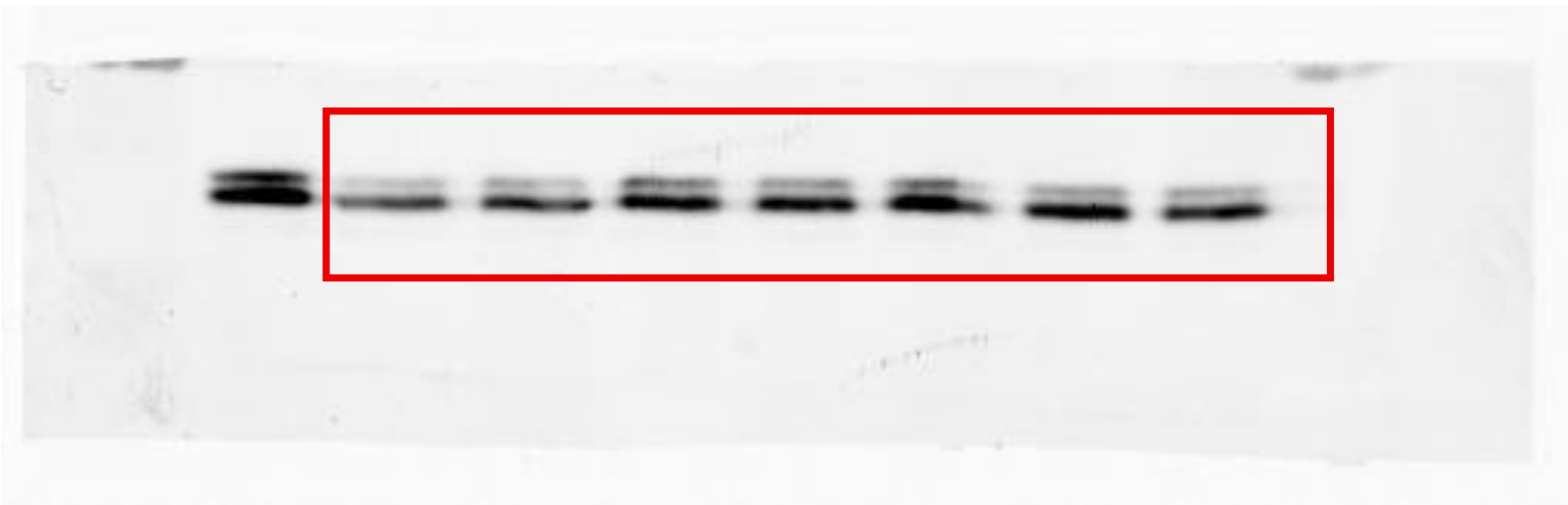

KCL22 RAS GTP

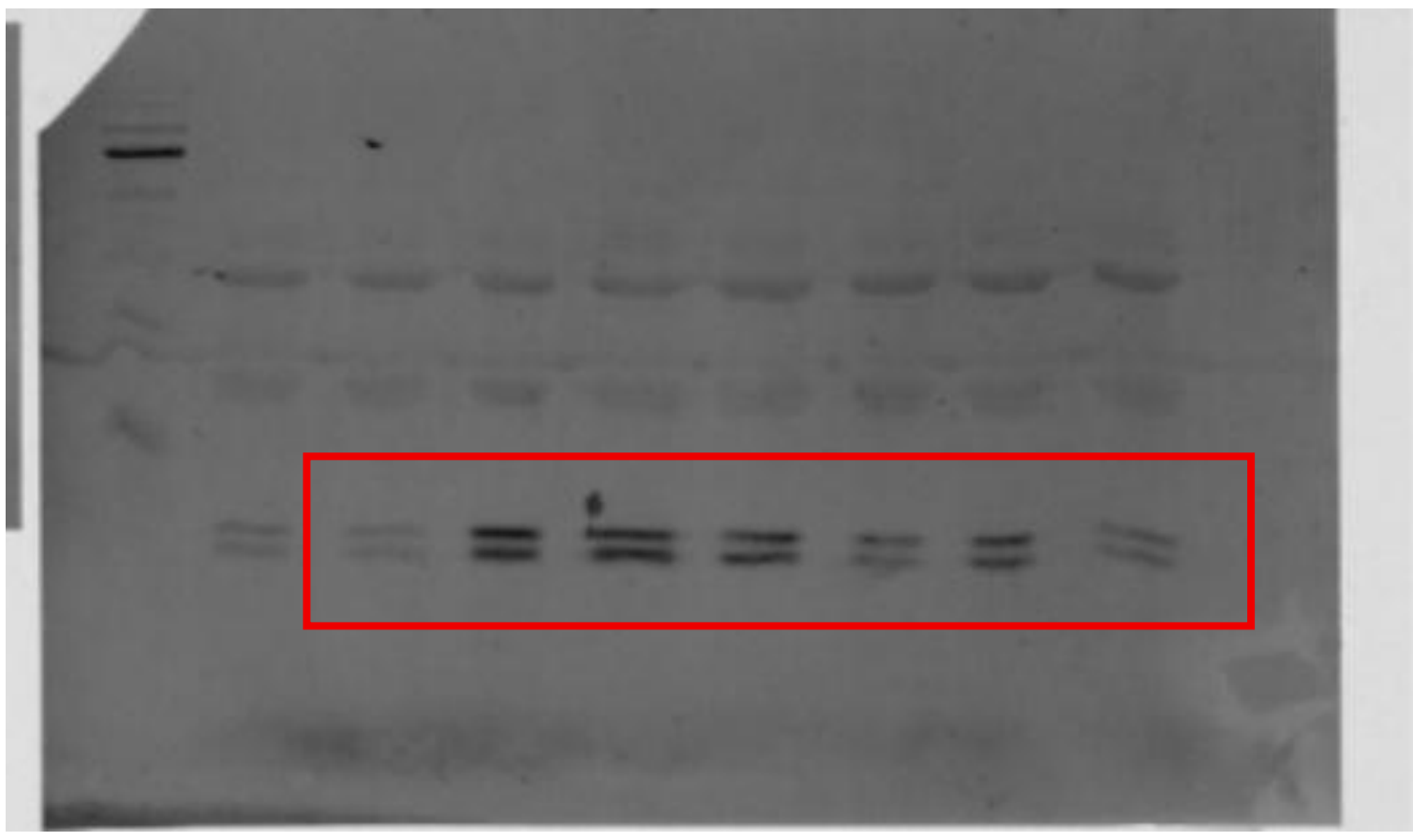

KCL22 RAS

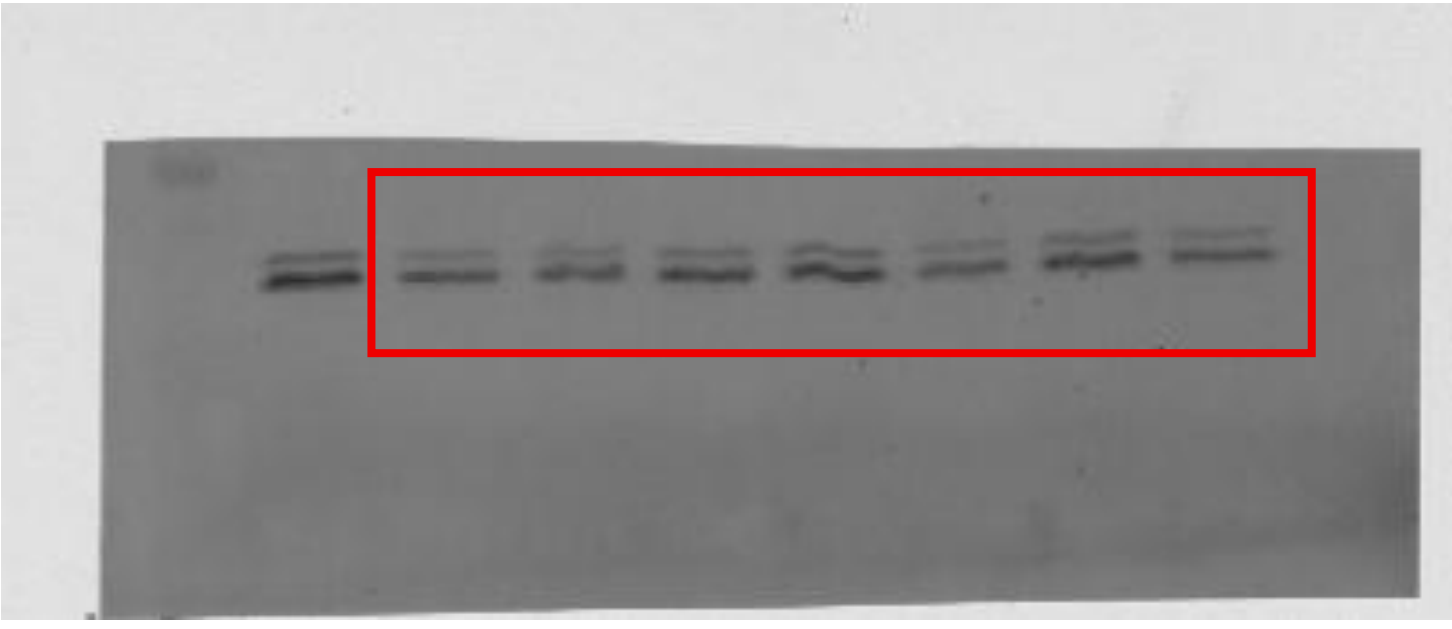

K562 RAC-GTP

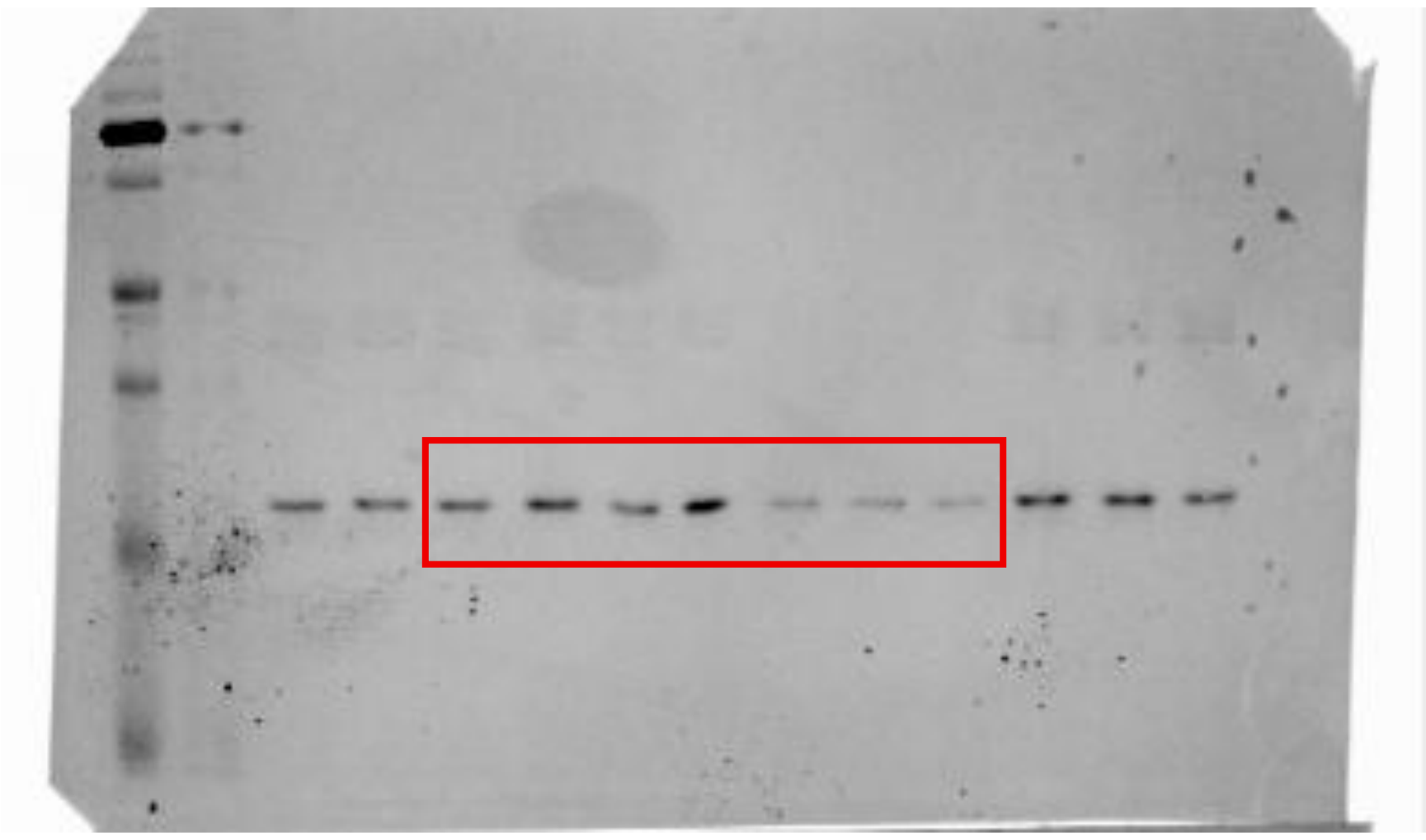

K562 RAC

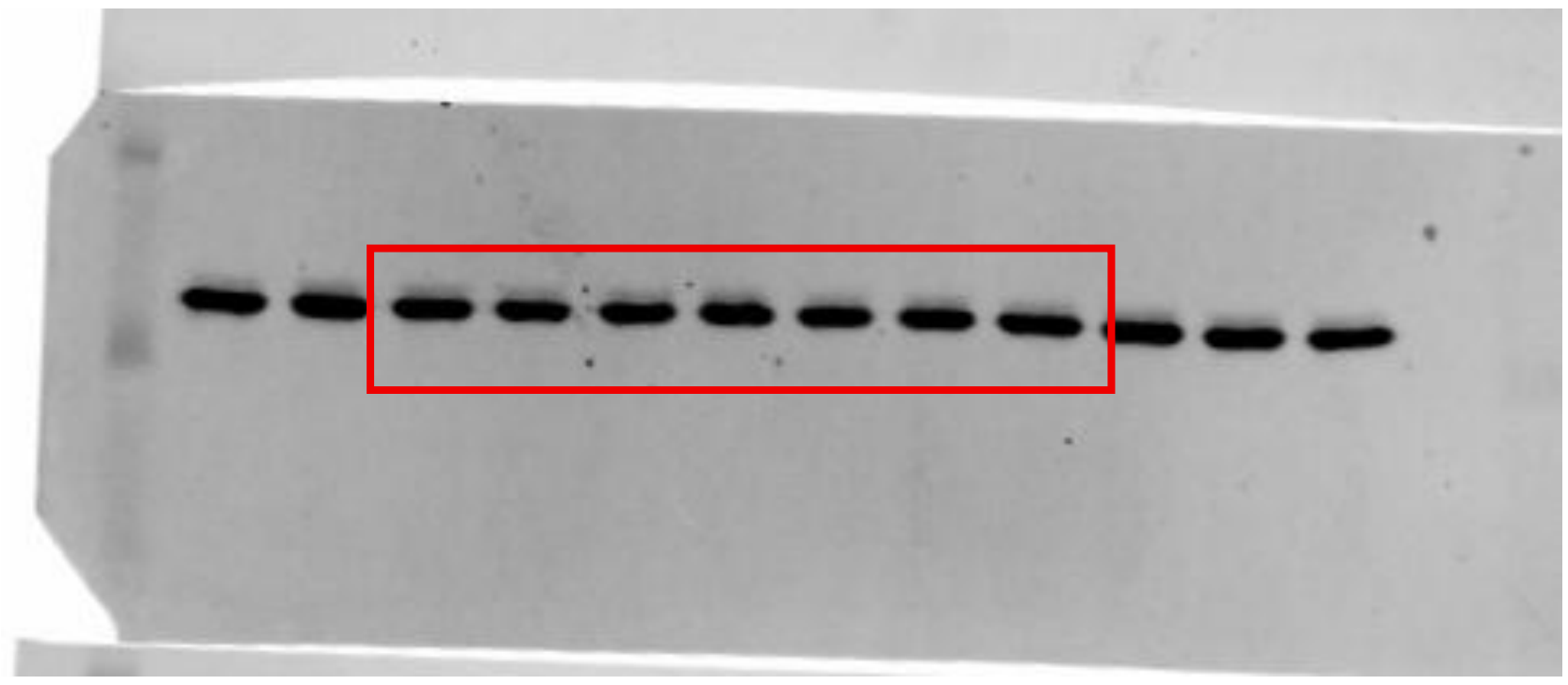

KCL22 RAC-GTP

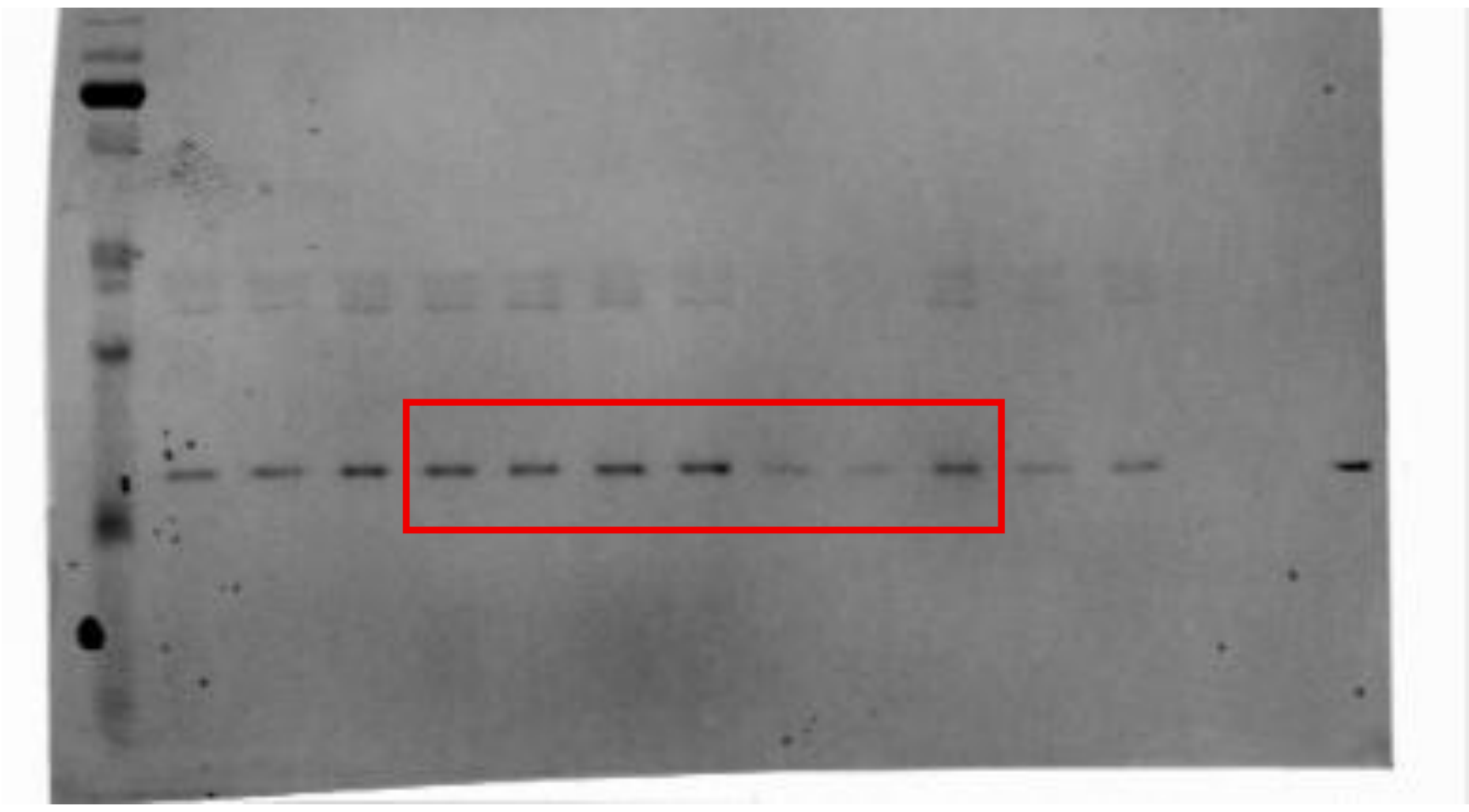

KCL22 RAC

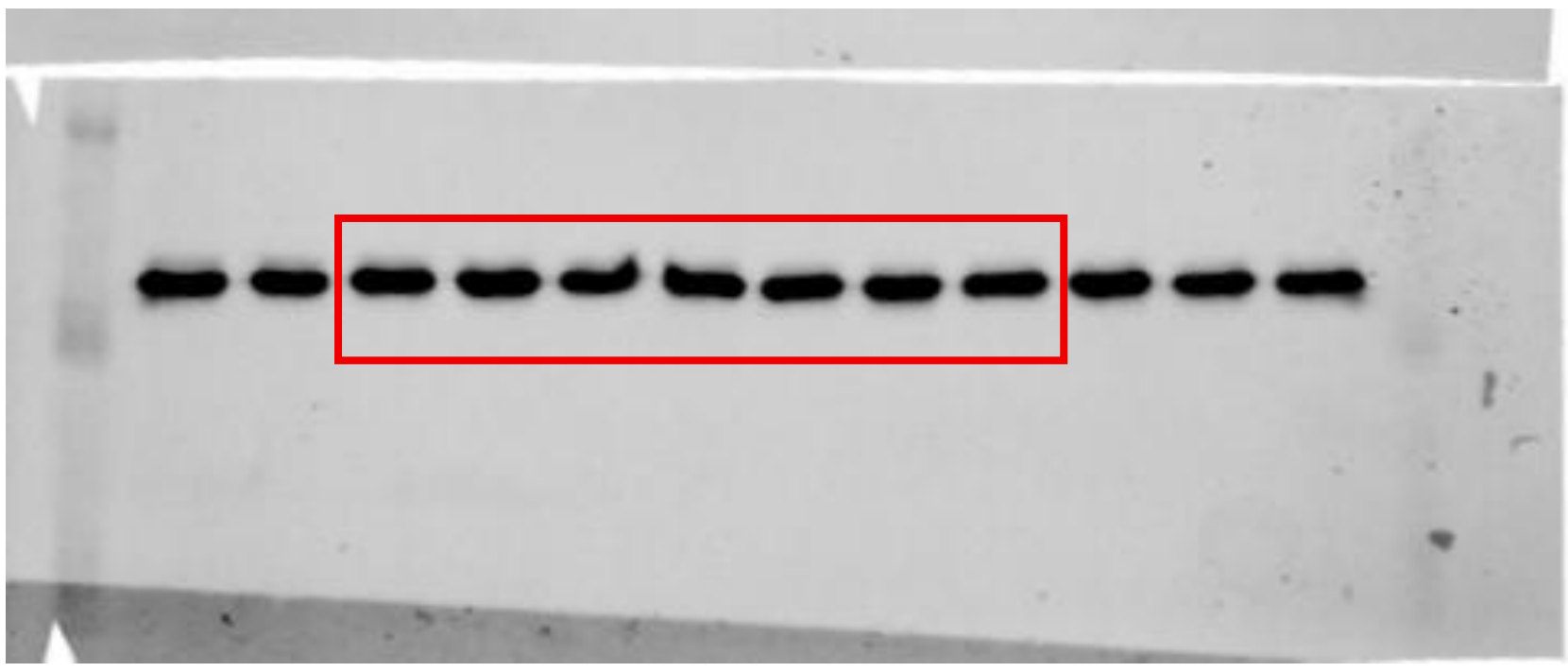

K562 MRAS-GTP

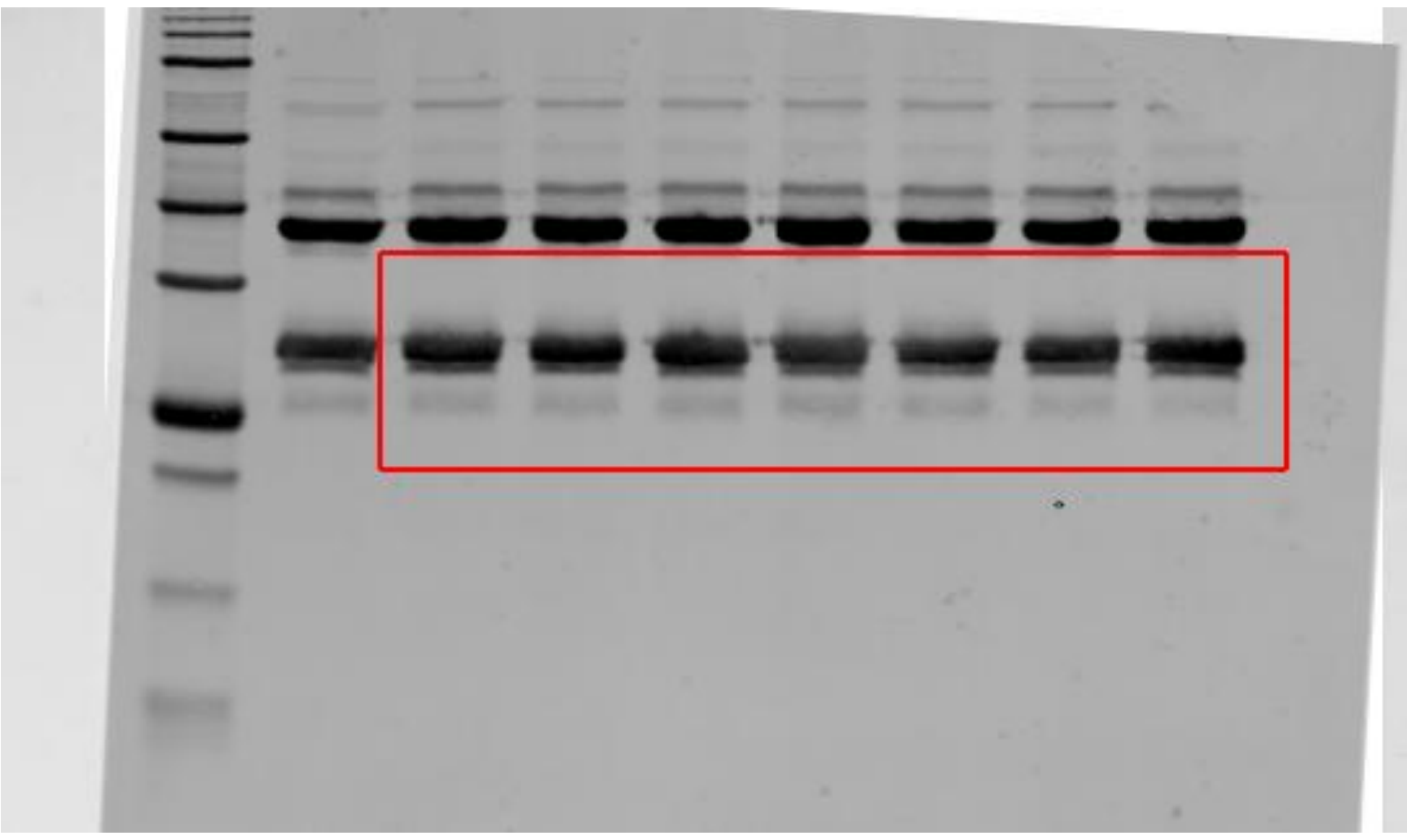

K562 MRAS

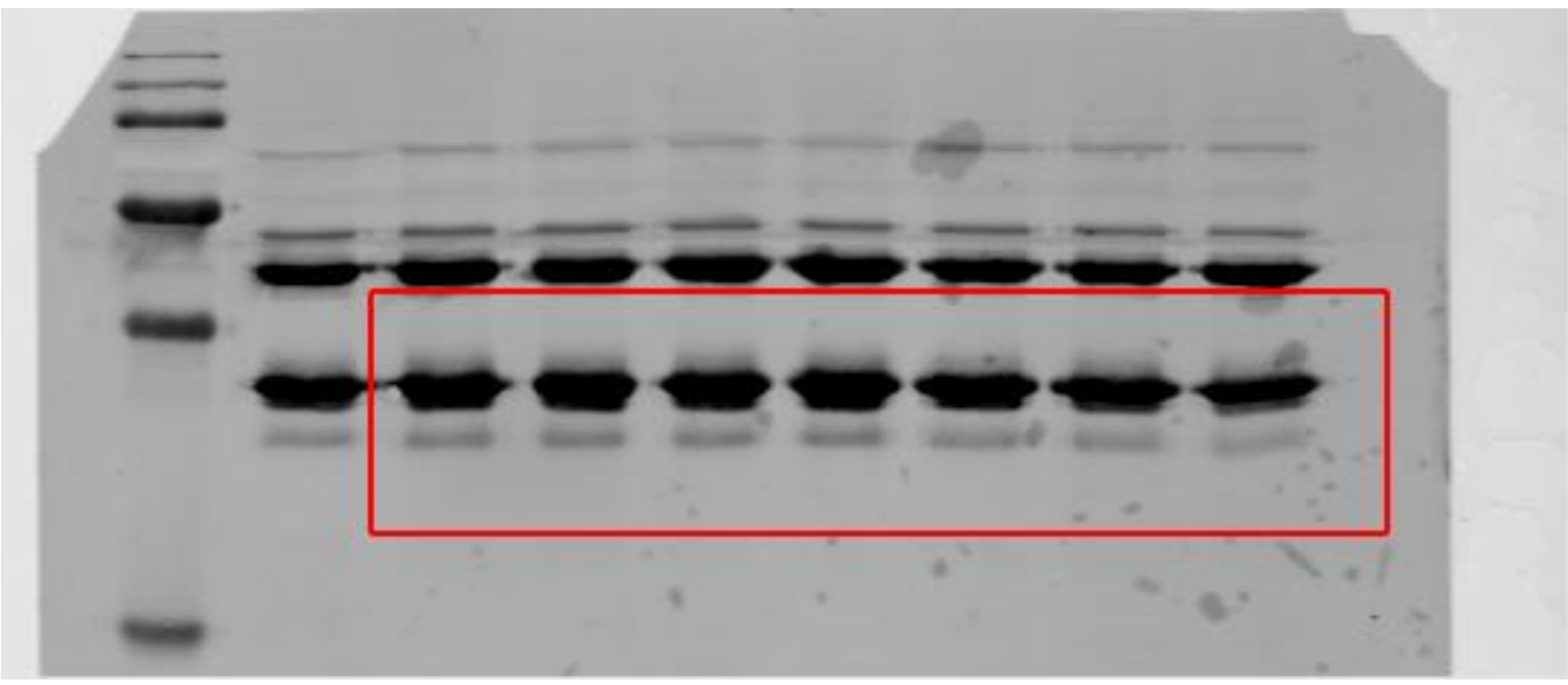

KCL22 MRAS-GTP

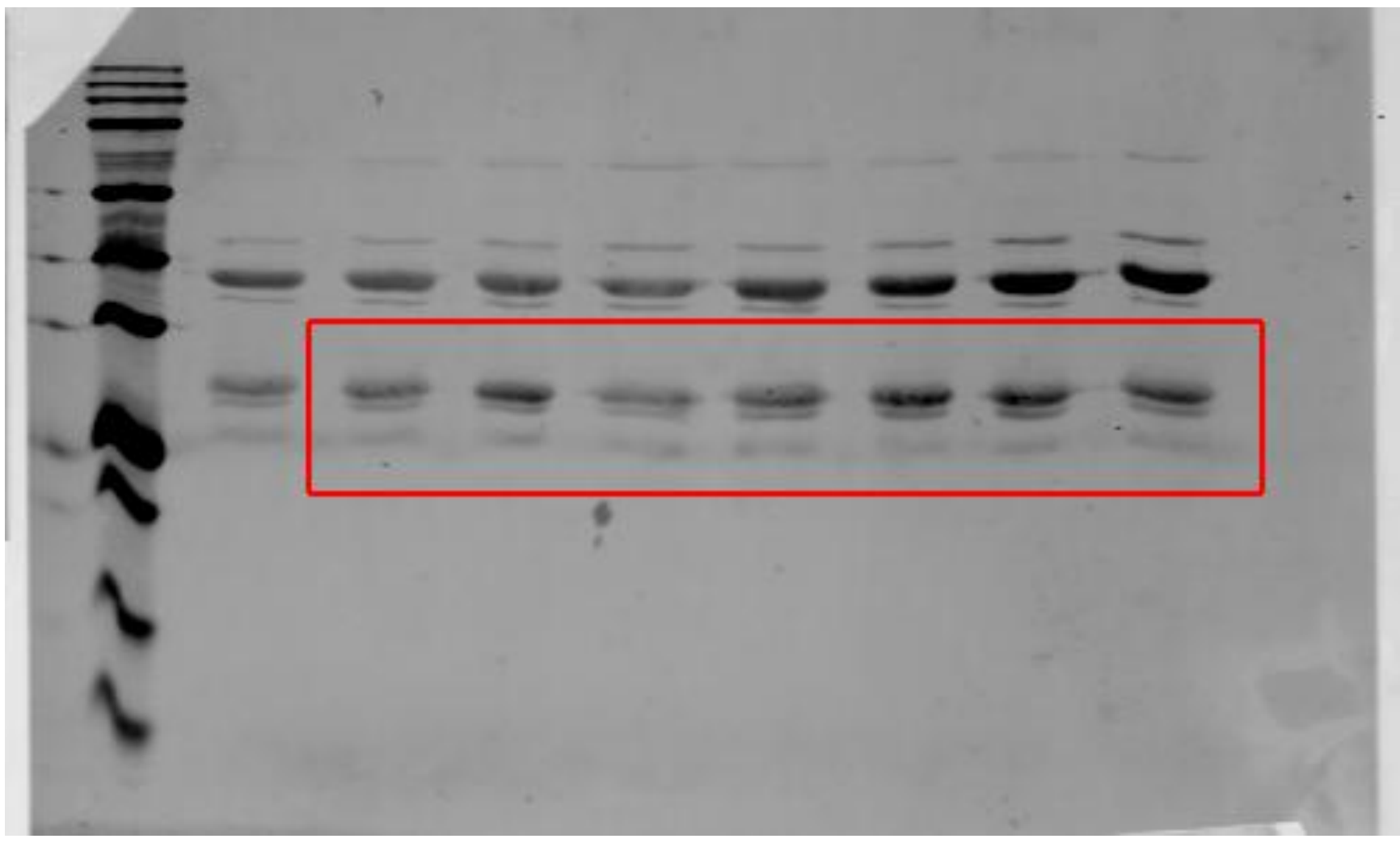

KCL22 MRAS

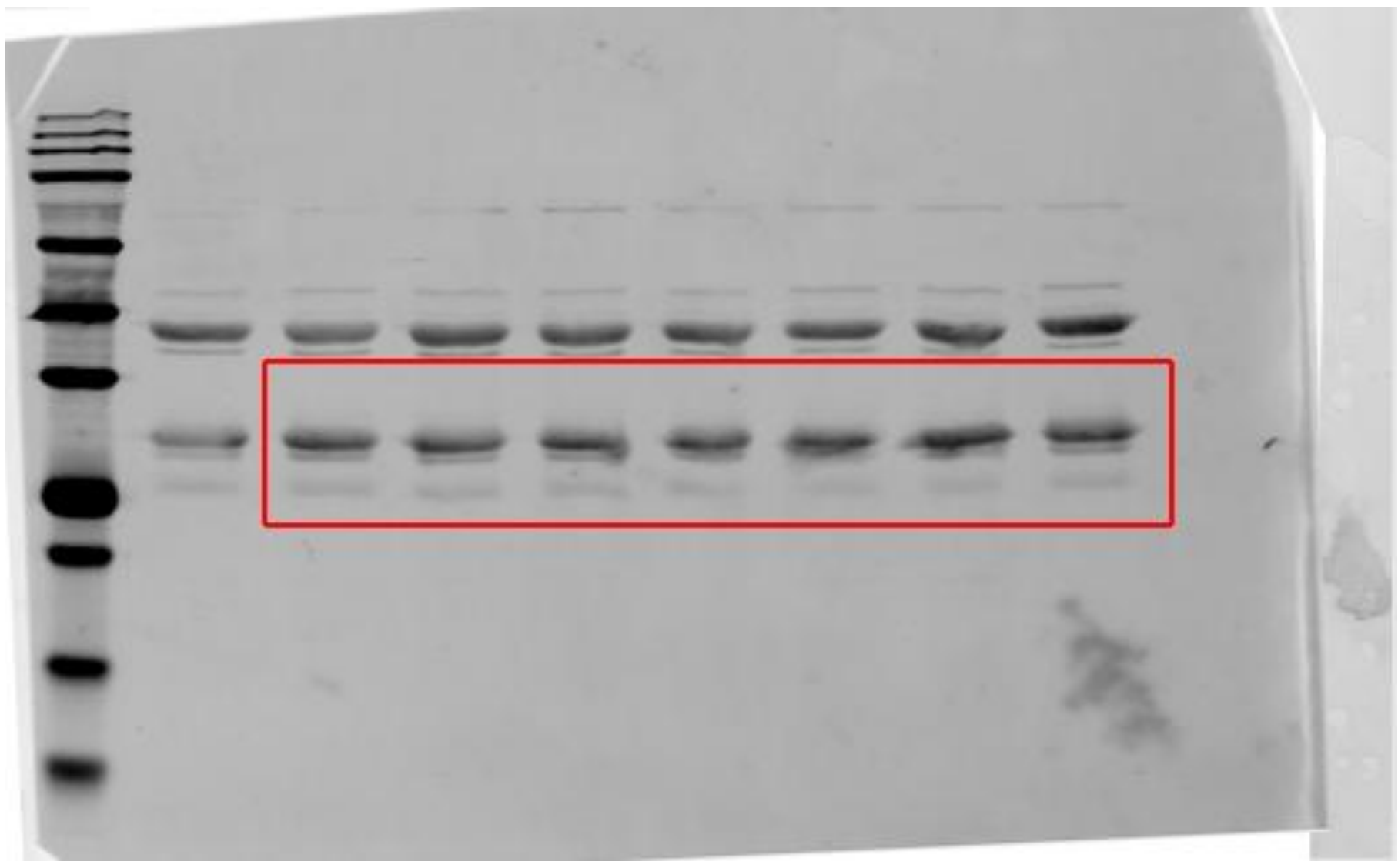

K562 pERK

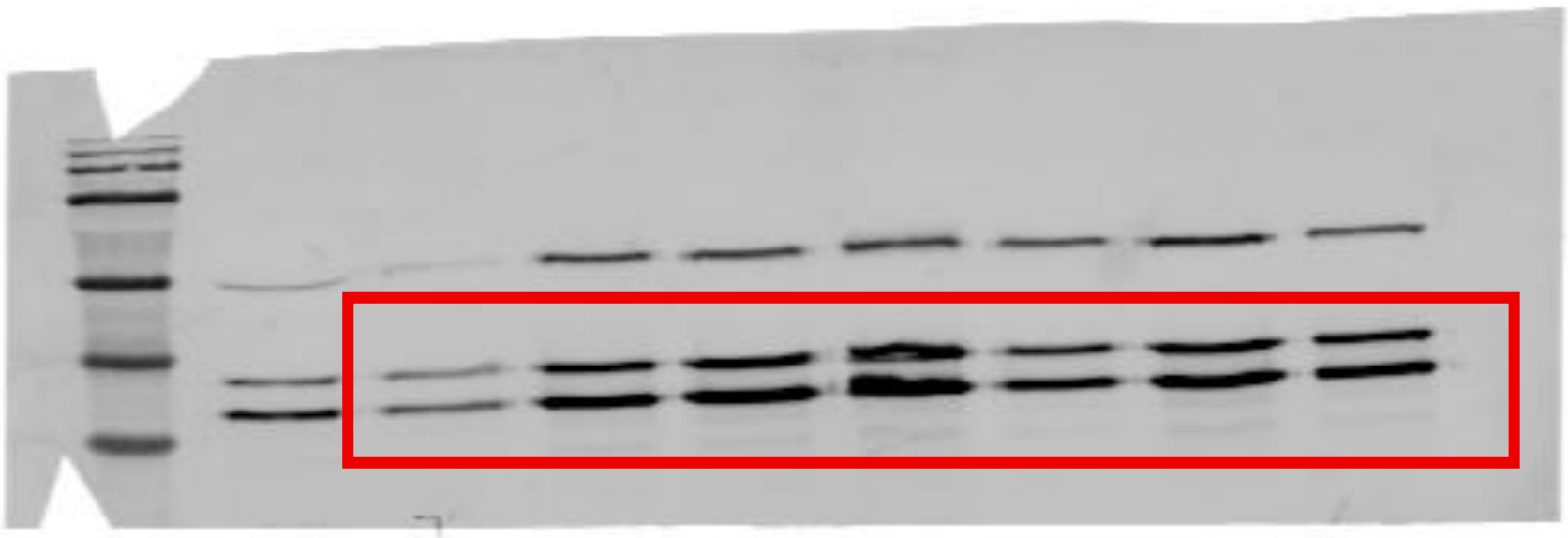

KCL22pERK

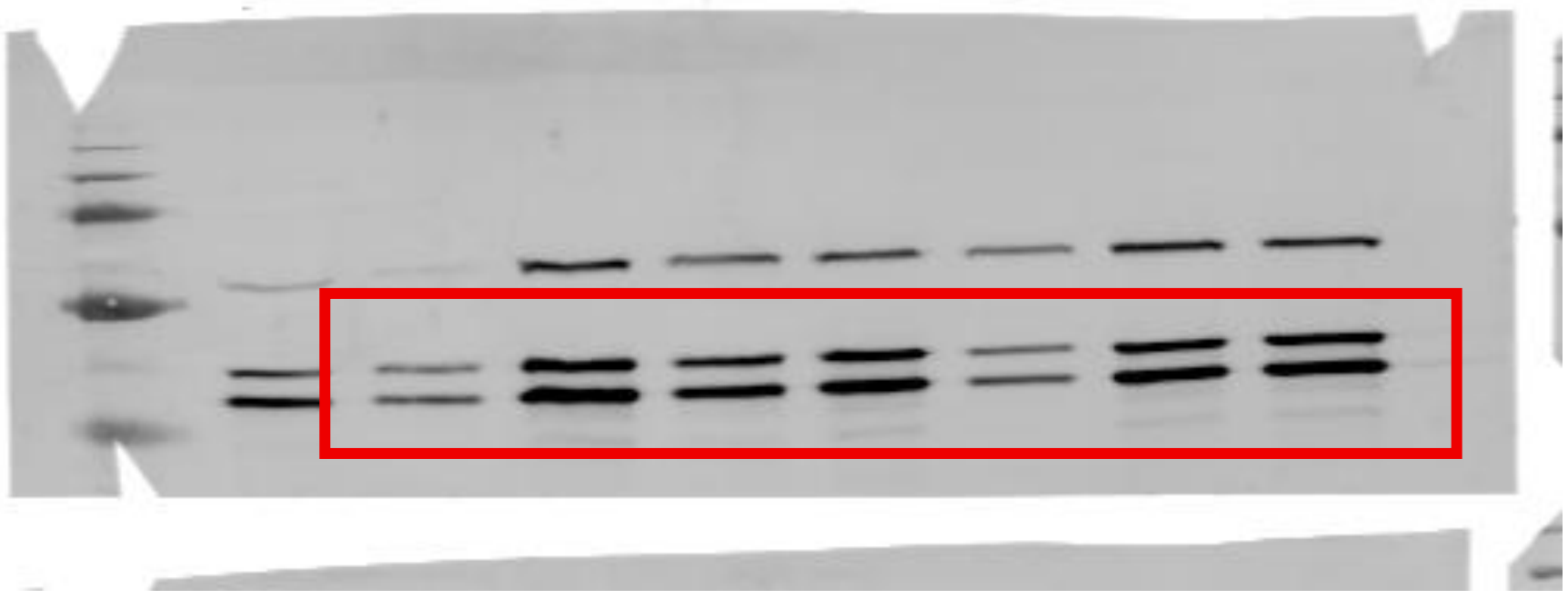

K562 pAKT

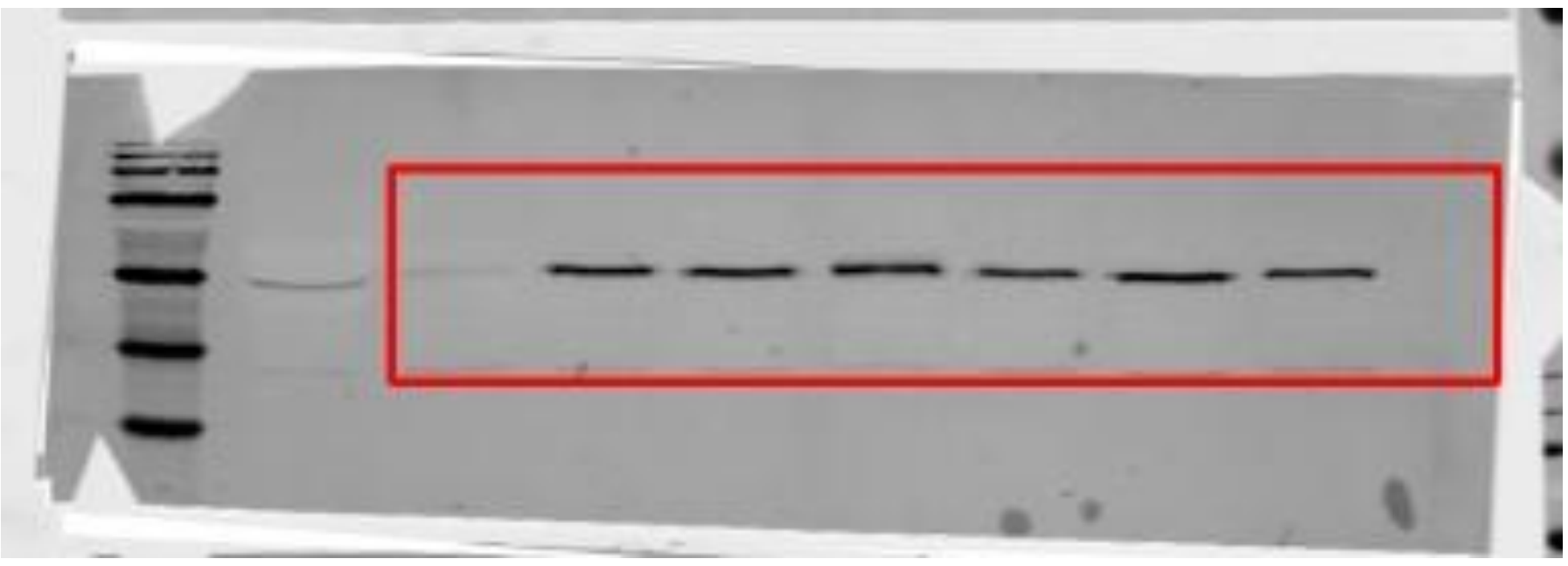

KCL22 pAKT

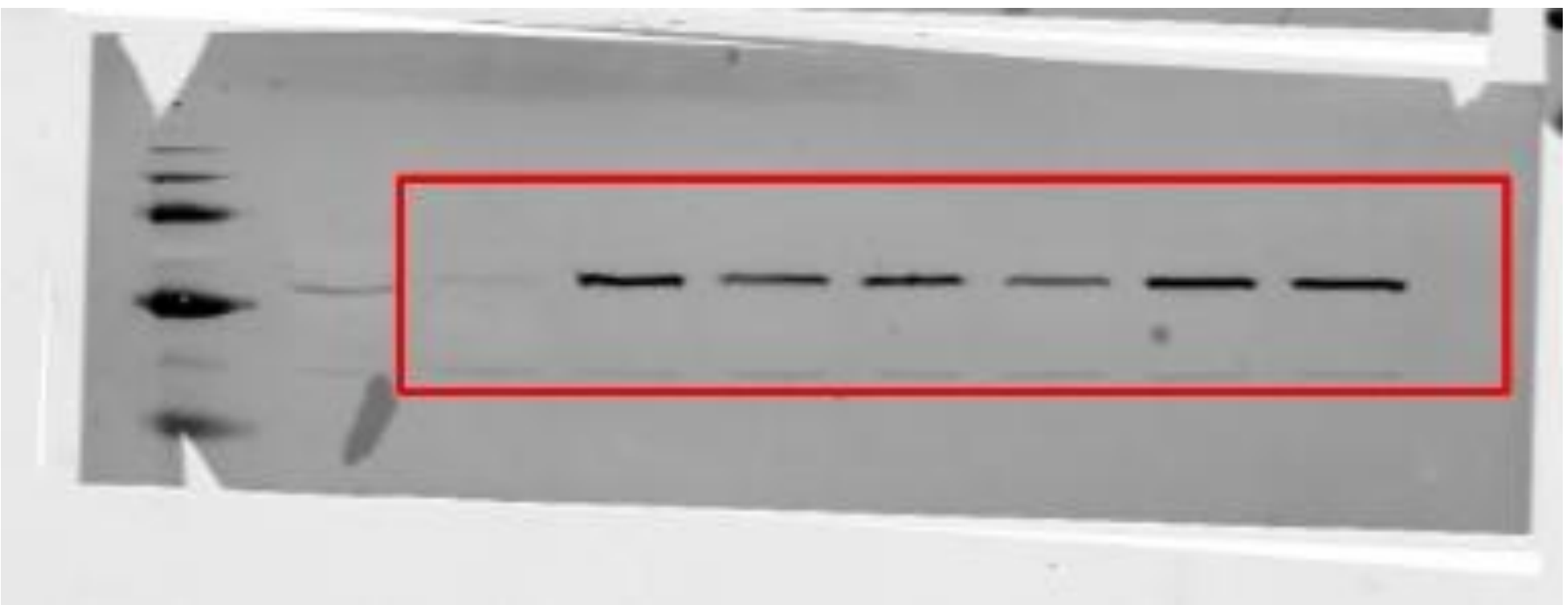

K562 AKT, ERK, TUB

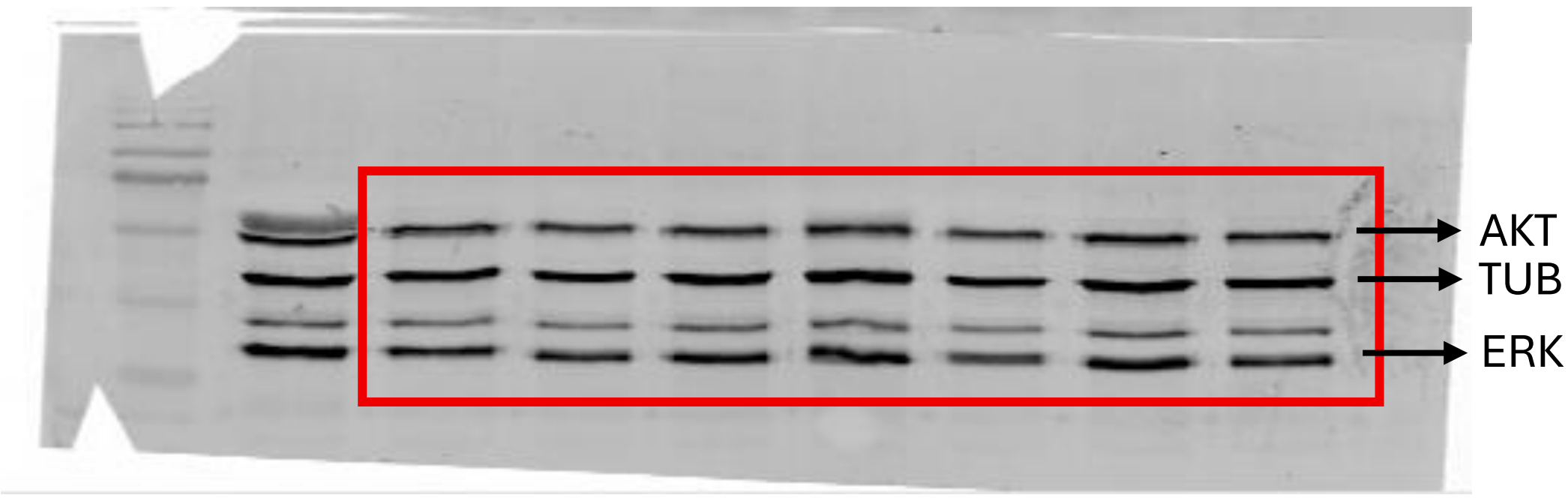

KCL22 AKT, TUB, ERK

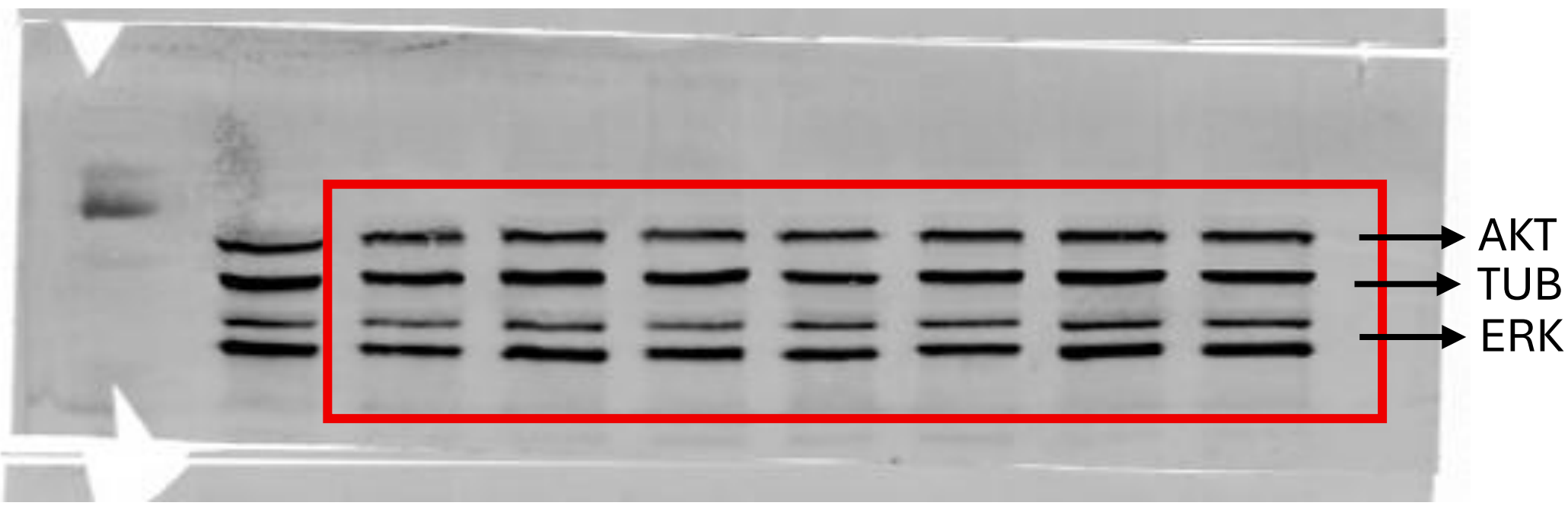

K562 SOS1

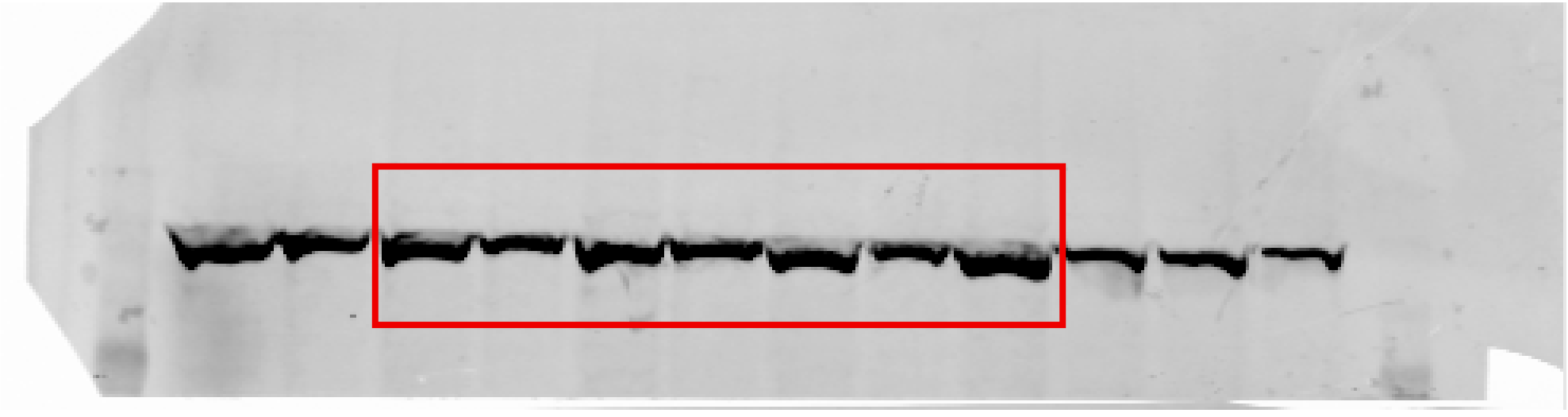

K562 SOS2

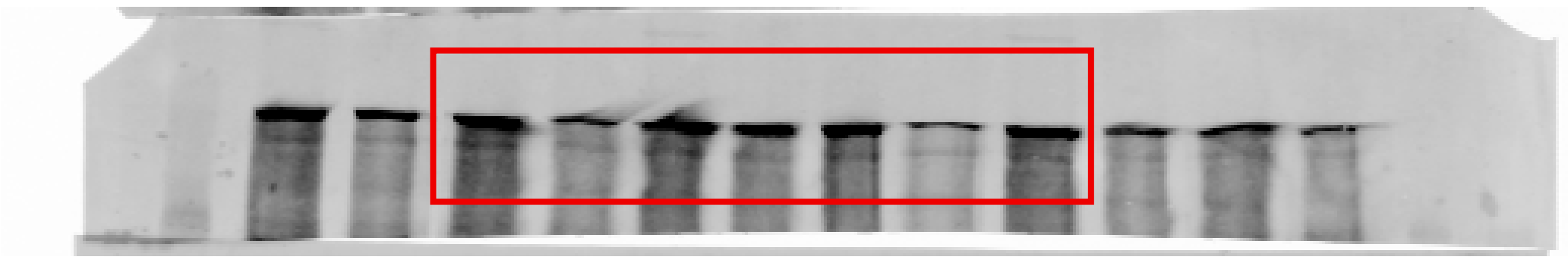

KCL22 SOS1

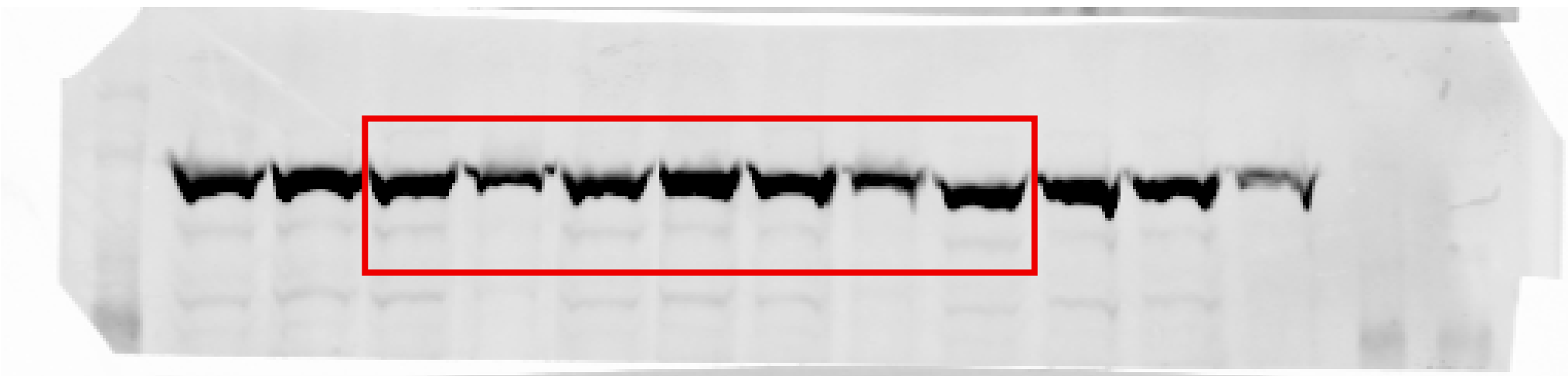

KCL22 SOS2

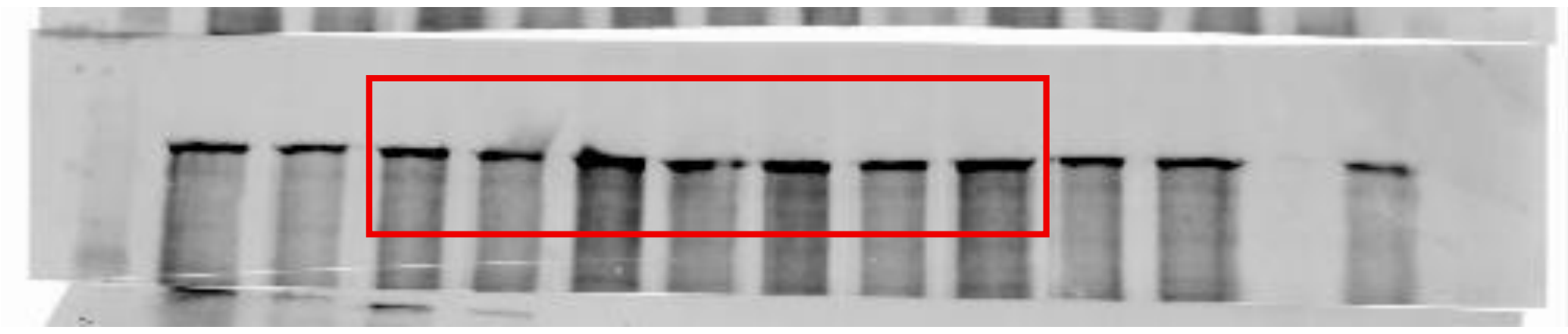

Figure 6b)

K562 pERK

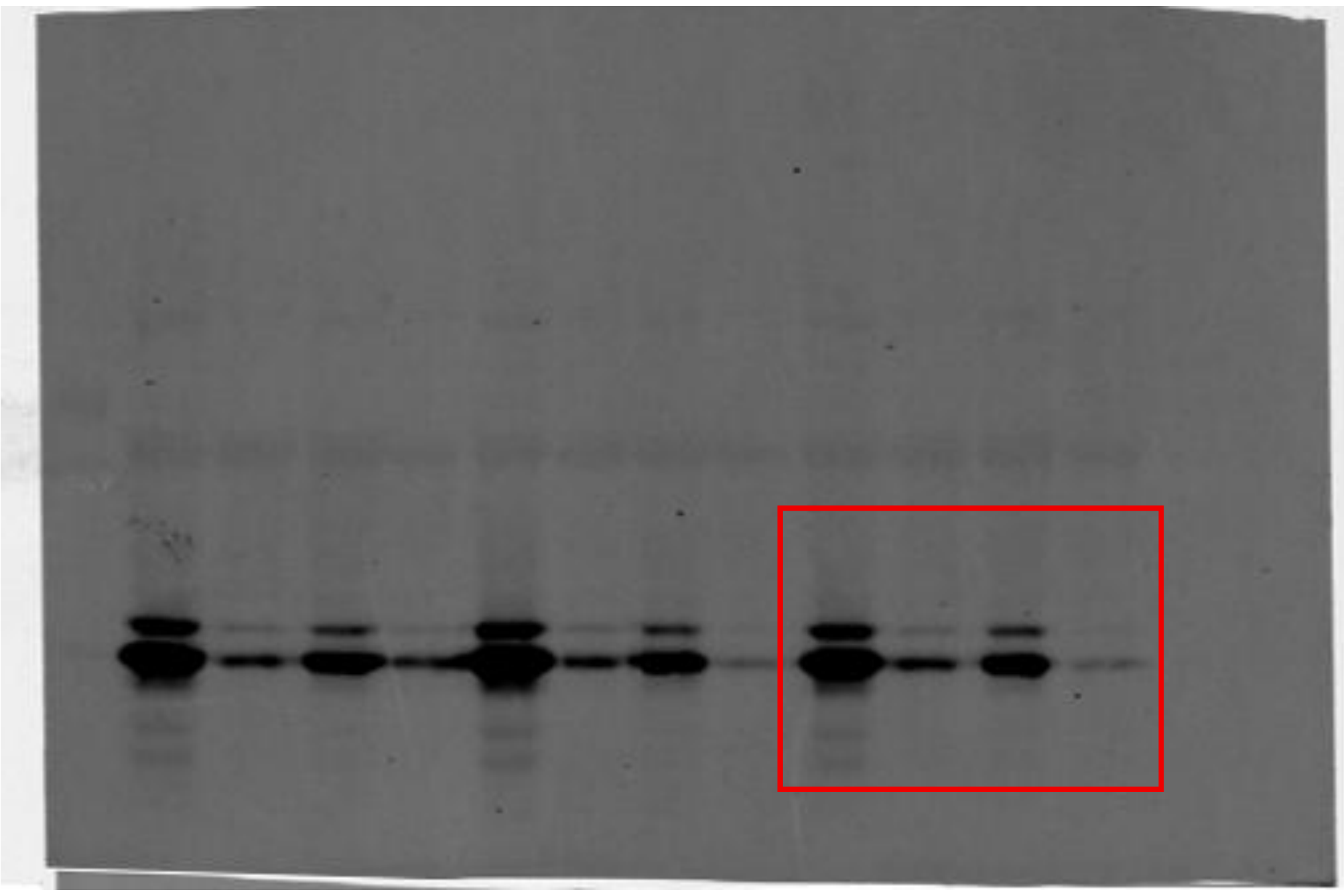

K562 ERK, TUB

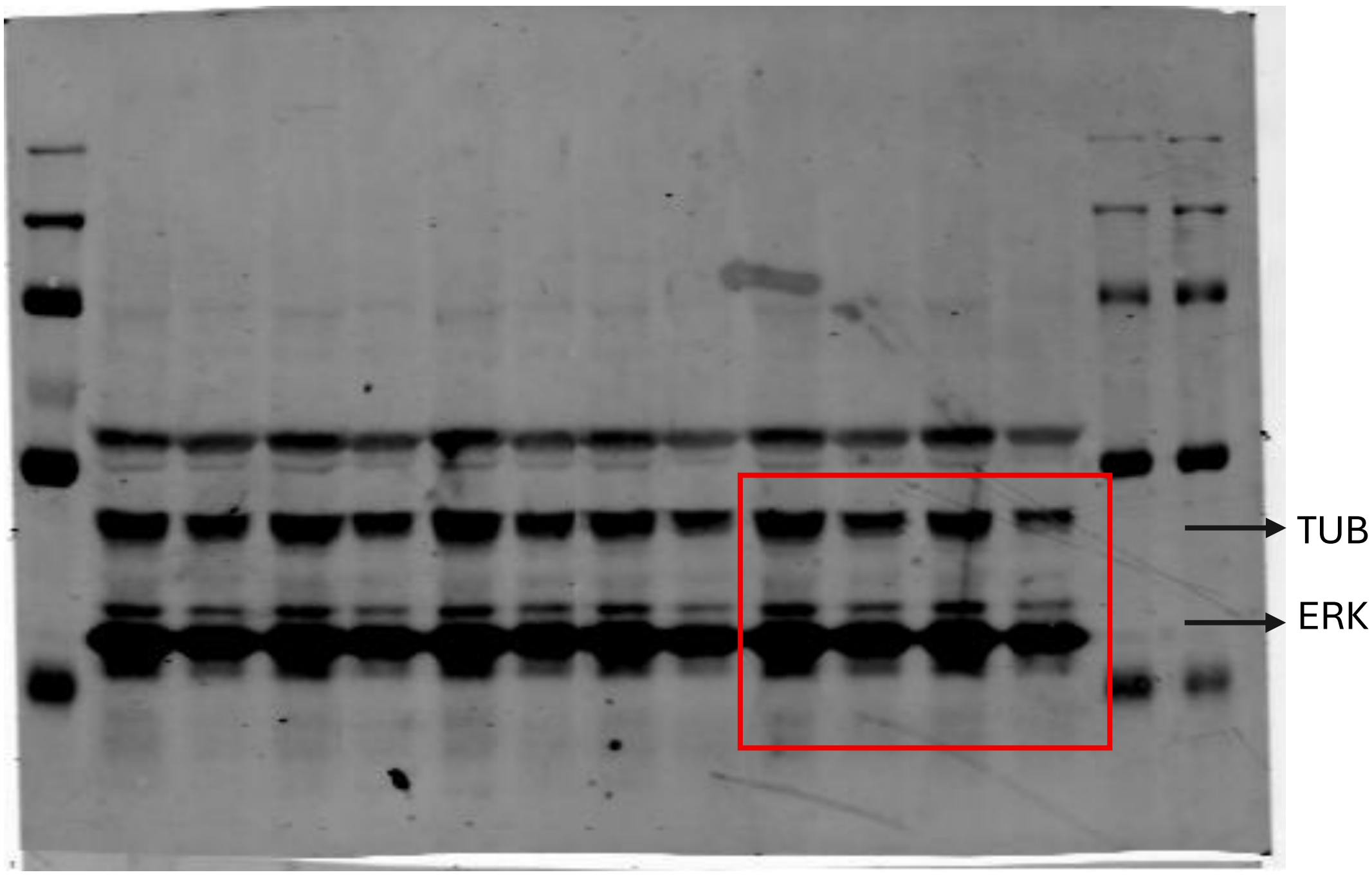

KCL22 pERK

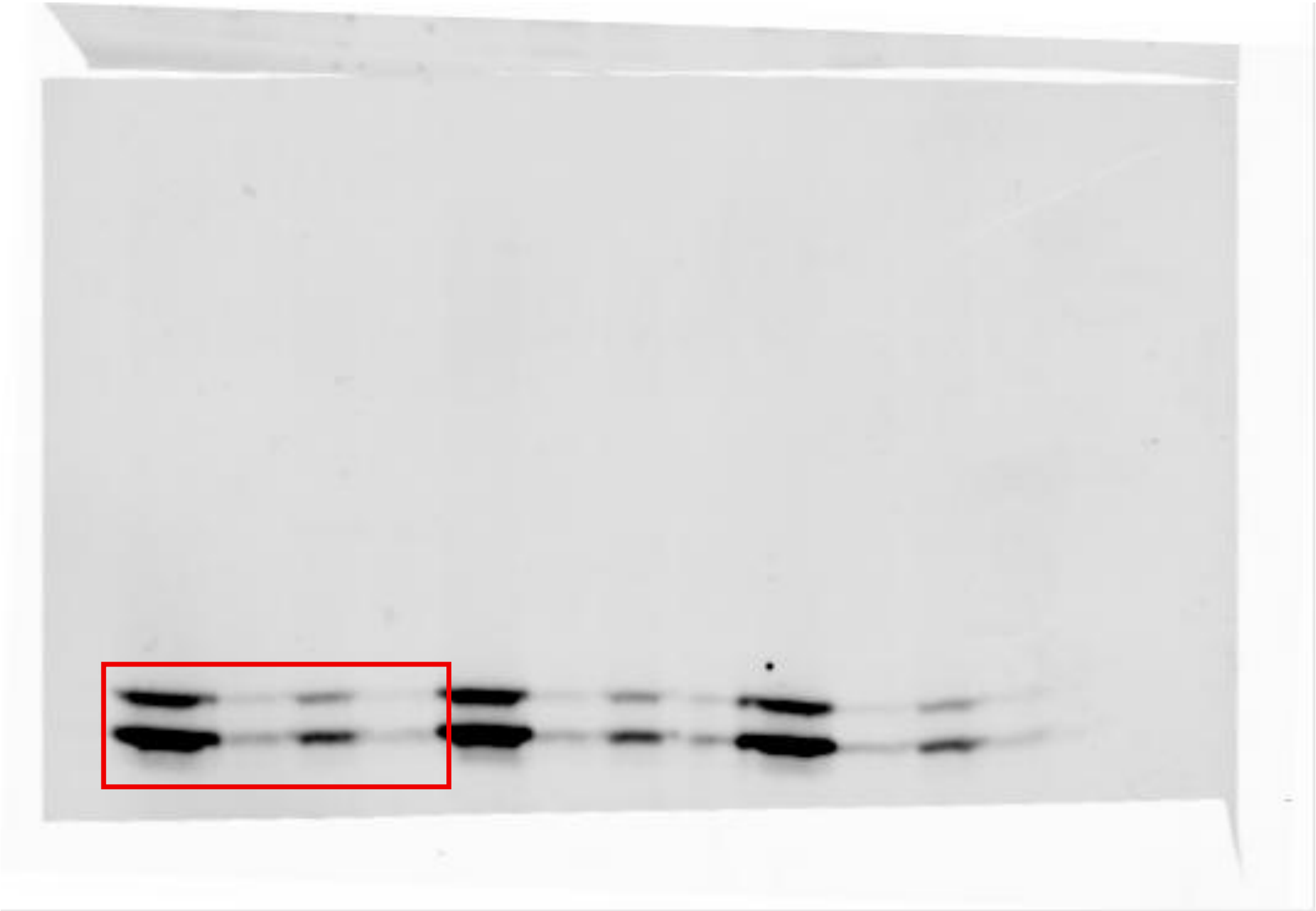

KCL22 ERK, TUB

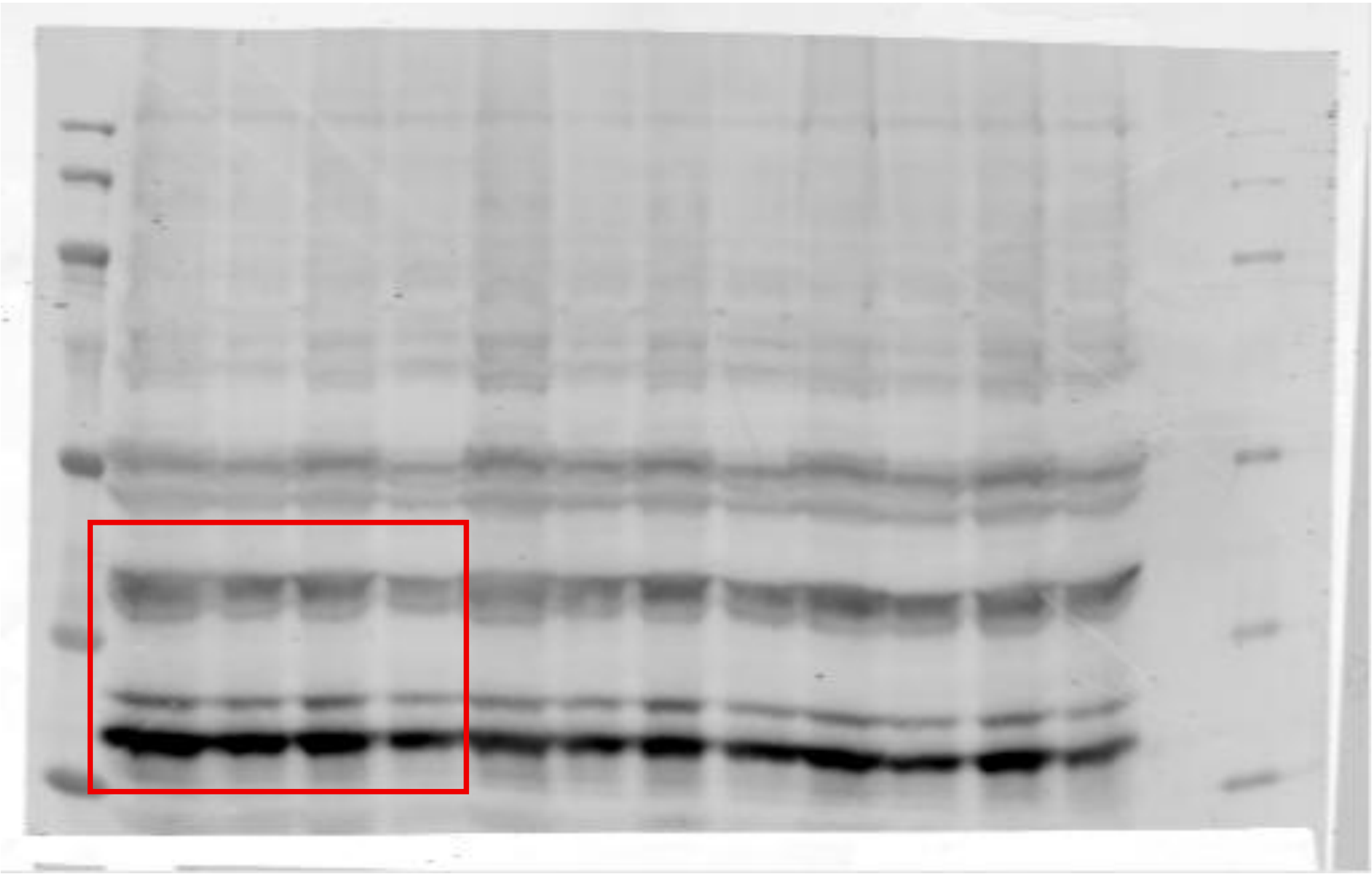

K562 pAKT

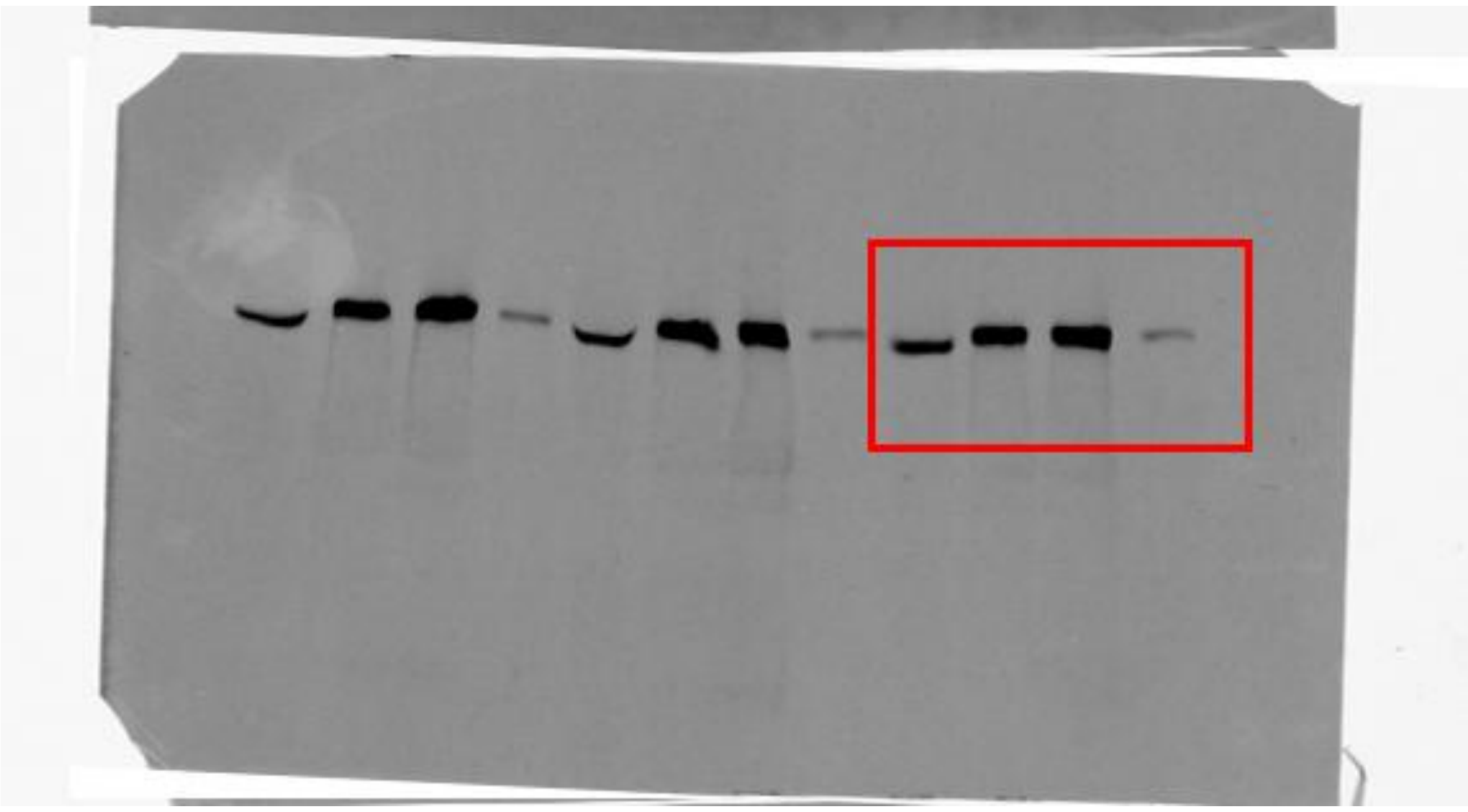

K562 AKT

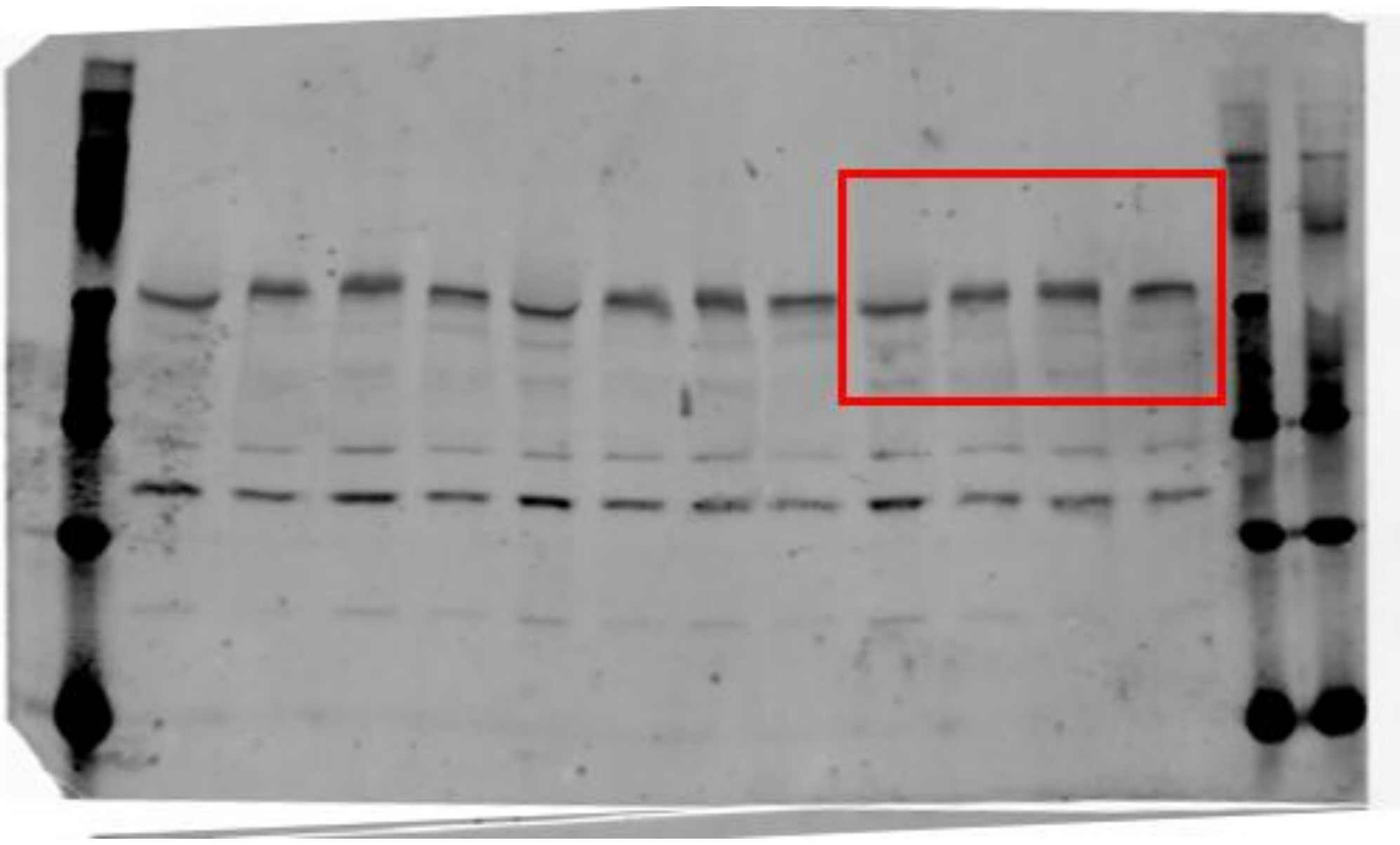

KCL22 pAKT,

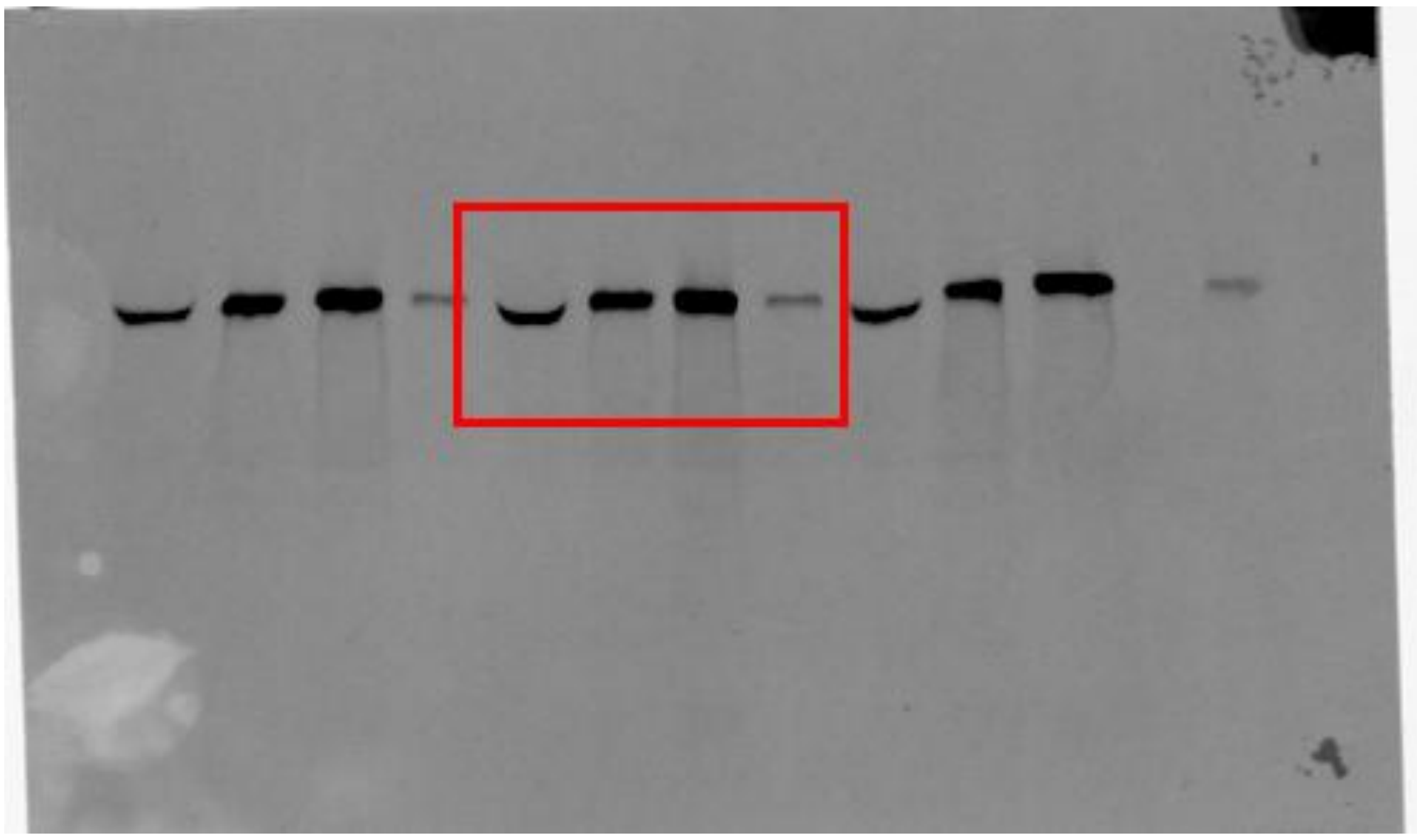

KCL22 AKT

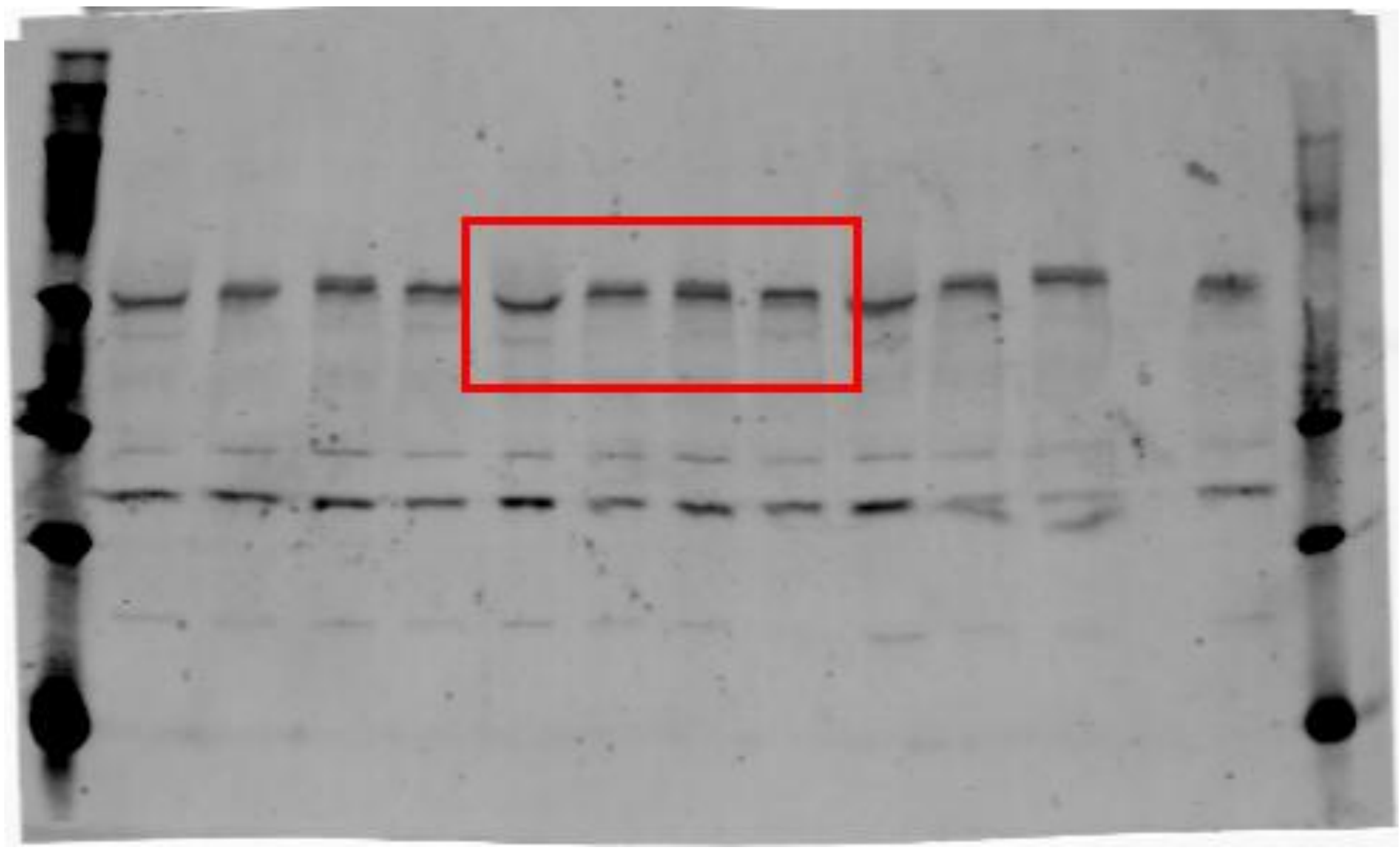

KCL22 TUB

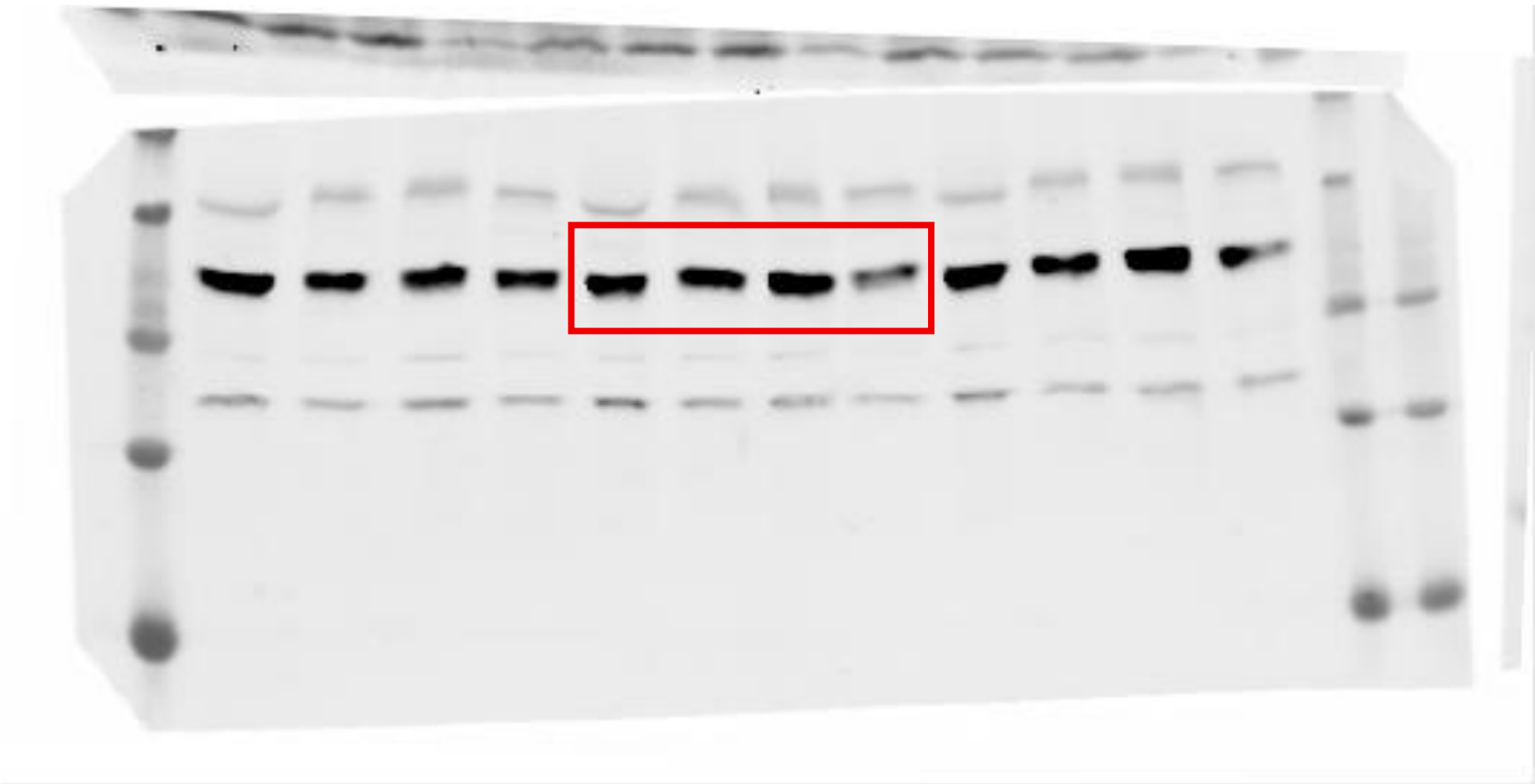

Supplement: Supplementary file 2 [file 41419_2026_9170_MOESM4_ESM.pdf]
